# Validation of an mHealth System for Monitoring Fundamental Physiological Parameters in the Clinical Setting

**DOI:** 10.3390/s24165164

**Published:** 2024-08-10

**Authors:** Filipe Martins, Elsa Fragoso, Hugo Plácido da Silva, Miguel Sales Dias, Luís Brás Rosário

**Affiliations:** 1 Instituto Superior Técnico, University of Lisbon, 1049-001 Lisbon, Portugal; 2 Pulmonology Department, Santa Maria University Hospital (CHULN), Santa Maria Local Health Unit, Av. Prof. Egas Moniz, 1649-028 Lisbon, Portugal; elsagcfragoso@gmail.com; 3 Pulmonology Clinic, Lisbon School of Medicine, University of Lisbon, 1649-028 Lisbon, Portugal; 4 Instituto de Telecomunicações, Instituto Superior Técnico, 1049-001 Lisbon, Portugal; 5 Department of Bioengineering, Instituto Superior Técnico, University of Lisbon, 1649-004 Lisbon, Portugal; 6 Lisbon Unit for Learning and Intelligent Systems (LUMLIS), European Laboratory for Learning and Intelligent Systems (ELLIS), 1049-001 Lisbon, Portugal; 7 Information Sciences and Technologies and Architecture Reasearch Center (ISTAR), University Institute of Lisbon (ISCTE-IUL), 1600-189 Lisbon, Portugal; miguel.dias@iscte-iul.pt; 8 Cardiology Department, Santa Maria University Hospital (CHULN), Lisbon Academic Medical Centre, 1649-028 Lisbon, Portugal; lsrosario@medicina.ulisboa.pt; 9 Centro Cardiovascular, Faculdade de Medicina, University of Lisbon, Av. Prof. Egas Moniz, 1649-028 Lisbon, Portugal

**Keywords:** agreement analysis, Bland–Altman plot, body temperature, heart rate, peripheral oxygen saturation

## Abstract

The aim of this work was to validate the measurements of three physiological parameters, namely, body temperature, heart rate, and peripheral oxygen saturation, captured with an out-of-the-lab device using measurements taken with clinically proven devices. The out-of-the-lab specialized device was integrated into a customized mHealth application, e-CoVig, developed within the AIM Health project. To perform the analysis, single consecutive measurements of the three vital parameters obtained with e-CoVig and with the standard devices from patients in an intensive care unit were collected, preprocessed, and then analyzed through classical agreement analysis, where we used Lin’s concordance coefficient to assess the agreement correlation and Bland–Altman plots with exact confidence intervals for the limits of agreement to analyze the paired data readings. The existence of possible systematic errors was also addressed, where we found the presence of additive errors, which were corrected, and weak proportional biases. We obtained the mean overall agreement between the measurements taken with the novel e-CoVig device and the reference devices for the measured quantities. Although some limitations in this study were encountered, we present more advanced methods for their further assessment.

## 1. Introduction

Cardiovascular diseases are a major cause of mortality worldwide and are the leading cause of hospital admissions in Europe and the U.S. Over 60 million premature deaths have been attributed to cardiovascular diseases in Europe annually, and device-based prevention and treatment can help tackle this pandemic [1]. The current annual expenditure for cardiovascular diseases is estimated to be over EUR 200 million in Europe, accounting for the largest proportion of the total health care expenditure, at over 16% [2].

Respiratory diseases also have a major impact worldwide. In 2017, almost 545 million people had chronic respiratory disease (7.9% of the world’s population) and that figure corresponded to a global increase of 39.8% compared to 1990 [3]. Chronic obstructive pulmonary disease (COPD) was the third leading cause of death in 2020, following ischemic heart disease and stroke [4]. Lower respiratory tract infections (namely, pneumonia) and lung cancer were the fourth and sixth leading causes of mortality in the same year. Further impacts on society are also observed in days lost due to disease-related disability. In fact, after analyzing all ages and levels of country income, four of the “big five” lung diseases are among the top 20 causes of disability-adjusted life years (DALY): lower tract respiratory infection is the 4th, COPD is the 6th, tuberculosis is the 12th, and lung cancer is the 17th leading cause [4]. Altogether these diseases have a major contribution to premature death and disability. Among the tools and strategies used to monitor and treat respiratory diseases, telemedicine is among the most promising, bringing the patient closer to the healthcare team. By providing the ability to perform remote monitoring, tele-spirometry, and home-based tele-rehabilitation, it is already a game-changer in modern clinical practice [5].

Multiple physiologic or vital parameters, such as heart rate, respiratory rate, and peripheral oxygen saturation, can be used to evaluate the diagnosis, outcome, and severity of cardiovascular and pulmonary diseases. In the intensive care setting and during hospitalization, these parameters are used to monitor disease progression, support therapeutic decisions, and determine the length of hospital stay. Ambulatory patients with chronic pulmonary and cardiovascular diseases could benefit from similar evaluation if an outpatient monitoring system was available and reliable. Chronic diseases, such as heart and chronic obstructive pulmonary diseases, that involve acute decompensations may benefit from outpatient monitoring to make therapeutic decisions and avoid hospital admissions. Proper outpatient monitoring may also allow for outpatient care after hospitalization and reduce the hospital length of stay.

The e-CoVig device, previously developed within the scope of the AIM Health project and described in [6], can be used to directly measure body temperature and peripheral oxygen saturation (SpO_2_) and indirectly to calculate heart rate (HR). A previous evaluation in healthy volunteers had promising results in the normal physiological range for the three parameters [6,7]. e-CoVig has an architecture based on the ESP32 chipset, coupled with two off-the-shelf biomedical sensors, namely, a pulse oximeter by Maxim Integrated (San Jose, CA, USA) and an Infra-Red (IR) thermometer by Melexis (Ypres, Belgium). The device has Bluetooth connectivity to communicate, e.g., with a smartphone, as is the case in this work.

This study was designed to compare the e-CoVig-determined physiologic parameters with those determined with current certified devices in a clinical setting within the pathologic range of variation by means of agreement data analysis.

## 2. Materials and Methods

**Figure 1:**
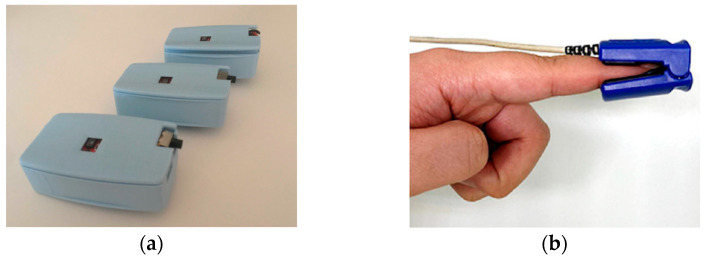
Photos of device and reading sensor. (**a**) e-CoVig device, extracted from [6]; (**b**) finger sensor with identical application to the one used, extracted from [8].

Figure 1 depicts the e-CoVig device; an ESP32 module is used as the microcontroller, coupled with a Maxim MAX30101 and a Maxim MAX32664 by Maxim Integrated (San Jose, CA, USA) for cardiovascular measurements, and with a Melexis MLX90615 sensor by Melexis (Ypres, Belgium) for body temperature measurement (we refer the reader to the Supplementary Material for the datasheets with more information on these modules). From the MAX30101 and MAX32664, oxygen saturation and heart rate values are obtained using reflective photoplethysmography; a confidence level indicator and a finger placement status are also computed.

With the MLX90615, body temperature is measured using an IR-based approach; the sensor also enables ambient temperature measurement. A moving average filter (N = 3) is implemented on the device to smooth temperature measurements, and a linear regression is used to estimate the body temperature; the parameters for this regression are described in [6]. The device measures SpO_2_, HR, and temperature two times per second and, once a Bluetooth connection is established, all the measurements are continuously streamed via Bluetooth Serial in JavaScript Object Notation (JSON) format to the smartphone, as illustrated below:

{“HR”: 72, “Confidence”: 100, “SpO_2_”: 99, “Status”: 3,

“Object Temperature”: 33.0, “Ambient Temperature”: 15.1}

From the operator’s point of view, this device has a very fast learning curve. To use e-CoVig, the healthcare professional needs to have a smartphone with the AIM Health application installed, a Bluetooth connection available, and Internet access for data transfer. The only steps required are to launch the app, start a new measurement, and then turn on the e-CoVig device. The device automatically connects to the app. Once the measurements are underway, the readings appear on the phone screen in the app. Successful readings are saved by the app and transferred to a remote server. Poor internet access at the time of the measurements does not preclude the actual readings collection, since the app saves the data and transfers the data automatically as soon as internet access is available.

### 2.1. Data Collection

The AIM Health Study was approved by the Centro Médico Académico de Lisboa/Hospital de Santa Maria Ethics Committee (Ref. 132/22) including its informed consent form. The Institution Data Protection Officer was informed of the study procedures.

The health care professional (HCP) in charge of the data collection had two sets of a mobile phone with the AIM Health app and an e-CoVig device and was responsible for ensuring their proper functioning; technical support for the AIM Health app was provided through direct contact with the team whenever necessary. 

The measurements were performed on patients admitted to the respiratory intensive care unit of a pulmonology department in two distinct periods of time: from 17 October 2021 to 16 February 2022 and from 24 March 2023 to 15 June 2023. A total of 86 measurements were taken. In each instance of data collection, the readings obtained through the e-CoVig device were directly transferred to the connected AIM Health app on the mobile phone. Immediately after successfully obtaining readings with the AIM Health app, the measurements were repeated using certified medical devices in the intensive care unit, and the values obtained were registered directly in the app by the HCP. Temperature measurement was taken at the forehead with the experimental e-CoVig device and from the ear canal with the reference thermometer. The different measurement techniques for each device implied measurement at different locations for this parameter. Heart rate and peripheral oxygen saturation were both obtained from contact with a finger. The same finger was used to collect the data with both devices. Once all the measurements were taken and registered, the HCP provided data on the age, sex, and perceived quality of the measurements taken in the AIM Health app. After completing the form, the data were transferred to the remote server by the app. All the measurement episodes were registered by the HCP in a spreadsheet for validation purposes.

The standard clinically validated devices used to collect the data were as follows: (1) For body temperature, a Welch Allyn Thermometer (Braun Thermoscan PRO 6000) by Welch Allyn (Skaneateles Falls, New York, NY, USA) was used, which had an error margin of ±0.2 °C. (2) For heart rate and peripheral oxygen saturation (SpO_2_), a Spacelabs Multiparamether Monitor, model 94267 PRO-19370030B by Spacelabs (Snoqualmie, Washington, DC, USA), was used, which had an error margin for heart rate ±3 beats per minute (BPM) (Quality Institute, ISQ and Portuguese Institute of Accreditation, IPAC report) and, for SpO_2_, ±4% (specification ISO 80601-2-61:2017 see [9] for more details). Both devices had updated quality certification reports, ensuring accurate measurements during the entire study period.

Participants agreed to take part in the AIM Health study and signed the informed consent form before any measurement was taken.

### 2.2. Statistical Methodology

The clinically validated e-CoVig readings were compared by means of agreement analysis, based on Lin’s concordance correlation and on Bland–Altman plots, to which we added exact confidence intervals for the limits of agreement. The measurements were obtained sequentially with the devices for each participant and for each prespecified physiological variable: body temperature, heart rate and peripheral oxygen saturation.

In medical studies, comparisons of new measurement equipment with established equipment is needed to prove they are equivalent for clinical use. Correlation studies are notably used to assess such comparison; however, correlation measures the linear relationship between one variable and another and not the difference between them, in the sense that the variables may very well correlate but may disagree significantly in value, so correlation measures are not recommended as a method for such analysis of comparability. 

Bland and Altman proposed a method to analyze the agreement between measurement quantities collected from two different devices, which sums up to producing a plot where the differences, the means of differences, and the limits of agreement between the measurements from the two devices can be analyzed. For each pair of measurements, their difference is plotted against their respective mean, a horizontal reference line is added, the mean of the differences and other two horizontal lines are added; typically, mean difference ± 1.96 times the standard deviation of the differences, is taken, which are known as the limits of agreement (LOA) [10,11]. Bland–Altman agreement analysis has been extensively used to evaluate the agreement between measurements from two different devices and allows the identification of any systematic differences between measurements, i.e., fixed bias, as well as any possible discrepancies in the differences along the range of measurements, i.e., proportional bias, and the identification of possible outliers in the data. 

The limits of agreement provide a way of assessing the range of variability between two types of measurements; however, the LOA is sample-size-dependent, so it becomes a biased estimate of the population’s LOA. Therefore, the inclusion of confidence intervals for the LOA should be standard practice, describing the range for the estimated LOA to lie in the population LOA, with a probability or confidence level of (typically) 95% [12]. For this reason, we included confidence intervals for the LOA. We considered exact confidence intervals for the LOA, as proposed in [12], which are more relevant for small sample sizes but also appropriate and useful for any sample size, as opposed to the approximate classical ones presented in [11]. 

The Bland–Altman method of analysis is based mainly on two assumptions: (1) Normally distributed differences between measurements, according to which a value of 1.96 represents the z-score at a significance level of 5%, which guarantees that approximately 95% of the data lies between the limits of agreement; (2) a lack of proportional bias, indicating that the two different measurements agree equally through the range of measurements; that is, the limits of agreement does not depend on the range of measurements. 

To address the normality of the differences, we considered the Kolmogorov-Smirnov (K-S) and Shapiro-Wilk (S-W) tests, whose null hypothesis is that the differences follow a Normal distribution. However, even in the case of a non-normality test result, if approximately 95% of the data points lie within the LOA, it is still valid to proceed with Bland–Altman analysis [11]. The K-S test was used because taking the difference in the means removes a significant amount of the variability in paired data, which underlies the assumption that the differences are likely to follow a Normal distribution. However, if the distribution of the differences is skewed or has long tails, the assumption of normality may not be valid; for this reason, we also considered the S-W test, which does not assume the normality of the differences. 

To measure the agreement correlation between the paired data measurements, we used Lin’s concordance correlation coefficient, which is a measure of inter-rater agreement. It measures how close the data points are to the line of best fit and how far they are from the line of equality or total agreement. Lin’s coefficient enabled more accurate estimates of the agreement between the measurements from the two devices, as opposed to Pearson’s correlation coefficient, which measures a linear relationship but fails to detect any departure from the 45-degree line through the origin, i.e., the line of equality or total agreement between the paired measurements [13]. 

In addition to the agreement analysis, we also considered the acceptance limits between measurements with regard to the admissible physiological limits of agreement in each measured quantity. For body temperature, the standard acceptable limit is ±0.5 °C, that of heart rate ranges from 60 to 100 BPM in mild conditions and at rest [14], and the clinically allowable error is ±5 BPM. Oxygen saturation ranges from 92 to 100% at rest [15], with an acceptable error limit of ±4%. We have to point out that these limiting error values are standard for any type of measuring equipment, in particular for the certified equipment used in the ICU (clinically validated). With the e-CoVig device being a low-cost mobile device, we decided to add one more acceptance limit for each quantity. We also considered a limit of ±1 °C for body temperature, a limit of ±8 BPM for heart rate, and a limit of ±6% for oxygen saturation.

In order to determine the possible existence of systematic additive errors in the differences of means of the measurements, we used the paired t-test and Wilcoxon signed-rank tests, whose null hypothesis in the present context was that the difference in the means in the measurements was zero. The paired t-test assumes the normality of the differences and the equality of the variance in the samples collected from both the e-CoVig and standard devices. In case of difference in variances, we used the Welch t-test. The Wilcoxon test was used in the case of non-normally distributed data (the case in which the differences failed the K-S normality test). To test for proportional bias, we performed a linear regression between the means and the differences and calculated the respective coefficient of determination, which, in this context, represents the proportion of the variance in the differences that is explained by the range of measurements. If zero or close to zero, it means there is none or a very small multiplicative or proportional effect of the range of measurements on the differences. These types of errors are typically constant or proportional to the true value, the clinically validated value, and should be eliminated as they may compromise the overall accuracy of the measurements, producing biased results in relation to validated ones. 

To assess the equality of variances between the independent samples obtained from the readings of the devices, we used the Levene test, whose null hypothesis is that the variance from the two independent samples is equal. This test is not very accurate for distributions that deviate from a Normal distribution, and three location measures were considered in the evaluation of the Levene test statistics due to existence of skewness and tails in the sample distributions: (1) in case of a symmetric distribution, the mean is used; (2) in case of skewness, the median is used; (3) in case of long tails, the trimmed mean is used. 

In this study, to assess the conclusiveness of the tests of the differences in the means between the independent samples, we calculated their respective statistical power, given the sample size and the effect size of the samples, which corresponds to the probability of identifying a real effect in the differences if there is one. For such, the effect size of the independent samples collected by the devices was determined using Cohen’s d measure, and the pooled standard deviation was used to measure the spread between the two groups of collected data. The effect size of the independent samples of equal size is a measure of the magnitude of the observed effect of the hypothesis being tested; in other words, it represents the practical importance difference in the parameter of interest, in this case, the mean of the differences. 

With regard to existence of potential outliers, as previously mentioned, these were detected as the points that were outside of the range of the LOA, and it is known that these types of points may compromise the results of the performed analysis. For instance, in this work, the measurements of body temperature, HR, and SpO_2_ collected with the e-CoVig were taken from the forehead, and the last two quantities were taken with a finger probe, from which we inferred that some of these measurements might have been affected by the occurrence of involuntary errors while taking the measurements and not due to issues with the device. After initial preprocessing of the data, we found an anomalous paired data point in SpO_2_, the pair (52,70)%, which is clinically an invalid value, and so it was omitted from the data. 

In all statistical tests, a two-sided hypothesis with a significance level of 5% (α = 0.05) was considered, as well as 95% confidence intervals. All numerical values are presented with two decimal places. The data were preprocessed and analyzed using Python.

## 3. Results

From the agreement analysis between the e-CoVig device and the standard clinically validated ones, we first present the summary statistics of the gathered data; then, for each measured quantity, we present the normality tests, the systemic error assessment, the Lin concordance correlation estimates, and, finally, the results of Bland–Altman analysis.

We evaluated the influence of the outlying points by calculating the elements of considered with the outliers removed and commenting on the differences obtained in the results. These data points were not excluded from the analysis, even if some of them could have been related to some type of error (non-random), as mentioned in the previous section. The outliers were determined by the data points that were outside the range of the limits of agreement in the Bland–Altman plots for each measured quantity.

### 3.1. Summary Statistics

The analysis comprised 86 participants, 48 men and 38 women, with an age distribution of 2 people aged 18–25 years old (y.o.), 14 aged 36–45 y.o., 12 aged 46–55 y.o., 16 aged 56–65 y.o., and 42 aged > 65 y.o., from which body temperature, heart rate, and oxygen saturation measurements were collected using both devices. In Table 1, we present the summary statistics of the measured quantities, where we include the mean, the standard deviation (sd), the minimum (min), the quartiles ($Q_{1}$ = 25%; $Q_{2}$ = 50%, which corresponds to the median; and $Q_{3}$ = 75%), and the maximum (max).

### 3.2. Normality Tests

Concerning the normality tests of the differences in the observed values for body temperature, we obtained a p-value of 0.36 for the K-S test and a p-value of 0.008 for the S-W test, which suggested the Normal distribution of the differences for the K-S test. As for the data with the outliers removed, we obtained p-values of 0.23 and 0.27 for the K-S and S-W tests, indicating that these points had a considerable influence on the distribution of the differences in the S-W test. In Figure A2a, we present the QQ plot of the differences for this variable, which shows a slight negative skewness as the data appear as a negative concave curve, showing an extended lower tail, a few outliers, and a reduced upper tail. This might explain the departure from the Normal distribution in the S-W test, since this test is more sensitive to deviations from the Normal distribution than the K-S test; nonetheless, the majority of points are approximately in line with the 45-degree line.

For heart rate, we obtained a p-value of 0.0003 for the K-S test, a p-value of << 0.05 for the S-W test, and p-values of 0.05 and of 0.003 on the K-S and S-W tests when the outliers were removed, from which we deduced that these points had a considerable influence on the deviance from normality. In Figure A4a, from the QQ plot of the HR differences, we can observe a flipped S shape formed by the points, presenting a positive excess kurtosis, where the lower and upper tails are extended. We can also observe that the majority of the points are not in line with the line of equality, although they are apparently symmetric; this might explain the non-normality of the result for both tests for the case with all data. 

For oxygen saturation, we obtained a p-value of 0.14 for the K-S test and a p-value of 0.0008 for the S-W test, which suggest the normality of the differences on the K-S test. For the data with the outliers removed, we obtained p-values of 0.17 and of 0.009 for the K-S and S-W tests, verifying the same results described previously and indicating that these data points had an influence on the deviation from normality. In Figure A6a, from the QQ plot of the SpO_2_ differences, we observe a positive concave curve, showing that the lower tail is reduced, and the upper tail is extended; a few outliers are present, similar to the QQ plot for body temperature but with the opposite concavity. This might explain the deviation from the Normal distribution in the S-W test; nonetheless, the majority of the points are approximately in line with the 45-degree line.

Hence, we confirmed the statistical significance of approximate normality of the differences in the body temperature and SpO_2_ measurements for the K-S test with all data points; when outliers are removed, for body temperature, HR, and SpO_2_, we verified the approximate normality in the differences in the K-S test with higher p-values, and the S-W test indicated the normality of the body temperature differences.

### 3.3. Systematic Errors and Power of Tests

Concerning the equality of the variance tests between the independent samples from the readings, for body temperature, we obtained p-values of 0.30, 0.27, and 0.45 when using the mean, median, and the trimmed mean respectively, in the estimation of the test statistics, which indicate the significance of the equality. Analyzing the data with the outliers removed, we obtained the same result but with higher p-values, indicating that the result was more significant when these data points were removed. For HR, the equality of the variance was statistically significant with p-values of 0.37, 0.36, and 0.38 when using the mean, the median and the trimmed mean, respectively. For the case without the outliers, we obtained higher p-values, indicating an equivalent result to the one obtained for body temperature. For SpO_2_, we obtained p-values of 0.05, 0.03, and 0.02 for the mean, median, and trimmed mean, respectively, meaning that only the equality of the variance for the mean was statistically significant. When the outliers were removed, we obtained p-values of 0.23, 0.14, and 0.09 for the mean, median, trimmed mean, respectively, indicating the significance of the equality of the variances between the samples.

In sequence, we present the results of the evaluation of the systematic additive bias in the difference in the means between the two measuring devices. For body temperature, the mean difference ${\bar{d}}_{Temp}$ = 0.6 °C, with standard deviation $s_{{\bar{d}}_{Temp}}$ = 0.74 °C. For both the paired t-test and Wilcoxon test, we obtained a p-value << 0.05, meaning that there was a systematic additive difference between the measuring devices. We see the QQ plot of the sample quantiles between the measurements from both devices in Figure A2b, showing a slight positive skewness, and the points on the tails depart from the main ones, which indicates deviation between the means. For heart rate, we had a mean difference ${\bar{d}}_{HR}$ = 1.23 BPM with $s_{{\bar{d}}_{HR}}$ = 9.85 BPM and a p-value of 0.61 for the paired t-test and 0.74 for the Wilcoxon test, indicating in this case that there was no additive bias. In Figure A4b, we present the sampled quantiles of the standard measurements against the e-CoVig measurements, where we can observe very few points in the tails that are not in line with the majority of the points. In turn, the vast majority of the points are approximately in line with the line of equality; this is thus a visual confirmation of the lack of additive bias in heart rate. For SpO_2_, we had ${\bar{d}}_{SpO2}$ = 2% with $s_{{\bar{d}}_{SpO2}}$ = 3.70% and a p-value < 0.05 for the paired Welch t-test and Wilcoxon test, indicating the existence of additive bias. In Figure A6b, showing the QQ plot of the sample quantiles for oxygen saturation_,_ we can observe a slightly positive skew pattern of the points, constituting a graphical confirmation of the presence of bias in this measured quantity. Thus, we inferred that there was a statistically significant additive bias in the mean difference in the body temperature and SpO_2_ measurements but no evidence of this in the heart rate measurements.

**Figure A1:**
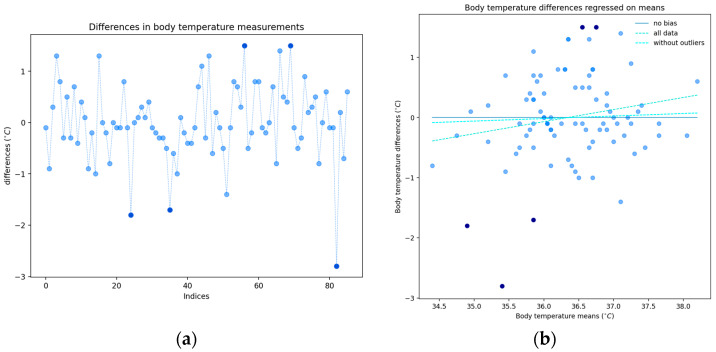
(**a**) Plot of difference in body temperature measurements between the standard clinically validated and e-CoVig devices; (**b**) plot of regression of the differences in the means of the measurements. The darker points correspond to the outliers determined by the Bland-Altman LOA, in both plots.

**Figure A2:**
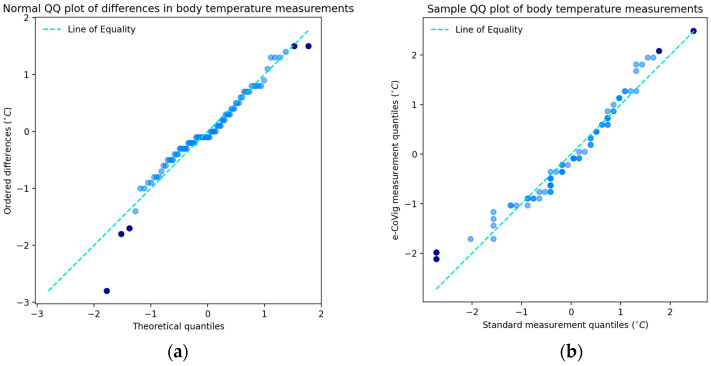
QQ plots of body temperature, where the darker points correspond to the outliers determined by the Bland-Altman LOA: (**a**) Normal quantiles against difference in measurements quantiles; (**b**) standard Normal sample quantiles against e-CoVig Normal sample quantiles.

**Figure A3:**
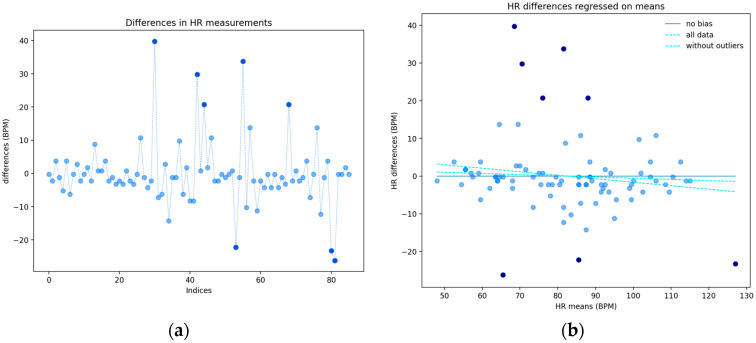
(**a**) Plot of difference in heart rate measurements between the standard clinically validated and e-CoVig devices; (**b**) plot of regression of the differences in the means of the measurements. The darker points correspond to the outliers determined by the Bland-Altaman LOA, in both plots.

**Figure A4:**
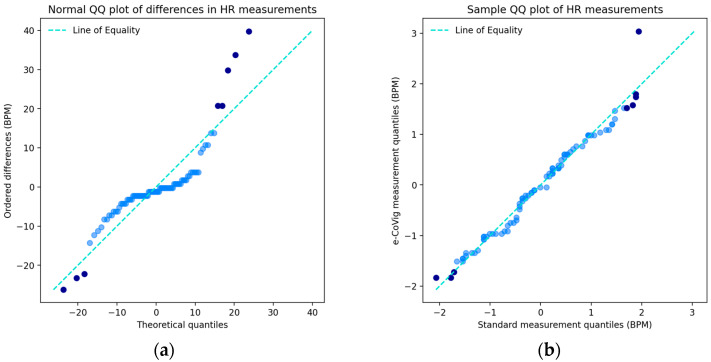
QQ plots of heart rate, where the darker points correspond to the outliers determined by the Bland-Altman LOA: (**a**) Normal quantiles against difference in measurements quantiles; (**b**) standard Normal sample quantiles against e-CoVig Normal sample quantiles.

**Figure A5:**
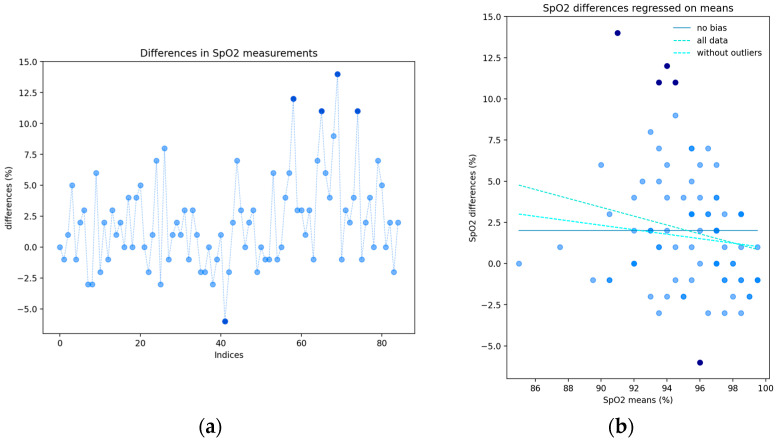
(**a**) Plot of differences of oxygen saturation measurements between the standard clinically validated and e-CoVig devices; (**b**) plot of regression of the differences in the means of the measurements. The darker points correspond to the outliers determined by the Bland-Altman LOA, in both plots.

**Figure A6:**
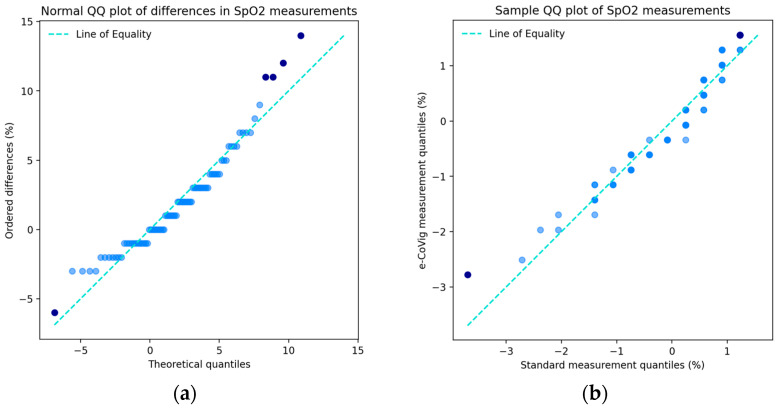
QQ plots of oxygen saturation, where the darker points correspond to the outliers determined by the Bland-Altman LOA: (**a**) Normal quantiles against difference in measurements quantiles; (**b**) standard Normal sample quantiles against e-CoVig Normal sample quantiles.

Regarding the proportional bias, after correcting the additive bias in the e-CoVig readings of the physiological quantities, we performed linear regressions between the differences and means of the measurements. In Figure A1b, we present the regression lines for body temperature, where we obtained $R^{2}$ = 0.04 for all data, meaning that about 4% of the variability in the differences was explained by the range of measurements, and $R^{2}$ = 0.003 for the data with the outliers removed, indicating that the multiplicative shift in this variable was not very significant and that the outliers were considerably influential. In Figure A3b, the same plot is presented for the heart rate differences and means, where $R^{2}$ = 0.03 for all data points, and $R^{2}$ = 0.01 for the data without the outliers, where the (multiplicative) shift is also not very significant. As for oxygen saturation, the regression plot can be seen in Figure A5b, with $R^{2}$ = 0.04 for all data points and $R^{2}$ = 0.02 for the case without the outliers. Again, we observe a small multiplicative bias. In these two quantities, we observed a decrease of 2% in the explained variance of the differences in the means. These results indicate that the relationship between the differences and the means of the measurements, across the range of measurements, was weak, and that the outliers contributed to the deviation of the regression line between the differences and the means from the zero line of a lack of proportional bias. Therefore, having found little influence of the range of measurements on the differences, the validity of the Bland–Altman plots was sufficiently assured [11].

The power of the tests of additive bias is now presented. For body temperature, the statistical power was 99.8% for an effect size of 0.74 for all data points, suggesting that the identification of the bias was conclusive, and the effect of the existing bias was easy to identify. The statistical power was 99.9% for an effect size of 0.83 when the outliers were removed. For SpO_2_, we obtained a power of 96.9% for an effect size of 0.59, and a power of 87.4% for an effect size of 0.49 for the data without outliers, so the same conclusion applied to this result, despite the fact that, in this parameter, the effects were weaker but still admissible for the identification of the effect. For heart rate, because the effect size was very small, 0.07 for all data points and 0.02 when outliers were removed, the effect of the differences of measurements collected from both devices could not be identified, suggesting that the differences were not important. Although we had a relatively small sample size, the power of the tests, as we showed, indicated that the sample size was adequate for the performed tests.

### 3.4. Body Temperature Agreement Analysis

**Figure 2:**
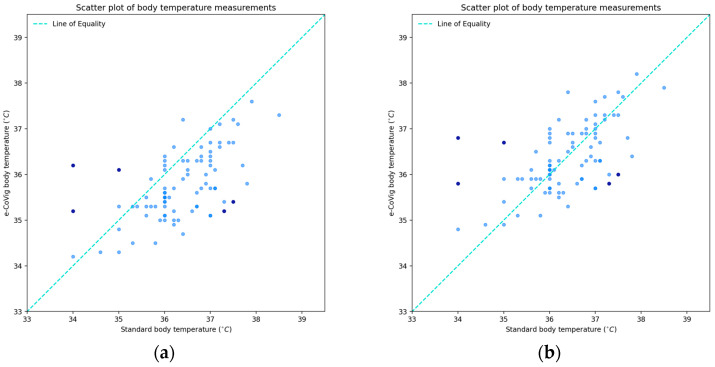
Scatter plots of body temperature measurements from standard clinically validated and e-CoVig devices. The darker points are the outliers determined by Bland–Altman LOA. (**a**) Plot with the raw measurements; (**b**) plot with the corrected bias.

Figure 2 shows the paired data measurements of body temperature from the standard device on the horizontal axis and from the novel e-CoVig device on the vertical axis. In the figure on the left, note the systematic additive shift, 0.6 ± 0.74 °C, and on the right, we present the rectified version, which was created by adding the additive bias for its correction; that is, the corrected e-CoVig reading equals the e-CoVig reading plus the mean of the differences between the clinically validated readings and e-CoVig readings.

For the raw data measurements, we had an estimated Lin’s correlation $\rho_{C}$ = 0.46, with a confidence interval of [0.32, 0.56], and for the data with corrected bias, we had $\rho_{C}$ = 0.59, with a confidence interval of [0.43, 0.71], which is a significant increase of 13% in the estimate of concordance, since, in this case, the data points were more in line with the line of equality. When the outliers were removed, we obtained $\rho_{C}$ = 0.71 in the additive correction case, which is an increase of 12% compared to the case with all data points, which is not surprising since the outlying points were removed from the regression in relation to the line of equality, indicating a noticeable influence of this correlation.

**Figure 3:**
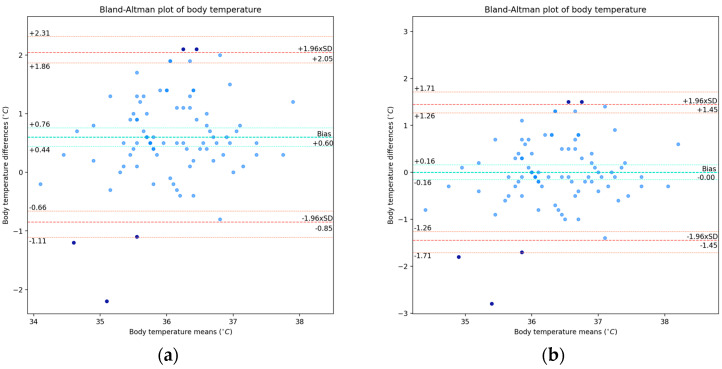
Bland–Altman plots of body temperature measurements. The darker points correspond to the outliers determined by the LOA: (**a**) plot with the raw differences and means; (**b**) plot with corrected bias.

We next present the Bland–Altman plots with exact confidence intervals. In Figure 3a, we present the results of the raw measurements, i.e., without the bias correction, and, in Figure 3b, we present the results for the rectified version of the data, i.e., with the bias corrected, which is equal to 0.6 °C.

For the corrected additive bias case, we obtained a confidence interval of [−0.16, 0.16] °C, and lower and upper LOAs of −1.45 and 1.45 °C, respectively, with exact confidence intervals of [−1.71, −1.26] and [1.26, 1.71] °C, respectively. Removing the outliers, we observed a slight increase in the fixed bias, given the removal of the three points below the lower LOA, but with a lesser deviation from it, 0.64 ± 0.59 °C, and the lower and upper LOAs were ±1.15 °C, with the span between the LOA being reduced from 2.9 °C to 2.3 °C, a reduction of 0.6 °C, which is somewhat significant.

Concerning the acceptance limits, we found that 61.6% of the points were within the clinically admissible margin, with the differences being ±0.5 °C and including 65.4% of the points of the data without the outliers. As for differences of ±1 °C, we found 87.2% and 92.6% of the points belonging to it for the cases with all data and with the outliers removed, respectively. 

With about 95% of the total points being within the limits of agreement and having found no significant relationship between the differences and the range of measurements, we verified the validity of the Bland–Altman plots and obtained fair concordance among the estimates, being almost moderate for the case with the bias corrected.

### 3.5. Heart Rate Agreement Analysis

**Figure 4:**
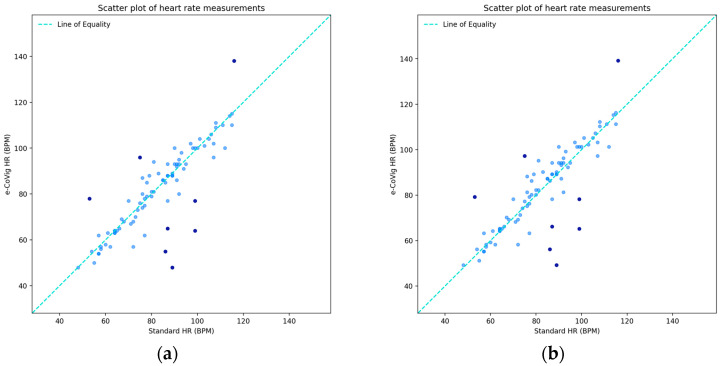
Scatter plots of heart rate measurements from standard clinically validated and e-CoVig devices. The darker points are the outliers determined by Bland–Altman LOA: (**a**) plot with the raw measurements; (**b**) plot with corrected additive shift (not considered as systematic bias).

For this physiological parameter, despite the absence of a systematic additive shift from the statistical point of view, we still considered its value correction by adding the additive shift to the e-CoVig readings in order to numerically adjust the values obtained with the new equipment, with the shift being 1.23 ± 9.85 BPM. In Figure 4, we present the paired data for the heart rate measurements, with the outliers depicted in the plots.

We obtained a concordance estimate of $\rho_{C}$ = 0.85, with a confidence interval of [0.77, 0.90] BPM and no significant differences between the coefficients and confidence intervals in both uncorrected and corrected additive shift cases, which was somehow expected as there was no statistically significant bias in these paired data. If the outliers were removed, we then had a remarkable $\rho_{C}$ = 0.96, revealing that all other points were much in line with the line of equality when the additive correction was applied.

**Figure 5:**
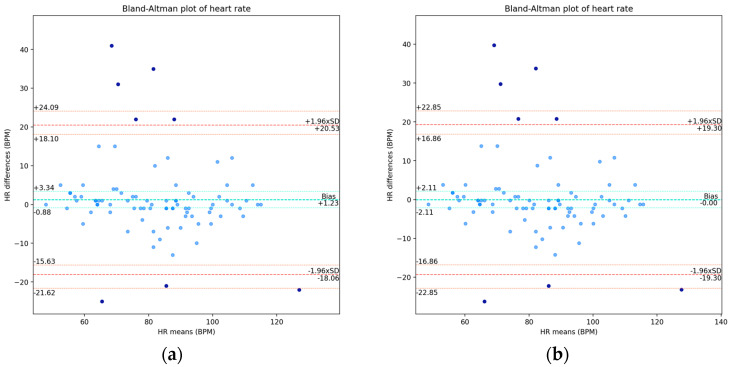
Bland–Altman plots of heart rate measurements. The darker points correspond to the outliers determined by the LOA: (**a**) plot with the raw differences and means; (**b**) plot with the corrected additive shift (not considered as systematic bias).

For this variable, we did not observe approximate normality in the differences’ distribution and, in particular, we found about 91% of the data within the LOA. The Bland–Altman plots are presented in Figure 5, for which we present the values of the LOA and the respective exact confidence intervals: for the raw data in Figure 5a, and for the rectified version in Figure 5b.

For the corrected mean shift, we obtained a confidence interval of [−2.11, 2.11] BPM, a lower LOA of −19.30 BPM, and an upper LOA of 19.30 BPM, with exact confidence intervals of [−22.86, −16.86] BPM and [16.86, 22.86] BPM, respectively. After discarding the outliers, the additive shift was 0.3 ± 5.07 BPM, which decreased with the removal of the most outlying points above the upper LOA, as well as decreased the deviation from it, as the outlying points ceased to influence it. The lower and upper LOAs became ±9.93 BPM, with the span between the LOA being reduced from 38.6 BPM to 19.86 BPM, a considerable reduction of 18.74 BPM in the range.

With regard to the acceptable limits in this physiological parameter, we found 69.8% between the clinically allowable range of ±5 BPM and 76.9% of the points within it when with the outliers removed. For the acceptable limits defined by the ±8 BPM margin, we found 76.7% of the points within it for the all-data case and 84.6% of the points within it in the case without the outliers.

Despite the assumption of 95% of the data points being between the limits of agreement not being established, the difference was 4%, three data points, and so this was not very relevant in quantitative terms. This lack of approximation to a Normal distribution of the differences requires further treatment of the data. Nonetheless, we found very good results of agreement between the measurements from both devices and no significant evidence of proportional bias, which indicate the agreement validity for this parameter.

### 3.6. Peripheral Oxygen Saturation Agreement Analysis

For oxygen saturation, we found a statistically significant additive bias in the difference in the means, 2 ± 3.7%, but it was not possible to correct it as there were a couple of measurements with 100% readings from both devices.

**Figure 6:**
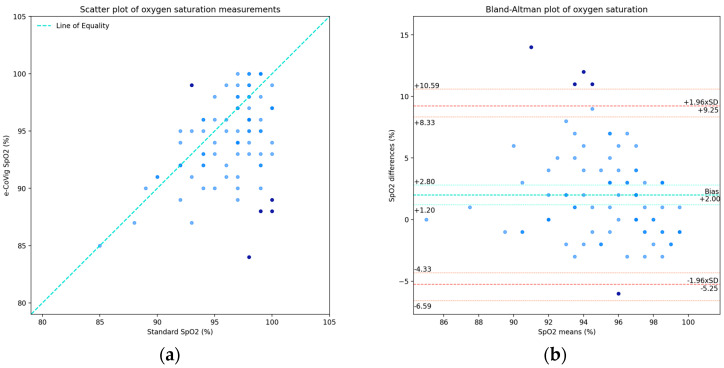
Oxygen saturation plots, where the darker points are the outliers determined by the LOA: (**a**) scatter plot of SpO_2_ measurements from standard clinically validated and e-CoVig devices; (**b**) Bland–Altman plot with the raw differences and means.

We obtained a concordance estimate of $\rho_{C}$ = 0.35, with confidence interval [0.18, 0.50] %. This slightly fair concordance correlation was in part due to the non-correction of the additive bias and possibly to the presence of outlying pairs. After removing the outliers, we obtained $\rho_{C}$ = 0.53, a considerable increase of 18% from the former, as the outlying points below the line of equality in Figure 6a deviated from the line of best fit to the points.

In Figure 6a, we present the scatter plot with the measurements from the e-CoVig device on the vertical axis and with the measurements from the standard device on the horizontal axis, where we show evidence of the additive bias in the paired data.

The Bland–Altman plot is presented in Figure 6b, which shows a “funnel effect” [16] (typical for SpO_2_ plots of means against differences), in which the variation in the differences is larger for smaller mean values and decreases as the mean values become larger. However, as previously mentioned, we found little expression of the effect size of the range of the measurements on the differences; though, if further corrected, it might improve the agreement between the readings of the two devices. The bias was 2% with a confidence interval of [1.20, 2.80]%; the lower LOA was −5.25% with an exact confidence of interval [−6.58, −4.33]%; and the upper LOA was 9.25%, with an exact confidence interval of [8.33, 10.58]%. After removing the outliers, we observed a bias of 1.6 ± 2.9%, a decrease with respect to the case with all points; the lower and upper LOAs became −4.09% and 7.29%, respectively, with the span between the LOAs being reduced from 14.5%to 11.38%, a reduction of about 3.12%.

As for the acceptable limits for SpO_2_, we found that 82.4% of the data were between the clinically allowable range of ±4% for the whole dataset and 87.5% within it when removing the outliers. For the acceptable range defined by the ±6% margin, we found 92.9% and 98.8% of the points within this margin for the case with all data points and for the case without the outliers, respectively.

Since 95% of the data points were within the limits of agreement, we considered the Bland–Altman agreement analysis as valid; however, from a statistical point of view, given the apparent dependency in the variation in the differences with respect to the means, even though minor, further analysis is required.

## 4. Discussion

We assessed the agreement between the clinically validated devices and the novel e-CoVig device for measuring selected physiological parameters, body temperature, heart rate, and oxygen saturation, in patients admitted to a respiratory intensive care unit. Using a combination of statistical methods, we analyzed the measurement errors between the devices, from which we were able to validate the Bland–Altman plots and obtain a more adequate measure of the agreement correlation between the single paired data measurements using Lin’s correlation coefficient.

The results obtained for body temperature are in line with those obtained by Cutuli S. L. et al. [17]. The authors performed a prospective experimental study in critically ill patients in an ICU, comparing invasive with noninvasive methods for measuring body temperature, where they obtained a bias of 0.66 °C and lower and upper limits of agreement of −1.23 and 2.55 °C, respectively, through forehead measurements. Compared with the work presented in [18] by Dolibog P. et al., our results are less concordant; the authors obtained a bias of −0.2 ± 0.58 °C and lower and upper LOAs of −1.35 and 0.95 °C, respectively, with the temperature being measured on the forehead with the tested device and in the tympanum with the reference device.

Our results for heart rate are similar to the ones obtained in [19], in which Downey C., et al. conducted an observational study on patients who had undergone major general surgery, obtaining a bias of −1.85 BPM and lower and upper LOAs equal to −23.92 and 20.22 BPM, respectively, from measurements taken from a patch on the chest of the patient with the testing device with wireless transmission and taken manually with the reference device. The results we obtained are also in agreement with the ones in [20]; Jacobs F. et al. conducted an observational study in patients recovering in a care unit after bariatric surgery, where they obtained a bias of −0.8 BPM and lower and upper limits of agreement of −19.3 and 17.8 BPM, respectively, where the measurements were also taken from a patch on the patient’s chest. These results pertain to patients presumably in sinus rhythm, whereas the validation of the e-CoVig device as an arrhythmia detector requires a different patient population with significant prevalence of atrial and ventricular arrhythmia as well as bradycardia.

The SpO_2_ results are somewhat in agreement with the those of Wilson B.J., et al. [21], where the authors performed a retrospective analysis of patients in an ICU with severe sepsis, having obtained a bias of 2.75% and limits of agreement of −3.4 and 8.9%, with measurements taken through a finger probe. Our results are less in agreement with those obtained by Thijssen M., et al. [22], where the authors performed a retrospective study in patients admitted to an ICU taking supplemental oxygen therapy, where they obtained a bias of 0.21 ± 2.04% and limits of agreement of −5.75 and 6.97%, and where the measurements were also collected using a finger probe.

From the comparison of our results with those of the abovementioned previous works, we found differences in the results, from which we confirmed that the process of measuring physiological parameters is uncertain and demanding, which necessitates the continued development, analysis, and testing of new low-cost and easy-to-use devices. These findings should encourage the research community to improve the precision of readings of these new mHealth device technologies, in particular in ICU environments, given the high standards for the monitoring of vital signs in critically ill patients.

The strengths of the present study include the e-CoVig testing in patients with acute severe respiratory diseases, for whom vital signs such as heart rate and pulse oximetry are frequently and significantly deviated from the normal range, increasing the confidence in the device’s performance in this specific population. In addition, measurements were taken in patients with different severity levels within the ICU: patients with acute respiratory failure on mechanical ventilation, patients in septic shock on vasopressor support, and less severe cases with people on noninvasive ventilatory support or on supplemental oxygen only. Finally, e-CoVig is a very user-friendly device, and its use does not require complex procedures, making it a potentially valuable tool for vital sign monitoring given its easy implementation process.

A unique aspect of this work, compared to many other similar studies, is the fact that we used exact confidence intervals for the limits of agreement, while some of the other studies in the literature did not make use of confidence intervals for the limits of agreement, or, when used, in some cases the application of the classical Bland–Altman plot was made in situations when the validation assumptions were not met.

From our retrospective reflection, some limitations were identified. Measurements were collected and statistical analysis was performed without taking into account potential confounders, such as patient medication at the time of measurement. This might be particularly relevant for drugs that interfere with peripheral perfusion, namely, vasoactive agents. In addition, although patients presenting arrhythmias at the time of the measurements were not excluded, the analysis was not stratified according to this factor; creating subgroups would be problematic because of the resulting small sample sizes. Other important issues to take into account would be device validation across different ethnicities and age groups. These are potential aspects to explore in future investigations using the e-CoVig device.

In relation to future work, certain aspects need further analysis in the statistical framework: (1) The deviation of the differences from the Normal distribution, even if slight, needs further attention. (2) The proportional bias, even being weak, needs further treatment. These aspects can be more thoroughly studied considering, for instance, (1) Transformation techniques, such as logarithmic and ratio transformations of the differences against the geometric mean and arithmetic mean of the means of the measurements. The percentage differences with the logarithmic mean or arithmetic mean of the means can be taken as denominators against the geometric and arithmetic means, respectively, having as reference, for example, [23,24,25]. These techniques can help mitigate some of the deviation from normality, as well as reduce some of the unwanted variability in the data. (2) A regression-based analysis of the fit to the differences can be used, with the inclusion of regressed horizontal limits around the line of best fit to the differences. This analysis can be performed, for instance, based on the works in [24,25], which can help in the estimation of the proportional bias and estimate possible trends in the data, enabling a formulation for their correction over the readings from the device. (3) Tolerance limits and prediction intervals around the line of best fit to the differences can be used, which may be more accurate in determining the adequacy of the closeness or equality between the two measuring methods in cases where any of the assumptions of the classical Bland–Altman plot are not fulfilled. The inclusion of these elements for future work can use the studies in [26,27,28] as a guide. By reanalyzing the data with the combination of the mentioned techniques, more rigorous and robust analyses of the collected data can be performed, and some of the outlying points can be better assessed.

We can state from this study that we were able to improve the state of the art by investigating the accuracy of the e-CoVig out-of-the-lab mHealth device in an ICU, which demonstrated a mean overall agreement with the standard clinically validated devices in the analyzed physiological parameters, despite the limitations previously mentioned. We also found considerable acceptance from the participants in this study regarding the use of the novel equipment. Nonetheless, further assessment of the errors is required with more general methods, taking into account methods for non-normally distributed differences. We must also consider adjustment methods for the proportional bias, even though weak, as well as a more thorough analysis of the outliers closest to the limits of agreement, and we could consider a prospective observational study with repeated within-subject measurements. We must add that we are working on implementing a healthcare decision support system and that this method is not intended to replace any medical staff or pre-established routines.

## Figures and Tables

**Table 1:** Summary statistics of the measured physiological variables for the standard and e-CoVig devices.

Variable	Mean	SD	Min	25%	50%	75%	Max
Standard Temp (°C)	36.36	0.87	34	36	36.4	37	38.5
e-CoVig Temp (°C)	35.76	0.74	34.2	35.3	35.7	36.3	37.6
Standard HR (BPM)	83.1	17.05	48	71.25	85	93.75	116
e-CoVig HR (BPM)	81.87	18.59	48	65	83	94.75	138
Standard SpO_2_ (%)	96.23	3.06	85	94	97	98	100
e-CoVig SpO_2_ (%)	94.26	3.71	84	92	95	97	100

## Data Availability

Data are contained within this article.

## Supplementary Materials

The following supporting information can be downloaded at: https://www.mdpi.com/article/10.3390/s24165164/s1.

## Appendix A

In all plots presented below, the additive bias was rectified, except in the case of oxygen saturation (SpO_2_), and the darker points in the plots correspond to the outliers determined by the respective Bland–Altman plot.
