# Supplementary material for: Validation of an mHealth System for Monitoring Fundamental Physiological Parameters in the Clinical Setting
Source: Sensors (Basel). 2024 Aug 10;24(16):5164. doi: 10.3390/s24165164 (PMC11359666; doi:10.3390/s24165164)
Supplement: Supplementary file 1 [file sensors-24-05164-s001.zip › max30101.pdf]

MAX30101

High-Sensitivity Pulse Oximeter and Heart-Rate Sensor for Wearable Health

General Description

The MAX30101 is an integrated pulse oximetry and heart-rate monitor module. It includes internal LEDs, photodetectors, optical elements, and low-noise electronics with ambient light rejection. The MAX30101 provides a complete system solution to ease the design-in process for mobile and wearable devices.

The MAX30101 operates on a single 1.8V power supply and a separate 5.0V power supply for the internal LEDs. Communication is through a standard I^2C-compatible interface. The module can be shut down through software with zero standby current, allowing the power rails to remain powered at all times.

Applications

- Wearable Devices
- Fitness Assistant Devices
- Smartphones
- Tablets

Benefits and Features

- Heart-Rate Monitor and Pulse Oximeter Sensor in LED Reflective Solution
- Tiny 5.6mm x 3.3mm x 1.55mm 14-Pin Optical Module
  - Integrated Cover Glass for Optimal, Robust Performance
- Ultra-Low-Power Operation for Mobile Devices
  - Programmable Sample Rate and LED Current for Power Savings
  - Low-Power Heart-Rate Monitor (< 1mW)
  - Ultra-Low Shutdown Current (0.7µA, typ)
- Fast Data Output Capability
  - High Sample Rates
- Robust Motion Artifact Resilience
  - High SNR
- -40°C to +85°C Operating Temperature Range

***Ordering Information** appears at end of data sheet.*

System Diagram

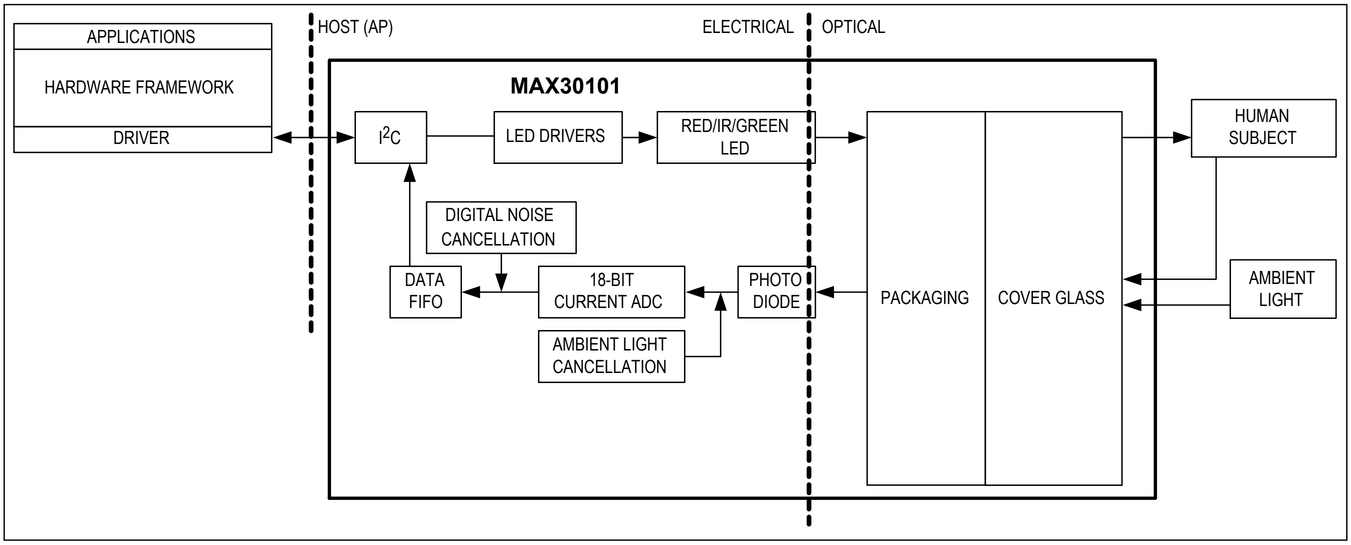

Absolute Maximum Ratings

V_{DD} to GND .... -0.3V to +2.2V
GND to PGND .... -0.3V to +0.3V
V_{LED}+ to PGND .... -0.3V to +6.0V
All Other Pins to GND .... -0.3V to +6.0V
Output Short-Circuit Current Duration .... Continuous
Continuous Input Current into Any Terminal .... ±20mA

Continuous Power Dissipation (T_A = +70°C) OESIP (derate 5.5mW/°C above +70°C) 440mW
Operating Temperature Range .... -40°C to +85°C
Junction Temperature .... +90°C
Soldering Temperature (reflow) .... +260ºC
Storage Temperature Range .... -40ºC to +105ºC

Stresses beyond those listed under "Absolute Maximum Ratings" may cause permanent damage to the device. These are stress ratings only, and functional operation of the device at these or any other conditions beyond those indicated in the operational sections of the specifications is not implied. Exposure to absolute maximum rating conditions for extended periods may affect device reliability.

Package Information

14 OESIP

Package Code	F143A5+1
Outline Number	21-1048
Land Pattern Number	90-0602
**THERMAL RESISTANCE, FOUR-LAYER BOARD**	
Junction-to-Ambient ($θ_{JA}$)	180°C/W
Junction-to-Case Thermal Resistance ($θ_{JC}$)	150°C/W

For the latest package outline information and land patterns (footprints), go to www.maximintegrated.com/packages. Note that a "+", "#", or "-" in the package code indicates RoHS status only. Package drawings may show a different suffix character, but the drawing pertains to the package regardless of RoHS status.

Package thermal resistances were obtained using the method described in JEDEC specification JESD51-7, using a four-layer board. For detailed information on package thermal considerations, refer to www.maximintegrated.com/thermal-tutorial.

Electrical Characteristics

(V_{DD} = 1.8V, V_{LED}+ = 5.0V, T_A = +25°C, min/max are from T_A = -40°C to +85°C, unless otherwise noted. Typical values are at T_A = 25°C.) (Note 1)

PARAMETER	SYMBOL	CONDITIONS	MIN	TYP	MAX	UNITS
**POWER SUPPLY**						
Power-Supply Voltage	V_{DD}	Guaranteed by RED and IR count tolerance	1.7	1.8	2.0	V
LED Supply Voltage VLED+ to PGND	V_{LED+}	Guaranteed by PSRR of LED driver (RED and IR LED only)	3.1	3.3	5.0	V
		Guaranteed by PSRR of LED driver (GREEN LED only). T_A = 25°C	4.5	5.0	5.5	
Supply Current	I_{DD}	SpO_2 and HR mode, PW = 215μs, 50sps		600	1100	μA
		IR only mode, PW = 215μS, 50sps		600	1100	
Supply Current in Shutdown	I_{SHDN}	T_A = +25°C, MODE = 0x80		0.7	2.5	μA
**PULSE OXIMETRY/HEART-RATE SENSOR CHARACTERISTICS**						
ADC Resolution				18		bits

Electrical Characteristics (continued)

(V_{DD} = 1.8V, V_{LED+} = 5.0V, T_A = +25°C, min/max are from T_A = -40°C to +85°C, unless otherwise noted. Typical values are at T_A = 25°C.) (Note 1)

PARAMETER	SYMBOL	CONDITIONS		MIN	TYP	MAX	UNITS
Red ADC Count (Note 2)	REDC	LED1_PA = 0x0C, LED_PW = 0x01, SPO2_SR = 0x05, ADC_RGE = 0x00			65536		Counts
IR ADC Count (Note 2)	IRC	LED2_PA = 0x0C, LED_PW = 0x01, SPO2_SR = 0x05 ADC_RGE = 0x00			65536		Counts
Green ADC Count (Note 2)	GRNC	LED3_PA = LED4_PA = 0x24, LED_PW = 0x01, SPO2_SR = 0x05, ADC_RGE = 0x00			65536		Counts
Dark Current Count	LED_DCC	LED1_PA = LED2_PA = 0x00, LED_PW = 0x03, SPO2_SR = 0x01 ADC_RGE = 0x02			30	128	Counts
		LED1_PA = LED2_PA = 0x00, LED_PW = 0x03, SPO2_SR = 0x01 ADC_RGE = 0x03			0.01	0.05	% of FS
DC Ambient Light Rejection (Note 3)	ALR	ADC counts with finger on sensor under direct sunlight (100K lux), ADC_RGE = 0x3, LED_PW = 0x03, SPO2_SR = 0x01	Red LED		2		Counts
		ADC counts with finger on sensor under direct sunlight (100K lux), ADC_RGE = 0x3, LED_PW = 0x03, SPO2_SR = 0x02	IR LED		2		
ADC Count—PSRR (VDD)	$PSRRV_{DD}$	1.7V < VDD < 2.0V, LED_PW = 0x00, SPO2_SR = 0x05			0.25	1	% of FS
		Frequency = DC to 100kHz, $100mV_{P-P}$			10		LSB
ADC Count—PSRR (LED Driver Outputs)	$PSRR_{LED}$	3.1V < VLED+ < 5.0V, LED1_PA = LED2_PA = 0x0C, LED_PW = 0x01, SPO2_SR = 0x05			0.05	1	% of FS
		4.5V < VLED+ < 5.5V, T_A = 25°C LED3_PA = LED4_PA = 0x24, LED_PW = 0x01, SPO2_SR = 0x05			0.05	1	
		Frequency = DC to 100kHz, 100mVP-P			10		LSB
ADC Clock Frequency	CLK			10.2	10.48	10.8	MHz
ADC Integration Time (Note 3)	INT	LED_PW = 0x00			69		µs
		LED_PW = 0x01			118		
		LED_PW = 0x02			215		
		LED_PW = 0x03			411		

Electrical Characteristics (continued)

(V_{DD} = 1.8V, V_{LED}+ = 5.0V, T_A = +25°C, min/max are from T_A = -40°C to +85°C, unless otherwise noted. Typical values are at T_A = 25°C.) (Note 1)

PARAMETER	SYMBOL	CONDITIONS	MIN	TYP	MAX	UNITS
Slot Timing (Timing Between Sequential Channel Samples; e.g., Red Pulse Rising Edge To IR Pulse Rising Edge)	INT	LED_PW = 0x00		427		µs
		LED_PW = 0x01		525		
		LED_PW = 0x02		720		
		LED_PW = 0x03		1107		
**COVER GLASS CHARACTERISTICS (Note 3)**						
Hydrolytic Resistance Class		Per DIN ISO 719	HGB 1			
**IR LED CHARACTERISTICS (Note 3)**						
LED Peak Wavelength	λP	I_{LED} = 20mA, T_A = +25°C	870	880	900	nm
Full Width at Half Max	Δλ	I_{LED} = 20mA, T_A = +25°C		30		nm
Forward Voltage	VF	I_{LED} = 20mA, T_A = +25°C		1.4		V
Radiant Power	P_O	I_{LED} = 20mA, T_A = +25°C		6.5		mW
**RED LED CHARACTERISTICS (Note 3)**						
LED Peak Wavelength	λ_P	I_{LED} = 20mA, T_A = +25°C	650	660	670	nm
Full Width at Half Max	Δ_λ	I_{LED} = 20mA, T_A = +25°C		20		nm
Forward Voltage	V_F	I_{LED} = 20mA, T_A = +25°C		2.1		V
Radiant Power	P_O	I_{LED} = 20mA, T_A = +25°C		9.8		mW
**GREEN LED CHARACTERISTICS (Note 3)**						
LED Peak Wavelength	λ_P	I_{LED} = 50mA, T_A = +25°C	530	537	545	nm
Full Width at Half Max	Δ_λ	I_{LED} = 50mA, T_A = +25°C		35		nm
Forward Voltage	V_F	I_{LED} = 50mA, T_A = +25°C		3.3		V
Radiant Power	P_O	I_{LED} = 50mA, T_A = +25°C		17.2		mW
**PHOTODETECTOR CHARACTERISTICS (Note 3)**						
Spectral Range of Sensitivity	Λ > 30% QE	QE: Quantum Efficiency	640		980	nm
Radiant Sensitive Area	A			1.36		mm2
Dimensions of Radiant Sensitive Area	L x W			1.38 x 0.98		mm x mm
**INTERNAL DIE TEMPERATURE SENSOR**						
Temperature ADC Acquisition Time	T_T	T_A = +25°C		29		ms
Temperature Sensor Accuracy	T_A	T_A = +25°C		±1		°C
Temperature Sensor Minimum Range	T_{MIN}			-40		°C
Temperature Sensor Maximum Range	T_{MAX}			85		°C

Electrical Characteristics (continued)

(V_{DD} = 1.8V, V_{LED+} = 5.0V, T_A = +25°C, min/max are from T_A = -40°C to +85°C, unless otherwise noted. Typical values are at T_A = 25°C.) (Note 1)

PARAMETER	SYMBOL	CONDITIONS	MIN	TYP	MAX	UNITS
**DIGITAL INPUTS (SCL, SDA)**						
Input Logic-Low Voltage	V_{IL}				0.3 x V_{DD}	V
Input Logic-High Voltage	V_{IH}		0.7 x V_{DD}			V
Input Hysteresis	V_{HYS}			0.5 x V_{DD}		V
Input Leakage Current	I_{IN}			±0.1	±1	µA
Input Capacitance	C_{IN}			10		pF
**DIGITAL OUTPUTS (SDA, $\overline{INT}$)**						
Output Low Voltage	VOL	I_{SINK} = 3mA			0.4	V
**I^2C TIMING CHARACTERISTICS**						
I^2C Write Address				AE		Hex
I^2C Read Address				AF		Hex
SCL Clock Frequency	f_{SCL}	Lower limit not tested	0		400	kHz
Bus Free Time Between STOP and START Condition	t_{BUF}		1.3			µs
Hold Time (Repeated) START Condition	$t_{HD,STA}$		0.6			µs
SCL Pulse-Width Low	t_{LOW}		1.3			µs
SCL Pulse-Width High	t_{HIGH}		0.6			µs
Setup Time for a Repeated START Condition	$t_{SU,STA}$		0.6			µs
Data Hold Time	$t_{HD;DAT}$		0		0.9	µs
Data Setup Time	$t_{SU;DAT}$		100			ns
Setup Time for STOP Condition	$t_{SU;STO}$		0.6			µs
Pulse Width of Suppressed Spike	t_{SP}				50	ns
Bus Capacitance	C_b				400	pF
SDA and SCL Receiving Rise Time	T_r	(Note 4)	20		300	ns
SDA and SCL Receiving Fall Time	t_{Rf}	(Note 4)	20 x V_{DD}/5.5		300	ns
SDA Transmitting Fall Time	t_{of}		20 x V_{DD}/5.5		250	ns

Note 1: All devices are 100% production tested at T_A = +25°C. Specifications over temperature limits are guaranteed by Maxim Integrated's bench or proprietary automated test equipment (ATE) characterization.

Note 2: Specifications are guaranteed by Maxim Integrated's bench characterization and by 100% production test using proprietary ATE setup and conditions.

Note 3: For design guidance only. Not production tested.

Note 4: These specifications are guaranteed by design, characterization, or I²C protocol.

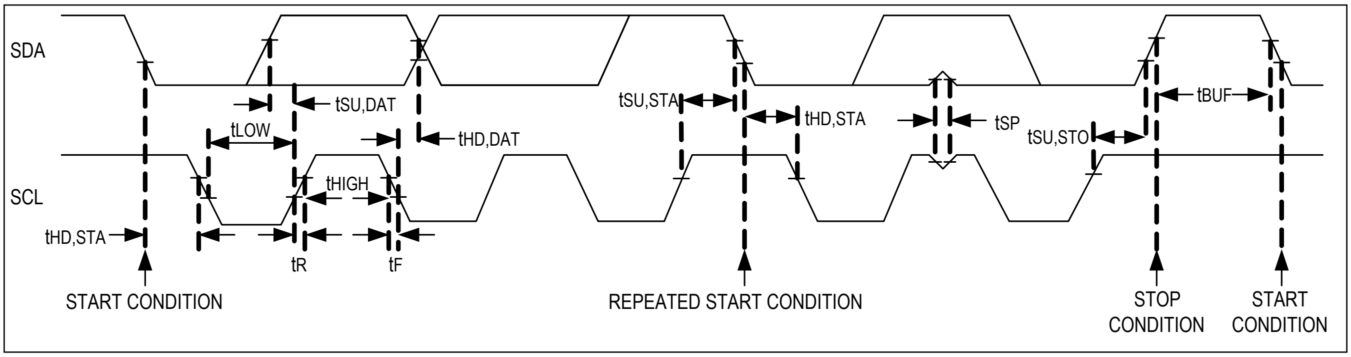

Figure 1. I²C-Compatible Interface Timing Diagram

Typical Operating Characteristics

(V_{DD} = 1.8V, V_{LED+} = 5.0V, T_A = +25°C, $\overline{RST}$, unless otherwise noted.)

RED LED SUPPLY HEADROOM

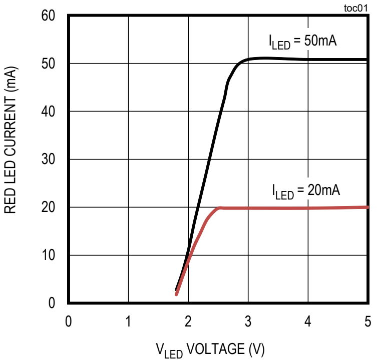

IR LED SUPPLY HEADROOM

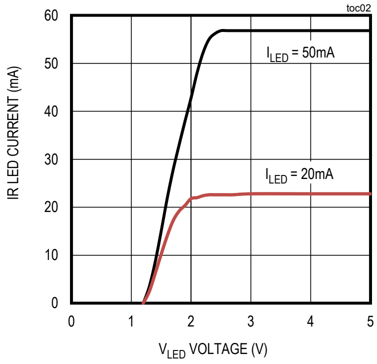

GREEN LED SUPPLY HEADROOM

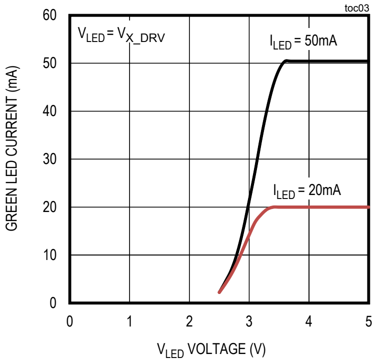

V_{DD} SUPPLY CURRENT vs. SUPPLY VOLTAGE

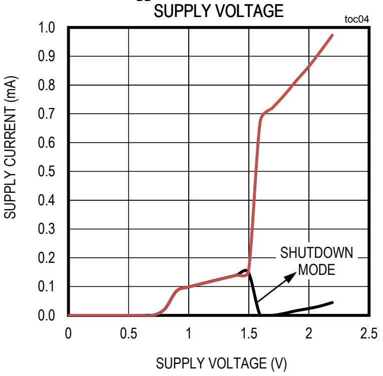

DC COUNTS vs. DISTANCE FOR WHITE HIGH IMPACT STYRENE CARD

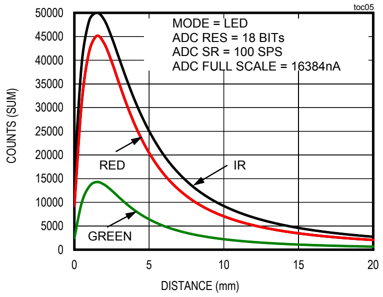

V_{DD} SHUTDOWN CURRENT vs. TEMPERATURE

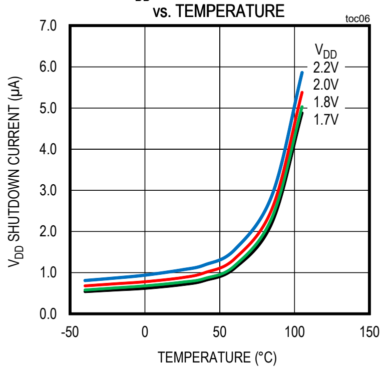

V_{LED} SHUTDOWN CURRENT vs. TEMPERATURE

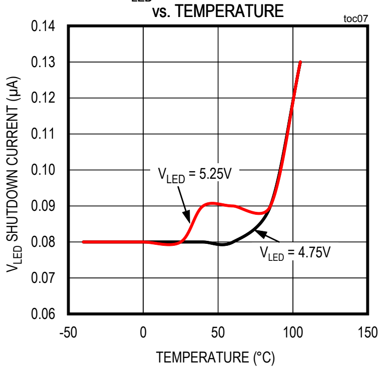

RED LED SPECTRA AT T_A = +30°C

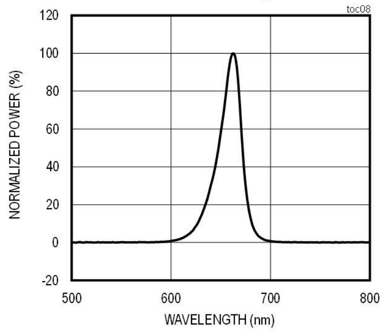

IR LED SPECTRA AT T_A = +30°C

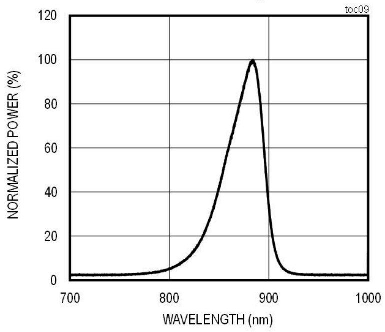

Typical Operating Characteristics (continued)

(V_{DD} = 1.8V, V_{LED+} = 5.0V, T_A = +25°C, $\overline{RST}$, unless otherwise noted.)

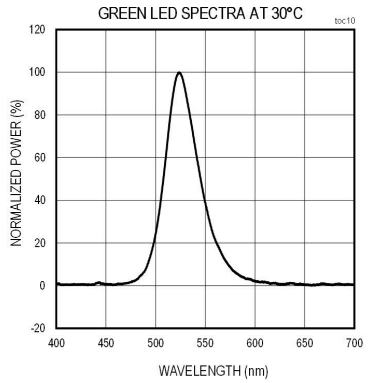

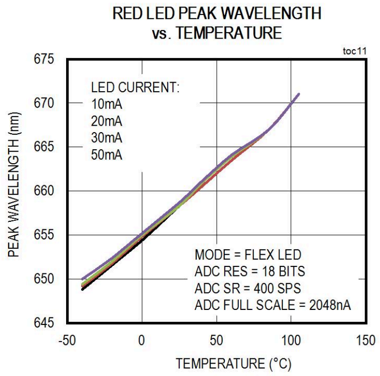

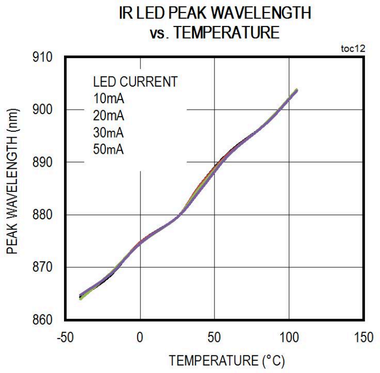

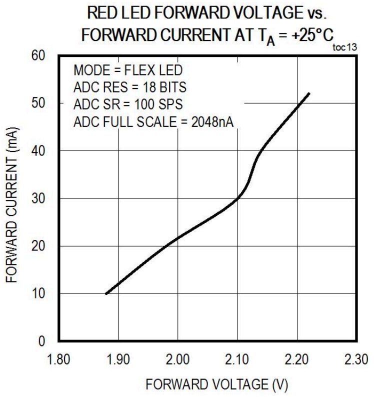

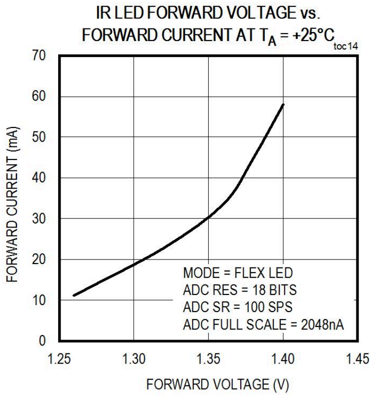

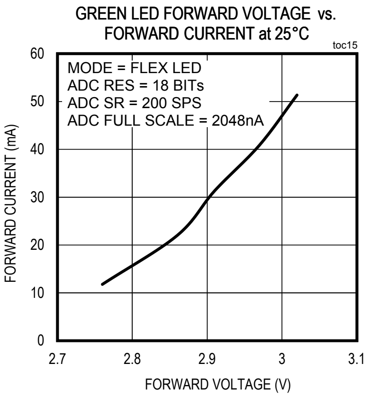

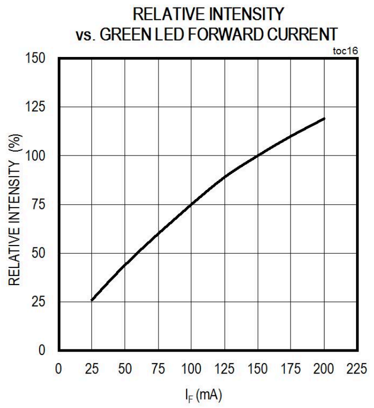

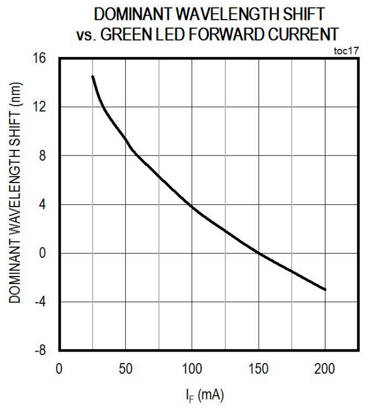

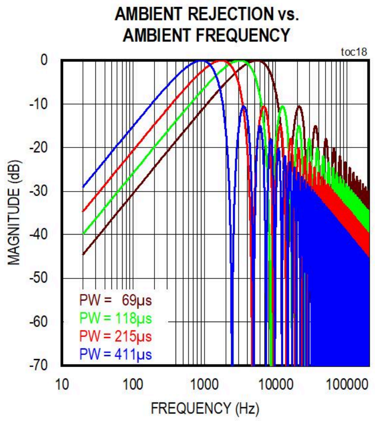

Typical Operating Characteristics (continued)

(V_{DD} = 1.8V, V_{LED+} = 5.0V, T_A = +25°C, $\overline{RST}$, unless otherwise noted.)

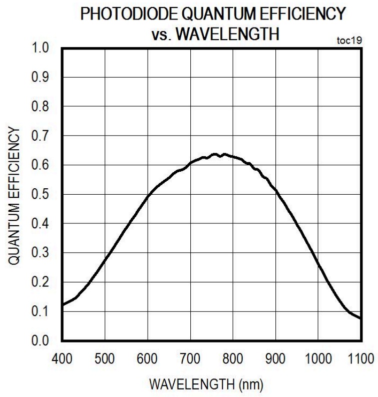

Pin Configuration

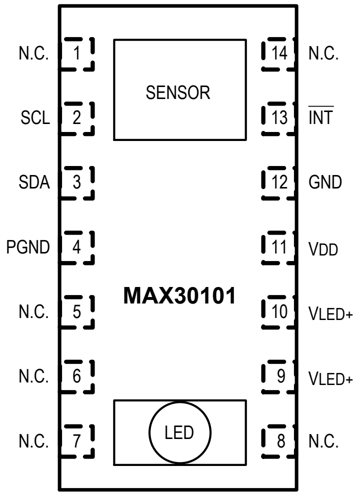

Pin Description

PIN	NAME	FUNCTION
1, 5, 6, 7, 8, 14	N.C.	No Connection. Connect to PCB pad for mechanical stability.
2	SCL	I^2C Clock Input
3	SDA	I^2C Clock Data, Bidirectional (Open-Drain)
4	PGND	Power Ground of the LED Driver Blocks
9, 10	V_{LED+}	LED Power Supply (anode connection). Use a bypass capacitor to PGND for best performance.
11	V_{DD}	Analog Power Supply Input. Use a bypass capacitor to GND for best performance.
12	GND	Analog Ground
13	$\overline{INT}$	Active-Low Interrupt (Open-Drain). Connect to an external voltage with a pullup resistor.

Functional Diagrams

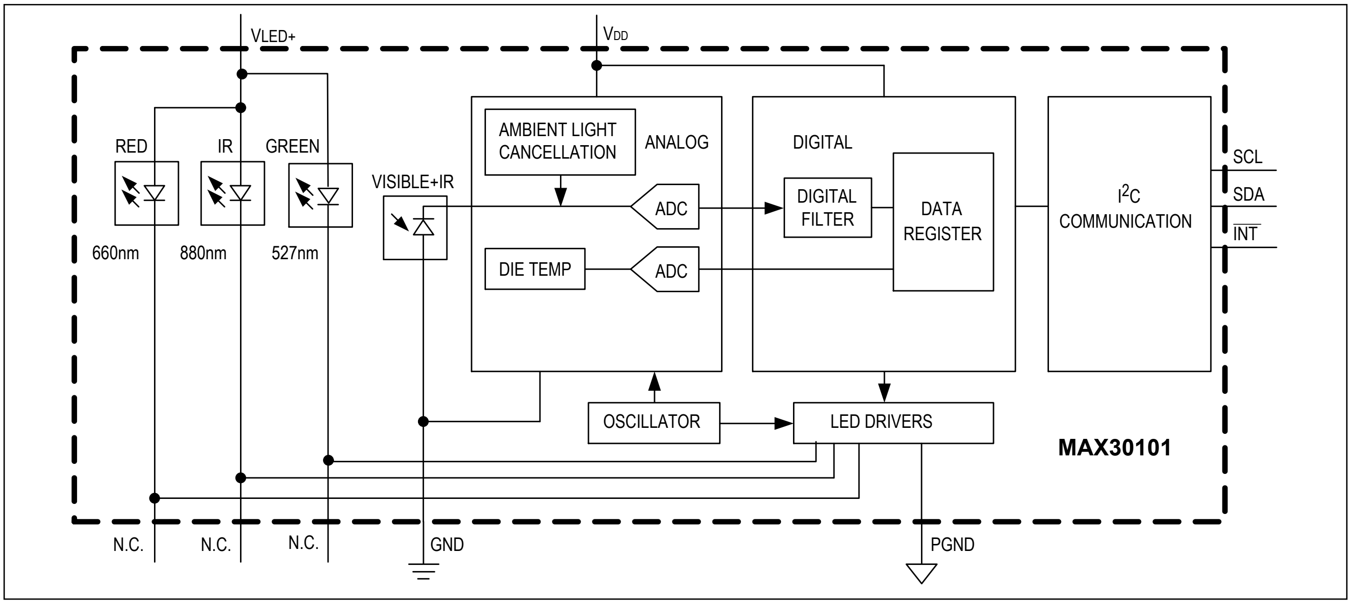

Detailed Description

The MAX30101 is a complete pulse oximetry and heart- rate sensor system solution module designed for the demanding requirements of wearable devices. The MAX30101 maintains a very small solution size without sacrificing optical or electrical performance. Minimal external hardware components are required for integra- tion into a wearable system.

The MAX30101 is fully adjustable through software regis- ters, and the digital output data can be stored in a 32-deep FIFO within the IC. The FIFO allows the MAX30101 to be connected to a microcontroller or processor on a shared bus, where the data is not being read continuously from the MAX30101's registers.

SpO_2 Subsystem

The SpO_2 subsystem contains ambient light cancellation (ALC), a continuous-time sigma-delta ADC, and propri- etary discrete time filter. The ALC has an internal Track/ Hold circuit to cancel ambient light and increase the effec- tive dynamic range. The SpO_2 ADC has a programmable full-scale ranges from 2μA to 16μA. The ALC can cancel up to 200μA of ambient current.

The internal ADC is a continuous time oversampling sigma-delta converter with 18-bit resolution. The ADC sampling rate is 10.24MHz. The ADC output data rate can be programmed from 50sps (samples per second) to 3200sps.

Temperature Sensor

The MAX30101 has an on-chip temperature sensor for calibrating the temperature dependence of the SpO_2 subsystem. The temperature sensor has an inherent resolution 0.0625°C.

The device output data is relatively insensitive to the wavelength of the IR LED, where the red LED's wave- length is critical to correct interpretation of the data. An SpO_2 algorithm used with the MAX30101 output signal can compensate for the associated SpO_2 error with ambient temperature changes.

LED Driver

The MAX30101 integrates red, green, and IR LED drivers to modulate LED pulses for SpO_2 and HR measurements. The LED current can be programmed from 0 to 50mA with proper supply voltage. The LED pulse width can be programmed from 69μs to 411μs to allow the algorithm to optimize SpO_2 and HR accuracy and power consumption

based on use cases.

Register Maps and Descriptions

REGISTER	B7	B6	B5	B4	B3	B2	B1	B0	REG ADDR	POR STATE	R/W
STATUS											
Interrupt Status 1	A_FULL	PPG_ RDY	ALC_ OVF					PWR_ RDY	0x00	0X00	R
Interrupt Status 2							DIE_TEMP _RDY		0x01	0x00	R
Interrupt Enable 1	A_FULL_ EN	PPG_ RDY_EN	ALC_ OVF_EN						0x02	0X00	R/W
Interrupt Enable 2							DIE_TEMP _RDY_EN		0x03	0x00	R/W
FIFO											
FIFO Write Pointer				FIFO_WR_PTR[4:0]					0x04	0x00	R/W
Overflow Counter				OVF_COUNTER[4:0]					0x05	0x00	R/W
FIFO Read Pointer				FIFO_RD_PTR[4:0]					0x06	0x00	R/W
FIFO Data Register	FIFO_DATA[7:0]								0x07	0x00	R/W
CONFIGURATION											
FIFO Configuration	SMP_AVE[2:0]			FIFO_ ROLL OVER_EN	FIFO_A_FULL[3:0]				0x08	0x00	R/W
Mode Configuration	SHDN	RESET				MODE[2:0]			0x09	0x00	R/W
SpO2 Configuration	0 (Reserved)	SPO2_ADC_RGE [1:0]		SPO2_SR[2:0]			LED_PW[1:0]		0x0A	0x00	R/W
RESERVED									0x0B	0x00	R/W
LED Pulse Amplitude	LED1_PA[7:0]								0x0C	0x00	R/W
	LED2_PA[7:0]								0x0D	0x00	R/W
	LED3_PA[7:0]								0x0E	0x00	R/W
	LED4_PA[7:0]								0x0F	0x00	R/W
Multi-LED Mode Control Registers		SLOT2[2:0]				SLOT1[2:0]			0x11	0x00	R/W
		SLOT4[2:0]				SLOT3[2:0]			0x12	0x00	R/W
RESERVED									0x13–0x17	0xFF	R/W
RESERVED									0x18-0x1E	0x00	R
DIE TEMPERATURE											

REGISTER	B7	B6	B5	B4	B3	B2	B1	B0	REG ADDR	POR STATE	R/W
Die Temp Integer	TINT[7:0]								0x1F	0x00	R
Die Temp Fraction					TFRAC[3:0]				0x20	0x00	R
Die Temperature Config								TEMP_EN	0x21	0x00	R/W
RESERVED									0x22–0x2F	0x00	R/W
PART ID											
Revision ID	REV_ID[7:0]								0xFE	0xXX*	R
Part ID	PART_ID[7]								0xFF	0x15	R

*XX denotes a 2-digit hexadecimal number (00 to FF) for part revision identification. Contact Maxim Integrated for the revision ID number assigned for your product.

Interrupt Status (0x00–0x01)

REGISTER	B7	B6	B5	B4	B3	B2	B1	B0	REG ADDR	POR STATE	R/W
Interrupt Status 1	A_FULL	PPG_RDY	ALC_OVF					PWR_RDY	0x00	0X00	R
Interrupt Status 2							DIE_TEMP_RDY		0x01	0x00	R

Whenever an interrupt is triggered, the MAX30101 pulls the active-low interrupt pin into its low state until the interrupt is cleared.

A_FULL: FIFO Almost Full Flag

In SpO_2 and HR modes, this interrupt triggers when the FIFO write pointer has a certain number of free spaces remaining. The trigger number can be set by the FIFO_A_FULL[3:0] register. The interrupt is cleared by reading the Interrupt Status 1 register (0x00).

PPG_RDY: New FIFO Data Ready

In SpO_2 and HR modes, this interrupt triggers when there is a new sample in the data FIFO. The interrupt is cleared by reading the Interrupt Status 1 register (0x00), or by reading the FIFO_DATA register.

ALC_OVF: Ambient Light Cancellation Overflow

This interrupt triggers when the ambient light cancellation function of the SpO_2/HR photodiode has reached its maximum limit, and therefore, ambient light is affecting the output of the ADC. The interrupt is cleared by reading the Interrupt Status 1 register (0x00).

PWR_RDY: Power Ready Flag

On power-up or after a brownout condition, when the supply voltage V_{DD} transitions from below the undervoltage lockout (UVLO) voltage to above the UVLO voltage, a power-ready interrupt is triggered to signal that the module is powered-up and ready to collect data.

DIE_TEMP_RDY: Internal Temperature Ready Flag

When an internal die temperature conversion is finished, this interrupt is triggered so the processor can read the temperature data registers. The interrupt is cleared by reading either the Interrupt Status 2 register (0x01) or the TFRAC register (0x20).

The interrupts are cleared whenever the interrupt status register is read, or when the register that triggered the interrupt is read. For example, if the SpO_2 sensor triggers an interrupt due to finishing a conversion, reading either the FIFO data register or the interrupt register clears the interrupt pin (which returns to its normal HIGH state). This also clears all the bits in the interrupt status register to zero.

Interrupt Enable (0x02-0x03)

REGISTER	B7	B6	B5	B4	B3	B2	B1	B0	REG ADDR	POR STATE	R/W
Interrupt Enable 1	A_ FULL_ EN	PPG_ RDY_EN	ALC_ OVF_EN						0x02	0X00	R/W
Interrupt Enable 2							DIE_TEMP_ RDY_EN		0x03	0x00	R/W

Each source of hardware interrupt, with the exception of power ready, can be disabled in a software register within the MAX30101 IC. The power-ready interrupt cannot be disabled because the digital state of the module is reset upon a brownout condition (low power supply voltage), and the default condition is that all the interrupts are disabled. Also, it is important for the system to know that a brownout condition has occurred, and the data within the module is reset as a result.

The unused bits should always be set to zero for normal operation.

FIFO (0x04–0x07)

REGISTER	B7	B6	B5	B4	B3	B2	B1	B0	REG ADDR	POR STATE	R/W
FIFO Write Pointer				FIFO_WR_PTR[4:0]					0x04	0x00	R/W
Over Flow Counter				OVF_COUNTER[4:0]					0x05	0x00	R/W
FIFO Read Pointer				FIFO_RD_PTR[4:0]					0x06	0x00	R/W
FIFO Data Register	FIFO_DATA[7:0]								0x07	0x00	R/W

FIFO Write Pointer

The FIFO Write Pointer points to the location where the MAX30101 writes the next sample. This pointer advances for each sample pushed on to the FIFO. It can also be changed through the I^2C interface when MODE[2:0] is 010, 011, or 111.

FIFO Overflow Counter

When the FIFO is full, samples are not pushed on to the FIFO, samples are lost. OVF_COUNTER counts the number of samples lost. It saturates at 0x1F. When a complete sample is “popped” (i.e., removal of old FIFO data and shifting the samples down) from the FIFO (when the read pointer advances), OVF_COUNTER is reset to zero.

FIFO Read Pointer

The FIFO Read Pointer points to the location from where the processor gets the next sample from the FIFO through the I_2C interface. This advances each time a sample is popped from the FIFO. The processor can also write to this pointer after reading the samples to allow rereading samples from the FIFO if there is a data communication error.

FIFO Data Register

The circular FIFO depth is 32 and can hold up to 32 samples of data. The sample size depends on the number of LED channels (a.k.a. channels) configured as active. As each channel signal is stored as a 3-byte data signal, the FIFO width can be 3 bytes, 6 bytes, 9 bytes, or 12 bytes in size. The FIFO_DATA register in the I^2C register map points to the next sample to be read from the FIFO. FIFO_RD_PTR points to this sample. Reading FIFO_DATA register, does not automatically increment the I^2C register address. Burst reading this register, reads the same address over and over.

Each sample is 3 bytes of data per channel (i.e., 3 bytes for RED, 3 bytes for IR, etc.). The FIFO registers (0x04–0x07) can all be written and read, but in practice only the FIFO_RD_PTR register should be written to in operation. The others are automatically incremented or filled with data by the MAX30101. When starting a new SpO_2 or heart rate conversion, it is recommended to first clear the FIFO_WR_PTR, OVF_COUNTER, and FIFO_RD_PTR registers to all zeroes (0x00) to ensure the FIFO is empty and in a known state. When reading the MAX30101 registers in one burst-read I^2C transaction, the register address pointer typically increments so that the next byte of data sent is from the next register, etc. The exception to this is the FIFO data register, register 0x07. When reading this register, the address pointer does not increment, but the FIFO_RD_PTR does. So the next byte of data sent represents the next byte of data available in the FIFO.

Reading from the FIFO

Normally, reading registers from the I^2C interface autoincrements the register address pointer, so that all the registers can be read in a burst read without an I^2C start event. In the MAX30101, this holds true for all registers except for the FIFO_DATA register (register 0x07). Reading the FIFO_DATA register does not automatically increment the register address. Burst reading this register reads data from the same address over and over. Each sample comprises multiple bytes of data, so multiple bytes should be read from this register (in the same transaction) to get one full sample. The other exception is 0xFF. Reading more bytes after the 0xFF register does not advance the address pointer back to 0x00, and the data read is not meaningful.

FIFO Data Structure

The data FIFO consists of a 32-sample memory bank that can store GREEN, IR, and RED ADC data. Since each sample consists of three channels of data, there are 9 bytes of data for each sample, and therefore 288 total bytes of data can be stored in the FIFO.

The FIFO data is left-justified, as shown in Table 1; in other words, the MSB bit is always in the bit 17 data position, regardless of ADC resolution setting. See Table 2 for a visual presentation of the FIFO data structure.

Table 1. FIFO Data is Left-Justified

ADC Resolution	FIFO_DATA[17]	FIFO_DATA[16]	...	FIFO_DATA[12]	FIFO_DATA[11]	FIFO_DATA[10]	FIFO_DATA[9]	FIFO_DATA[8]	FIFO_DATA[7]	FIFO_DATA[6]	FIFO_DATA[5]	FIFO_DATA[4]	FIFO_DATA[3]	FIFO_DATA[2]	FIFO_DATA[1]	FIFO_DATA[0]
18-bit	■	■	■	■	■	■	■	■	■	■	■	■	■	■	■	■
17-bit	■	■	■	■	■	■	■	■	■	■	■	■	■	■	■	
16-bit	■	■	■	■	■	■	■	■	■	■	■	■	■	■		
15-bit	■	■	■	■	■	■	■	■	■	■	■	■	■			

FIFO Data Contains 3 Bytes per Channel

The FIFO data is left-justified, meaning that the MSB is always in the same location regardless of the ADC resolution setting. FIFO DATA[18] – [23] are not used. Table 2 shows the structure of each triplet of bytes (containing the 18-bit ADC data output of each channel). Each data sample in SpO_2 mode comprises two data triplets (3 bytes each), To read one sample, requires an I^2C read command for each byte. Thus, to read one sample in SpO_2 mode, requires 6 I^2C byte reads. To read one sample with three LED channels requires 9 I^2C byte reads. The FIFO read pointer is automatically incremented after the first byte of each sample is read.

Write/Read Pointers

Write/Read pointers are used to control the flow of data in the FIFO. The write pointer increments every time a new sample is added to the FIFO. The read pointer is incremented every time a sample is read from the FIFO. To reread a sample from the FIFO, decrement its value by one and read the data register again.

The FIFO write/read pointers should be cleared (back to 0x00) upon entering SpO_2 mode or HR mode, so that there is no old data represented in the FIFO. The pointers are automatically cleared if V_{DD} is power-cycled or V_{DD} drops below its UVLO voltage.

Table 2. FIFO Data (3 Bytes per Channel)

BYTE 1							FIFO_ DATA[17]	FIFO_ DATA[16]
BYTE 2	FIFO_ DATA[15]	FIFO_ DATA[14]	FIFO_ DATA[13]	FIFO_ DATA[12]	FIFO_ DATA[11]	FIFO_ DATA[10]	FIFO_ DATA[9]	FIFO_ DATA[8]
BYTE 3	FIFO_ DATA[7]	FIFO_ DATA[6]	FIFO_ DATA[5]	FIFO_ DATA[4]	FIFO_ DATA[3]	FIFO_ DATA[2]	FIFO_ DATA[1]	FIFO_ DATA[0]

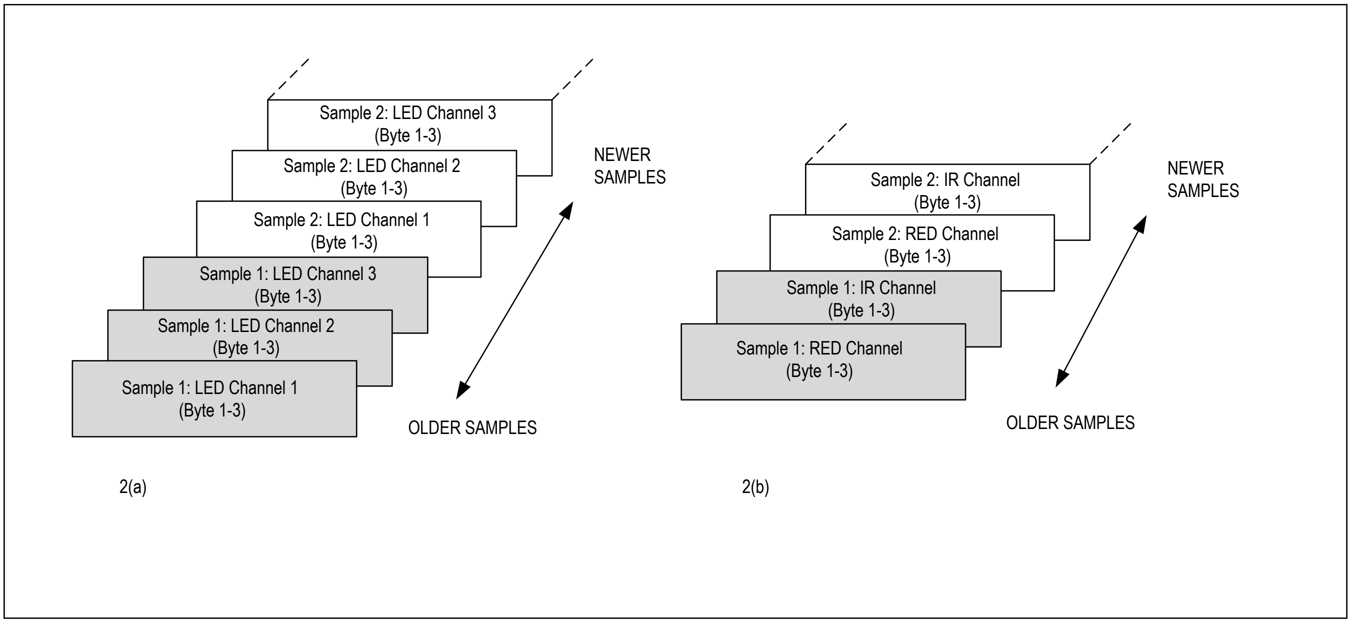

Figure 2.a and 2b. Graphical Representation of the FIFO Data Register. The left shows three LEDs in multi-LED mode, and the right shows IR and Red only in SpO_2 Mode.

Pseudo-Code Example of Reading Data from FIFO

First transaction:Get the FIFO_WR_PTR:

START;
end device address + write mode Send address of FIFO_WR_PTR;
REPEATED_START;
Send device address + read mode
Read FIFO_WR_PTR;
STOP;

The central processor evaluates the number of samples to be read from the FIFO:

NUM_AVAILABLE_SAMPLES = FIFO_WR_PTR – FIFO_RD_PTR
(Note: pointer wrap around should be taken into account)
NUM_SAMPLES_TO_READ = < less than or equal to NUM_AVAILABLE_SAMPLES >

Second transaction: Read NUM_SAMPLES_TO_READ samples from the FIFO:

```
START;
Send device address + write mode
Send address of FIFO_DATA;
REPEATED_START;
Send device address + read mode
for (i = 0; i < NUM_SAMPLES_TO_READ; i++) {
Read FIFO_DATA;
Save LED1[23:16];
Read FIFO_DATA;
Save LED1[15:8];
Read FIFO_DATA;
Save LED1[7:0];
Read FIFO_DATA;
Save LED2[23:16];
Read FIFO_DATA;
Save LED2[15:8];
Read FIFO_DATA;
Save LED2[7:0];
Read FIFO_DATA;
Save LED3[23:16];
Read FIFO_DATA;
Save LED3[15:8];
Read FIFO_DATA;
Save LED3[7:0];
Read FIFO_DATA;
}
STOP;
START;
Send device address + write mode
Send address of FIFO_RD_PTR;

Write FIFO_RD_PTR;
STOP;
```

Third transaction: Write to FIFO_RD_PTR register. If the second transaction was successful, FIFO_RD_PTR points to the next sample in the FIFO, and this third transaction is not necessary. Otherwise, the processor updates the FIFO_RD_PTR appropriately, so that the samples are reread.

FIFO Configuration (0x08)

REGISTER	B7	B6	B5	B4	B3	B2	B1	B0	REG ADDR	POR STATE	R/W
FIFO Configuration	SMP_AVE[2:0]			FIFO_ROL LOVER_EN	FIFO_A_FULL[3:0]				0x08	0x00	R/W

Bits 7:5: Sample Averaging (SMP_AVE)

To reduce the amount of data throughput, adjacent samples (in each individual channel) can be averaged and decimated on the chip by setting this register.

Table 3. Sample Averaging

SMP_AVE[2:0]	NO. OF SAMPLES AVERAGED PER FIFO SAMPLE
000	1 (no averaging)
001	2
010	4
011	8
100	16
101	32
110	32
111	32

Bit 4: FIFO Rolls on Full (FIFO_ROLLOVER_EN)

This bit controls the behavior of the FIFO when the FIFO becomes completely filled with data. If FIFO_ROLLOVER_EN is set (1), the FIFO Address rolls over to zero and the FIFO continues to fill with new data. If the bit is not set (0), then the FIFO is not updated until FIFO_DATA is read or the WRITE/READ pointer positions are changed.

Bits 3:0: FIFO Almost Full Value (FIFO_A_FULL)

This register sets the number of data samples (3 bytes/sample) remaining in the FIFO when the interrupt is issued. For example, if this field is set to 0x0, the interrupt is issued when there is 0 data samples remaining in the FIFO (all 32 FIFO words have unread data). Furthermore, if this field is set to 0xF, the interrupt is issued when 15 data samples are remaining in the FIFO (17 FIFO data samples have unread data).

FIFO_A_FULL[3:0]	EMPTY DATA SAMPLES IN FIFO WHEN INTERRUPT IS ISSUED	UNREAD DATA SAMPLES IN FIFO WHEN INTERRUPT IS ISSUED
0x0h	0	32
0x1h	1	31
0x2h	2	30
0x3h	3	29
…	…	...
0xFh	15	17

Mode Configuration (0x09)

REGISTER	B7	B6	B5	B4	B3	B2	B1	B0	REG ADDR	POR STATE	R/W
Mode Configuration	SHDN	RESET				MODE[2:0]			0x09	0x00	R/W

Bit 7: Shutdown Control (SHDN)

The part can be put into a power-save mode by setting this bit to one. While in power-save mode, all registers retain their values, and write/read operations function as normal. All interrupts are cleared to zero in this mode.

Bit 6: Reset Control (RESET)

When the RESET bit is set to one, all configuration, threshold, and data registers are reset to their power-on-state through a power-on reset. The RESET bit is cleared automatically back to zero after the reset sequence is completed. Note: Setting the RESET bit does not trigger a PWR_RDY interrupt event.

Bits 2:0: Mode Control

These bits set the operating state of the MAX30101. Changing modes does not change any other setting, nor does it erase any previously stored data inside the data registers.

Table 4. Mode Control

MODE[2:0]	MODE	ACTIVE LED CHANNELS
000	Do not use	
001	Do not use	
010	Heart Rate mode	Red only
011	SpO2 mode	Red and IR
100–110	Do not use	
111	Multi-LED mode	Green, Red, and/or IR

SpO_2 Configuration (0x0A)

REGISTER	B7	B6	B5	B4	B3	B2	B1	B0	REG ADDR	POR STATE	R/W
SpO2 Configuration		SPO2_ADC_RGE[1:0]		SPO2_SR[2:0]			LED_PW[1:0]		0x0A	0x00	R/W

Bits 6:5: SpO_2 ADC Range Control

This register sets the SpO_2 sensor ADC's full-scale range as shown in Table 5.

Table 5. SpO2 ADC Range Control (18-Bit Resolution)

SPO2_ADC_RGE[1:0]	LSB SIZE (pA)	FULL SCALE (nA)
00	7.81	2048
01	15.63	4096
02	31.25	8192
03	62.5	16384

Bits 4:2: SpO_2 Sample Rate Control

These bits define the effective sampling rate with one sample consisting of one IR pulse/conversion, one RED pulse/conversion, and one GREEN pulse/conversion. The sample rate and pulse-width are related in that the sample rate sets an upper bound on the pulse-width time. If the user selects a sample rate that is too high for the selected LED_PW setting, the highest possible sample rate is programmed instead into the register.

Table 6. SpO_2 Sample Rate Control

SPO2_SR[2:0]	SAMPLES PER SECOND
000	50
001	100
010	200
011	400
100	800
101	1000
110	1600
111	3200

See Table 15 and Table 16 for Pulse-Width vs. Sample Rate information.

Bits 1:0: LED Pulse Width Control and ADC Resolution

These bits set the LED pulse width (the IR, Red, and Green have the same pulse width), and, therefore, indirectly sets the integration time of the ADC in each sample. The ADC resolution is directly related to the integration time.

Table 7. LED Pulse Width Control

LED_PW[1:0]	PULSE WIDTH (µs)	ADC RESOLUTION (bits)
00	69 (68.95)	15
01	118 (117.78)	16
10	215 (215.44)	17
11	411 (410.75)	18

LED Pulse Amplitude (0x0C–0x0F)

REGISTER	B7	B6	B5	B4	B3	B2	B1	B0	REG ADDR	POR STATE	R/W
LED Pulse Amplitude	LED1_PA[7:0]								0x0C	0x00	R/W
	LED2_PA[7:0]								0x0D	0x00	R/W
	LED3_PA[7:0]								0x0E	0x00	R/W
	LED4_PA[7:0]								0x0F	0x00	R/W

These bits set the current level of each LED as shown in Table 8

Table 8. LED Current Control

LEDx_PA [7:0]	TYPICAL LED CURRENT (mA)*
0x00h	0.0
0x01h	0.2
0x02h	0.4
…	…
0x0Fh	3.0
…	…
0x1Fh	6.2
…	…
0x3Fh	12.6
…	…
0x7Fh	25.4
…	…
0xFFh	51.0

.*Actual measured LED current for each part can vary significantly due to the trimming methodology.

Multi-LED Mode Control Registers (0x11–0x12)

REGISTER	B7	B6	B5	B4	B3	B2	B1	B0	REG ADDR	POR STATE	R/W
Multi-LED Mode Control Registers		SLOT2[2:0]				SLOT1[2:0]			0x11	0x00	R/W
		SLOT4[2:0]				SLOT3[2:0]			0x12	0x00	R/W

In multi-LED mode, each sample is split into up to four time slots, SLOT1 through SLOT4. These control registers determine which LED is active in each time slot, making for a very flexible configuration.

Table 9. Multi-LED Mode Control Registers

SLOTx[2:0] Setting	WHICH LED IS ACTIVE	LED PULSE AMPLITUDE SETTING
000	None (time slot is disabled)	N/A (Off)
001	LED1 (RED)	LED1_PA[7:0]
010	LED2 (IR)	LED2_PA[7:0]
011*	LED3 (GREEN)	LED3_PA[7:0]
	LED4 (GREEN)	LED4_PA[7:0]
100	None	N/A (Off)
101	RESERVED	RESERVED
110	RESERVED	RESERVED
111	RESERVED	RESERVED

Each slot generates a 3-byte output into the FIFO. One sample comprises all active slots, for example if SLOT1 and SLOT2 are non-zero, then one sample is 2 x 3 = 6 bytes. If SLOT1 through SLOT3 are all non-zero, then one sample is 3 x 3 = 9 bytes. The slots should be enabled in order (i.e., SLOT1 should not be disabled if SLOT2 or SLOT3 are enabled). *Both LED3 and LED4 are wired to Green LED. Green LED sinks current out of LED3_PA[7:0] and LED4_PA[7:0] configurationin Multi-LED Mode and SLOTx[2:0] = 011.

Temperature Data (0x1F–0x21)

REGISTER	B7	B6	B5	B4	B3	B2	B1	B0	REG ADDR	POR STATE	R/W
Temp_Integer	TINT[7]								0x1F	0x00	R/W
Temp_Fraction					TFRAC[3:0]				0x20	0x00	R/W
Die Temperature Config								TEMP_EN	0x21	0x00	R/W

Temperature Integer

The on-board temperature ADC output is split into two registers, one to store the integer temperature and one to store the fraction. Both should be read when reading the temperature data, and the equation below shows how to add the two registers together:

$T_{MEASURED} = T_{INTEGER} + T_{FRACTION}$

This register stores the integer temperature data in 2's complement format, where each bit corresponds to 1°C.

Table 10. Temperature Integer

REGISTER VALUE (hex)	TEMPERATURE (°C)
0x00	0
0x00	+1
...	...
0x7E	+126
0x7F	+127
0x80	-128
0x81	-127
...	...
0xFE	-2
0xFF	-1

Temperature Fraction

This register stores the fractional temperature data in increments of 0.0625°C. If this fractional temperature is paired with a negative integer, it still adds as a positive fractional value (e.g., -128°C + 0.5°C = -127.5°C).

Temperature Enable (TEMP_EN)

This is a self-clearing bit which, when set, initiates a single temperature reading from the temperature sensor. This bit clears automatically back to zero at the conclusion of the temperature reading when the bit is set to one.

Timing for Measurements and Data Collection

Slot Timing in Multi-LED Modes

The MAX30101 can support up to three LED channels of sequential processing (Red, IR, and Green). In multi-LED modes, a time slot or period exists between active sequential channels. Table 11 displays the four possible channel slot times associated with each pulse width setting. [[Figure 3. Channel Slot Timing for the SpO_2 Mode with a 1kHz Sample Rate]] shows an example of channel slot timing for a SpO_2 mode application with a 1kHz sample rate.

Table 11. Slot Timing

PULSE-WIDTH SETTING (µs)	CHANNEL SLOT TIMING (TIMING PERIOD BETWEEN PULSES) (µs)	CHANNEL-CHANNEL TIMING (RISING EDGE-TO-RISING EDGE) (µs)
69	358	427
118	407	525
215	505	720
411	696	1107

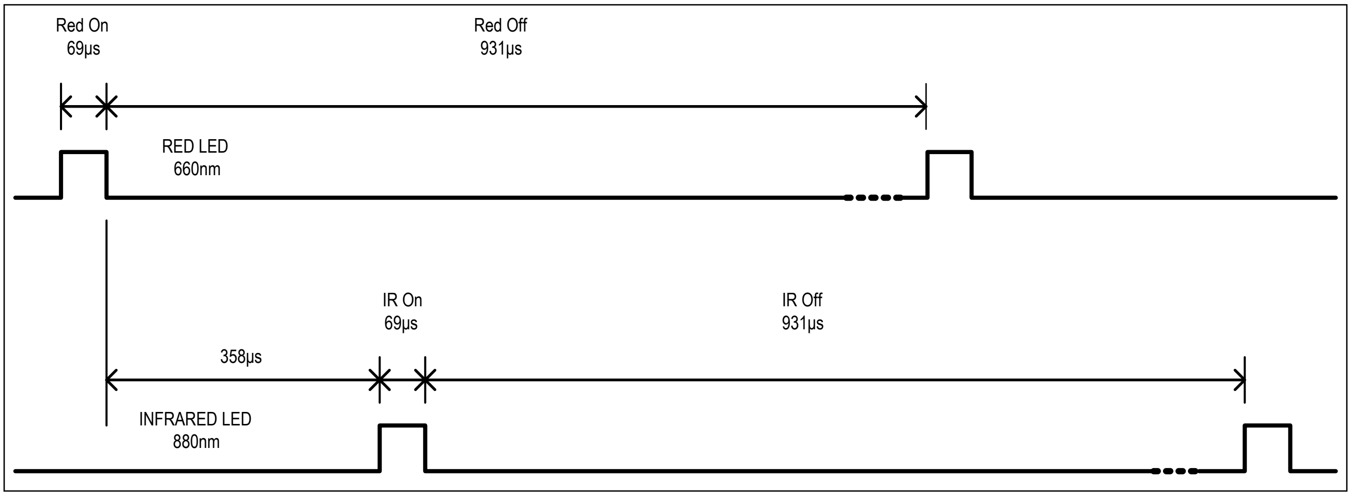

Figure 3. Channel Slot Timing for the SpO2 Mode with a 1kHz Sample Rate

Timing in SpO_2 Mode

The internal FIFO stores up to 32 samples, so that the system processor does not need to read the data after every sample. SpO_2 can be calibrated using temperature

data. In this case, the temperature does not need to be sampled very often – once a second or every few seconds should be sufficient.

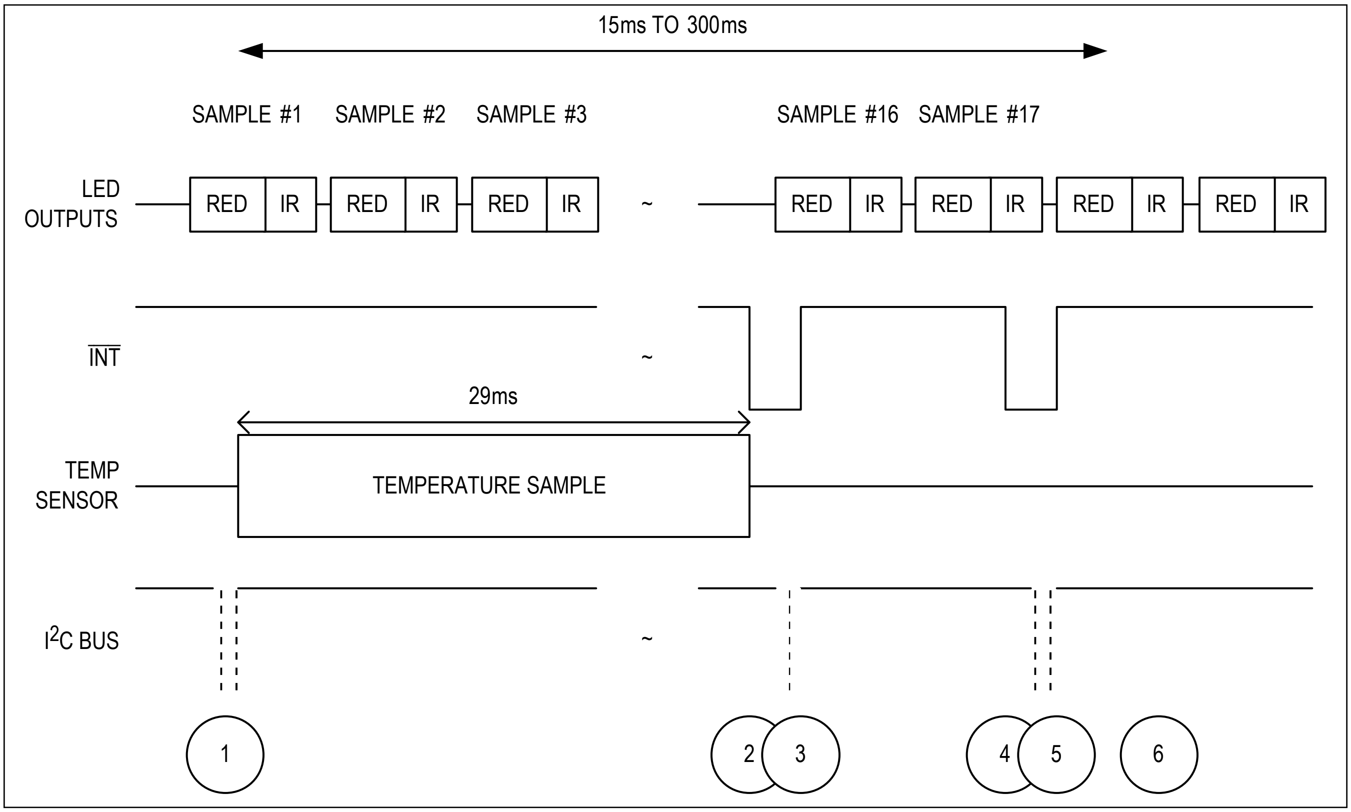

Figure 4. Timing for Data Acquisition and Communication When in SpO_2 Mode

Table 12. Events Sequence for Figure 4 in SpO2 Mode

EVENT	DESCRIPTION	COMMENTS
1	Enter into SpO2 Mode. Initiate a Temperature measurement.	I2C Write Command sets MODE[2:0] = 0x03 and set A_FULL_EN. Then, to enable and initiate a single temperature measurement, set TEMP_EN and DIE_TEMP_RDY_EN.
2	Temperature Measurement Complete, Interrupt Generated	DIE_TEMP_RDY interrupt triggers, alerting the central processor to read the data.
3	Temp Data is Read, Interrupt Cleared	
4	FIFO is Almost Full, Interrupt Generated	Interrupt is generated when the FIFO almost full threshold is reached.
5	FIFO Data is Read, Interrupt Cleared	
6	Next Sample is Stored	New Sample is stored at the new read pointer location. Effectively, it is now the first sample in the FIFO.

Timing in HR Mode

The internal FIFO stores up to 32 samples, so that the system processor does not need to read the data after every sample. In HR mode (Figure 5), unlike in SpO_2 mode, temperature information is not necessary to interpret the data. The user can select either the Red, IR, or Green LED channel for heart rate.

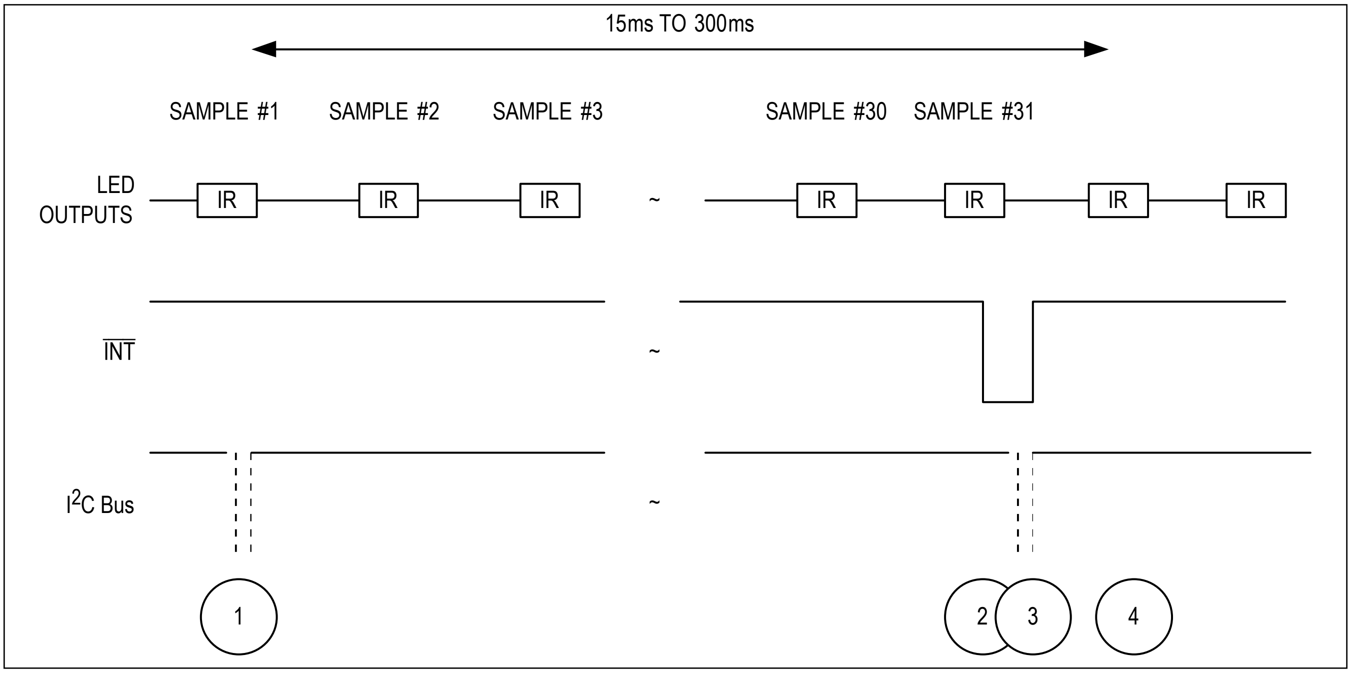

Figure 5. Timing for Data Acquisition and Communication When in HR Mode

Table 13. Events Sequence for Figure 5 in HR Mode

EVENT	DESCRIPTION	COMMENTS
1	Enter into Mode	I2C Write Command sets MODE[2:0] = 0x02. Mask the A_FULL_EN Interrupt.
2	FIFO is Almost Full, Interrupt Generated	Interrupt is generated when the FIFO has only one empty space left.
3	FIFO Data is Read, Interrupt Cleared	
4	Next Sample is Stored	New sample is stored at the new read pointer location. Effectively, it is now the first sample in the FIFO.

Power Sequencing and Requirements

Power-Up Sequencing

Figure 6 shows the recommended power-up sequence for the MAX30101. It is recommended to power the V_{DD} supply first, before the LED power supplies (V_{LED+}). The interrupt and I^2C pins can be pulled up to an external voltage even when the power supplies are not powered up. After the power is established, an interrupt occurs to alert the system that the MAX30101 is ready for operation. Reading the I_2C interrupt register clears the interrupt, as shown in the Figure 6.

Power-Down Sequencing

The MAX30101 is designed to be tolerant of any power supply sequencing on power-down.

I2C Interface

The MAX30101 features an I2C/SMBus-compatible, 2-wire serial interface consisting of a serial data line (SDA) and a serial clock line (SCL). SDA and SCL facilitate communication between the MAX30101 and the master at clock rates up to 400kHz. Figure 1 shows the 2-wire interface timing diagram. The master generates SCL and initiates data transfer on the bus. The master device writes data to the MAX30101 by transmitting the proper slave address followed by data. Each transmit sequence is framed by a START (S) or REPEATED START (Sr) condition and a STOP (P) condition. Each word transmitted to the MAX30101 is 8 bits long and is followed by an acknowledge clock pulse. A master reading data from the MAX30101 transmits the proper slave address followed by a series of nine SCL pulses.

The MAX30101 transmits data on SDA in sync with the master-generated SCL pulses. The master acknowledges receipt of each byte of data. Each read sequence is framed by a START (S) or REPEATED START (Sr) condition, a not acknowledge, and a STOP (P) condition. SDA operates as both an input and an open-drain output. A pullup resistor, typically greater than 500Ω, is required on SDA. SCL operates only as an input. A pullup resistor, typically greater than 500Ω, is required on SCL if there are multiple masters on the bus, or if the single master has an open-drain SCL output. Series resistors in line with SDA and SCL are optional. Series resistors protect the digital inputs of the MAX30101 from high voltage spikes on the bus lines and minimize crosstalk and undershoot of the bus signals.

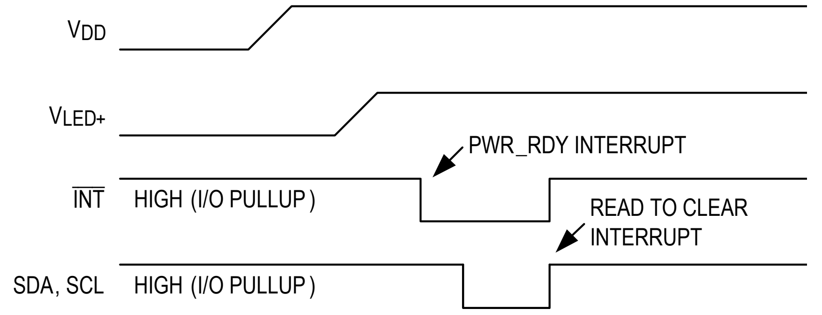

Figure 6. Power-Up Sequence of the Power Supply Rails

Bit Transfer

One data bit is transferred during each SCL cycle. The data on SDA must remain stable during the high period of the SCL pulse. Changes in SDA while SCL is high are control signals. See the *START and STOP Conditions* section.

START and STOP Conditions

SDA and SCL idle high when the bus is not in use. A master initiates communication by issuing a START condition. A START condition is a high-to-low transition on SDA with SCL high. A STOP condition is a low-to-high transition on SDA while SCL is high (Figure 7). A START condition from the master signals the beginning of a transmission to the MAX30101. The master terminates transmission, and frees the bus, by issuing a STOP condition. The bus remains active if a REPEATED START condition is generated instead of a STOP condition.

Early STOP Conditions

The MAX30101 recognizes a STOP condition at any point during data transmission except if the STOP condition occurs in the same high pulse as a START condition. For proper operation, do not send a STOP condition during the same SCL high pulse as the START condition.

Slave Address

A bus master initiates communication with a slave device by issuing a START condition followed by the 7-bit slave ID. When idle, the MAX30101 waits for a START condition followed by its slave ID. The serial interface compares each slave ID bit by bit, allowing the interface to power down and disconnect from SCL immediately if an incorrect slave ID is detected. After recognizing a START condition followed by the correct slave ID, the MAX30101 is programmed to accept or send data. The LSB of the slave ID word is the read/write (R/W) bit. R/W indicates whether the master is writing to or reading data from the MAX30101 (R/W = 0 selects a write condition, R/W = 1 selects a read condition). After receiving

the proper slave ID, the MAX30101 issues an ACK by pulling SDA low for one clock cycle.

The MAX30101 slave ID consists of seven fixed bits, B7–B1 (set to 0b1010111). The most significant slave ID bit (B7) is transmitted first, followed by the remaining bits. Table 14 shows the possible slave IDs of the device.

Acknowledge

The acknowledge bit (ACK) is a clocked 9th bit that the MAX30101 uses to handshake receipt each byte of data when in write mode (Figure 8). The MAX30101 pulls down SDA during the entire master-generated 9th clock pulse if the previous byte is successfully received. Monitoring ACK allows for detection of unsuccessful data transfers. An unsuccessful data transfer occurs if a receiving device is busy or if a system fault has occurred. In the event of an unsuccessful data transfer, the bus master retries communication. The master pulls down SDA during the 9th clock cycle to acknowledge receipt of data when the MAX30101 is in read mode. An acknowledge is sent by the master after each read byte to allow data transfer to continue. A not-acknowledge is sent when the master reads the final byte of data from the MAX30101, followed by a STOP condition.

Write Data Format

For the write operation, send the slave ID as the first byte followed by the register address byte and then one or more data bytes. The register address pointer increments automatically after each byte of data received, so for example the entire register bank can be written by at one time. Terminate the data transfer with a STOP condition. The write operation is shown in Figure 9.

The internal register address pointer increments automatically, so writing additional data bytes fill the data registers in order.

Table 14. Slave ID Description

B7	B6	B5	B4	B3	B2	B1	B0	WRITE ADDRESS	READ ADDRESS
1	0	1	0	1	1	1	RW	0xAE	0xAF

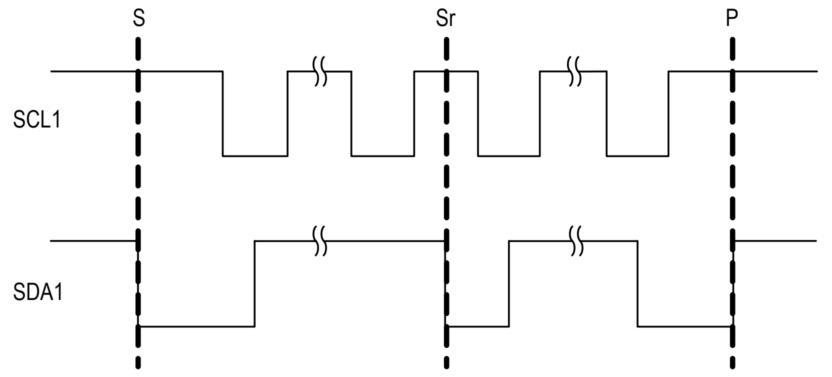

Figure 7. START, STOP, and REPEATED START Conditions

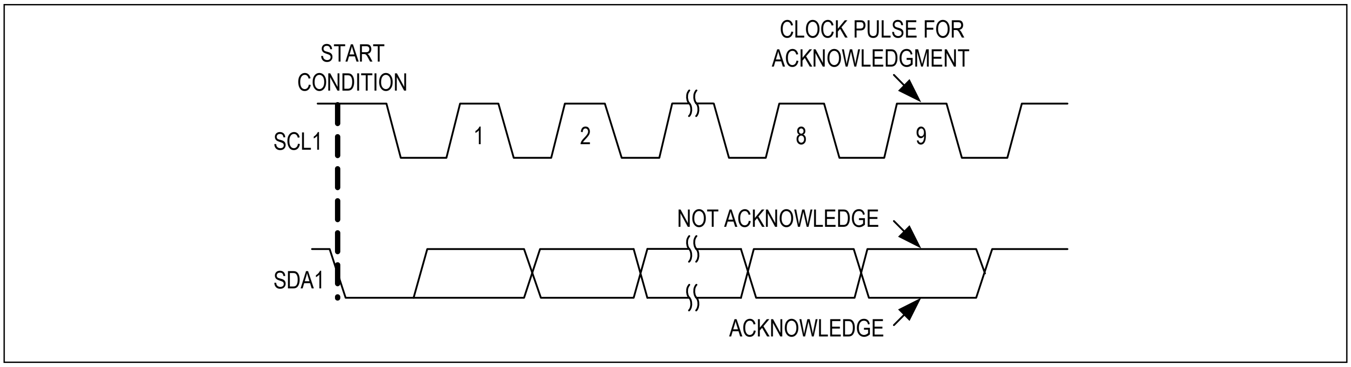

Figure 8. Acknowledge

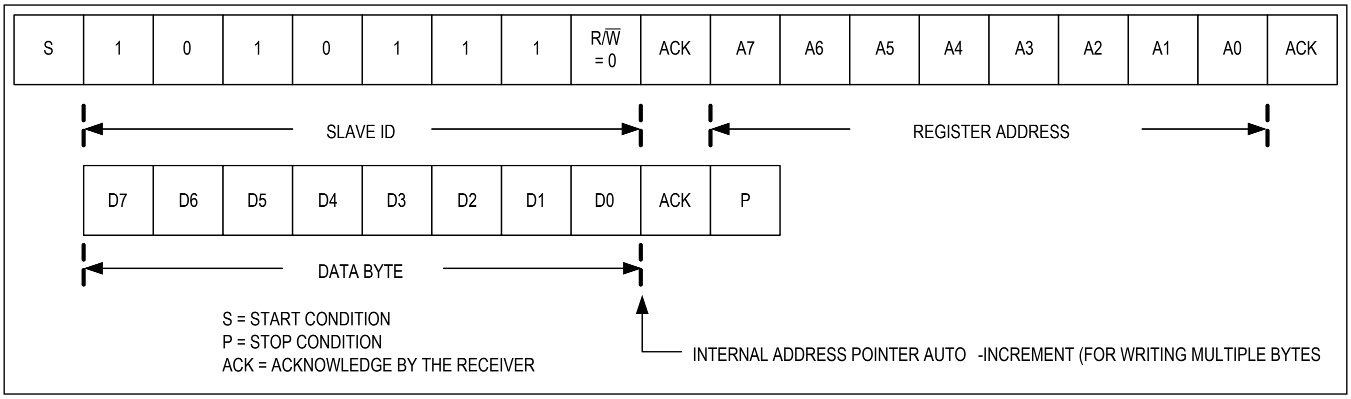

Figure 9. Writing One Data Byte to the MAX30101

Read Data Format

For the read operation, two I^2C operations must be performed. First, the slave ID byte is sent followed by the I^2C register that you wish to read. Then a REPEAT START (Sr) condition is sent, followed by the read slave ID. The MAX30101 then begins sending data beginning with the register selected in the first operation. The read pointer increments automatically, so the MAX30101 continues sending data from additional registers in sequential order until a STOP (P) condition is received. The exception to this is the FIFO_DATA register, at which the read pointer no longer increments when reading additional bytes. To read the next register after FIFO_DATA, an I^2C write command is necessary to change the location of the read pointer. Figure 10 show the process of reading one byte or multiple bytes of data. An initial write operation is required to send the read register address.

Data is sent from registers in sequential order, starting from the register selected in the initial I^2C write operation. If the FIFO_DATA register is read, the read pointer will not automatically increment, and subsequent bytes of data will contain the contents of the FIFO.

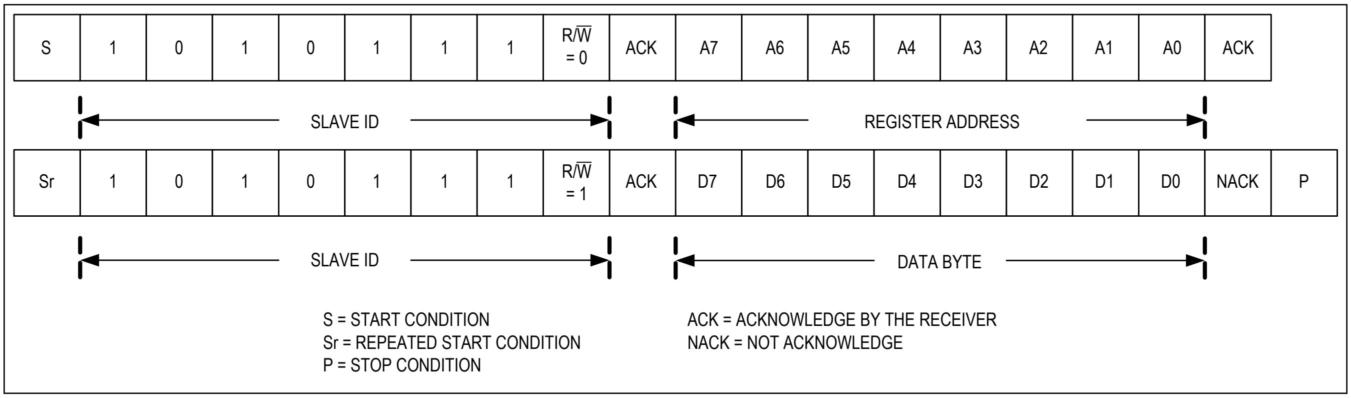

Figure 10. Reading one byte of data from MAX30101

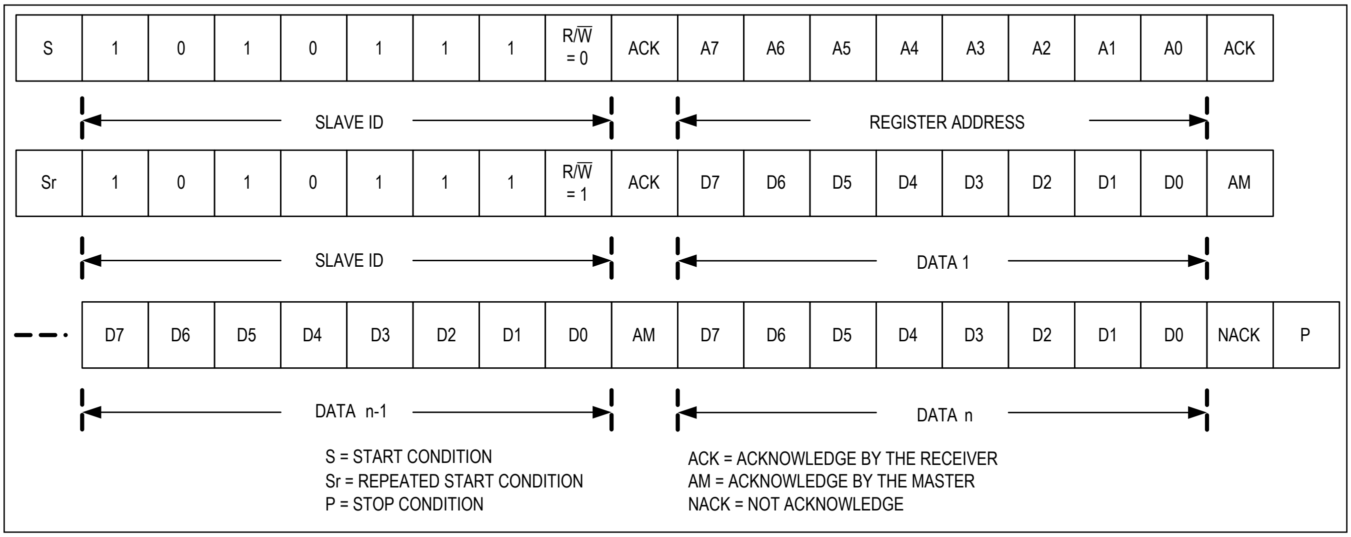

Figure 11. Reading multiple bytes of data from the MAX30101

Applications Information

Soldering and Cleaning Recommendations

The MAX30101 comes in an OLGA package that is not sealed from dust or liquid. Because of this, the MAX30101 requires special care to install on a board.

If possible, the MAX30101 should be the last component installed on the board. Install the MAX30101 after the board ultrasonic cleaning is completed. When soldering the MAX30101, use a low-residue, no-clean solder paste. The MAX30101 should not be cleaned with a liquid solution, baked, or coated with anything.

The *Application Note 6381* serves as a guide for handling the OLGA package when manufacturing a board.

Sampling Rate and Performance

The maximum sample rate for the ADC depends on the selected pulse-width, which in turn, determines the ADC resolution. For instance, if the pulse-width is set to 69µs then the ADC resolution is 15 bits, and all sample rates are selectable. However, if the pulse-width is set to 411µs, then the samples rates are limited. The allowed sample rates for both SpO_2 and HR Modes are summarized in the Table 15 and Table 16:

Power Considerations

The LED waveforms and their implication for power supply design are discussed in this section.

The LEDs in the MAX30101 are pulsed with a low duty cycle for power savings, and the pulsed currents can cause ripples in the V_{LED+} power supply. To ensure these pulses do not translate into optical noise at the LED outputs, the power supply must be designed to handle these. Ensure that the resistance and inductance from the power supply (battery, DC-DC converter, or LDO) to the pin is much smaller than 1Ω, and that there is at least 1µF of power-supply bypass capacitance to a good ground plane. The capacitance should be located as close as physically possible to the IC.

Table 15. SpO_2 Mode (Allowed Settings)

SAMPLES PER SECOND	PULSE WIDTH (µs)			
**69**	**118**	**215**	**411**	
50	O	O	O	O
100	O	O	O	O
200	O	O	O	O
400	O	O	O	O
800	O	O	O	
1000	O	O		
1600	O			
3200				
Resolution (bits)	15	16	17	18

Table 16. HR Mode (Allowed Settings)

SAMPLES PER SECOND	PULSE WIDTH (µs)			
**69**	**118**	**215**	**411**	
50	O	O	O	O
100	O	O	O	O
200	O	O	O	O
400	O	O	O	O
800	O	O	O	O
1000	O	O	O	O
1600	O	O	O	
3200	O			
Resolution (bits)	15	16	17	18

SpO_2 Temperature Compensation

The MAX30101 has an accurate on-board temperature sensor that digitizes the IC's internal temperature upon command from the I^2C master. The temperature has an effect on the wavelength of the red and IR LEDs. While the device output data is relatively insensitive to the wavelength of the IR LED, the red LED's wavelength is critical to correct interpretation of the data.

Table 17 shows the correlation of red LED wavelength versus the temperature of the LED. Since the LED die heats up with a very short thermal time constant (tens of microseconds), the LED wavelength should be calculated according to the current level of the LED and the temperature of the IC. Use Table 17 to estimate the temperature.

Table 17. RED LED Current Settings vs. LED Temperature Rise

RED LED CURRENT SETTING	RED LED DUTY CYCLE (% OF LED PULSE-WIDTH TO SAMPLE TIME)	ESTIMATED TEMPERATURE RISE (ADD TO TEMP SENSOR MEASUREMENT) (°C)
00000001 (0.2mA)	8	0.1
11111010 (50mA)	8	2
00000001 (0.2mA)	16	0.3
11111010 (50mA)	16	4
00000001 (0.2mA)	32	0.6
11111010 (50mA)	32	8

Red LED Current Settings vs. LED Temperature Rise

Add this to the module temperature reading to estimate the LED temperature and output wavelength. The LED temperature estimate is valid even with very short pulse-widths, due to the fast thermal time constant of the LED.

Interrupt Pin Functionality

The active-low interrupt pin pulls low when an interrupt is triggered. The pin is open-drain, which means it normally requires a pullup resistor or current source to an external voltage supply (up to +5V from GND). The interrupt pin is not designed to sink large currents, so the pullup resistor value should be large, such as 4.7kΩ.

Typical Application Circuits

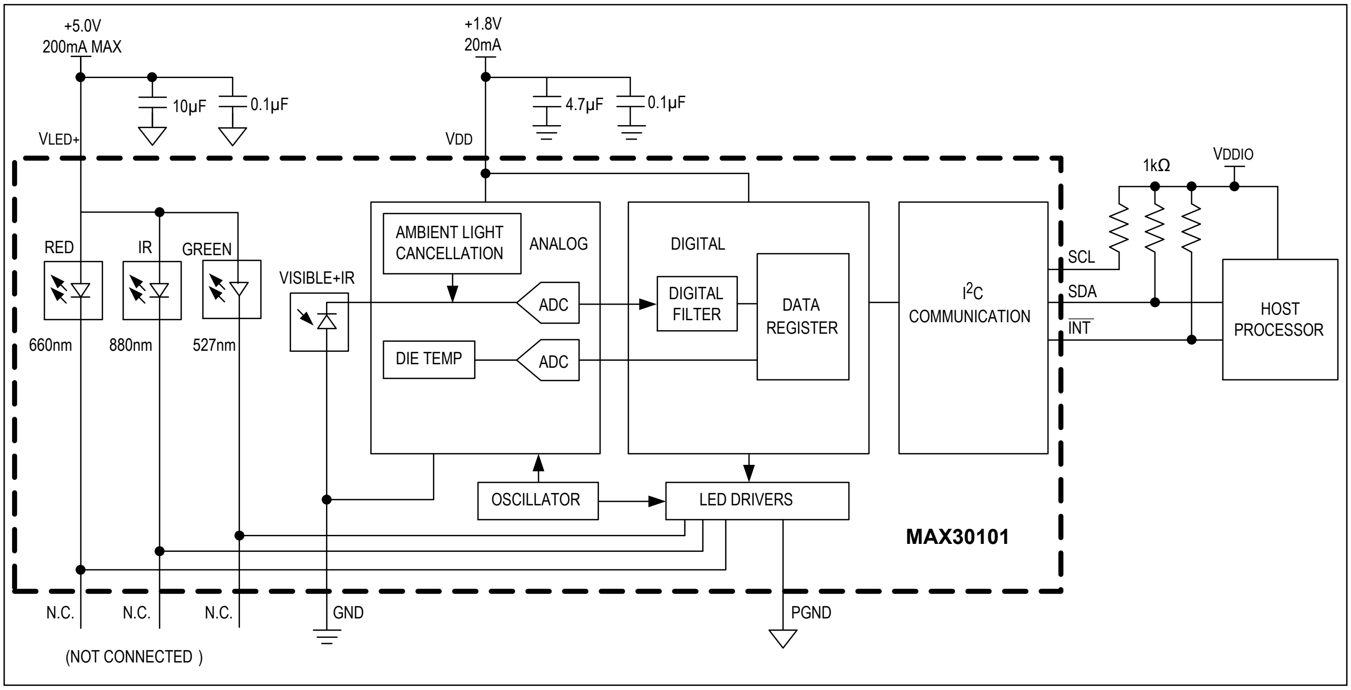

Ordering Information

PART	TEMP RANGE	PIN-PACKAGE
MAX30101EFD+T	-40°C to +85°C	14 OESIP<br>(0.8mm Pin Pitch)

+Denotes lead(Pb)-free/RoHS-compliant package.

T = Tape and reel.

Revision History

REVISION NUMBER	REVISION DATE	DESCRIPTION	PAGES CHANGED
0	3/16	Initial release	—
1	6/18	Changed register descriptions, updated tables 8,9,13,15,16, removed Proximity function, updated FIFO_A_FULL description table	10–15, 18, 21–25, 27, 28
2	9/18	Updated the *Applications, Absolute Maximum Ratings, Electrical Characteristics, Pin Description*, and *Power-Up Sequencing* sections; updated the *System Diagram, Pin Configuration, Functional Diagram*, and *Typical Application Circuit*; updated the *Register Maps and Descriptions, Mode Configuration (0x09),* SpO_2 *Configuration (0x0A), LED Pulse Amplitude (0x0C–0x0F)*, Table 8, and Table 9.	1–5, 9–11,19, 21–22, 29, 32
3	6/20	Updated SpO_2 *Sample Rate Control[2:4] (0x0A)* and *Applications Information* section	24, 34

For pricing, delivery, and ordering information, please visit Maxim Integrated's online storefront at https://www.maximintegrated.com/en/storefront/storefront.html.

Maxim Integrated cannot assume responsibility for use of any circuitry other than circuitry entirely embodied in a Maxim Integrated product. No circuit patent licenses are implied. Maxim Integrated reserves the right to change the circuitry and specifications without notice at any time. The parametric values (min and max limits) shown in the Electrical Characteristics table are guaranteed. Other parametric values quoted in this data sheet are provided for guidance.

Maxim Integrated and the Maxim Integrated logo are trademarks of Maxim Integrated Products, Inc. © 2020 Maxim Integrated Products, Inc.
