# Supplementary material for: Validation of an mHealth System for Monitoring Fundamental Physiological Parameters in the Clinical Setting
Source: Sensors (Basel). 2024 Aug 10;24(16):5164. doi: 10.3390/s24165164 (PMC11359666; doi:10.3390/s24165164)
Supplement: Supplementary file 1 [file sensors-24-05164-s001.zip › MAX32664.pdf]

MAX32664

Ultra-Low Power Biometric Sensor Hub

General Description

The MAX32664 is a sensor hub family with embedded firmware and world-class algorithms for wearables. It seamlessly enables customer-desired sensor functionality, including communication with Analog Devices' optical sensor solutions and delivering raw or calculated data to the outside world. This is achieved while keeping overall system power consumption in check. The device family interfaces to a microcontroller host through a fast-mode slave I^2C interface for access to raw and processed sensor data as well as field updates. A firmware bootloader is also provided.

The MAX32664 Version A supports the MAX30101/MAX30102 high-sensitivity pulse oximeter and heart-rate sensor for wearable health for finger-based applications. A master mode I^2C interface for communication with sensors is provided.

The MAX32664 Version B supports the MAX86140/MAX86141 for wrist-based applications. A master mode SPI interface for communication with sensors is provided.

The MAX32664 Version C supports the MAX86140/MAX86141 for wrist-based applications and MAXM86161 for ear-based applications. The device provides either a master mode SPI or an I^2C interface for communication with sensors.

The MAX32664 Version D supports the MAX30101/MAX30102 high-sensitivity pulse oximeter and heart-rate sensor for wearable health for finger-based applications. A master mode I^2C interface for communication with sensors is provided. Version D also supports estimated blood pressure monitoring.

The wearable algorithms in the MAX32664 sensor hub support a directly connected accelerometer. They also allow feeding of X, Y, and Z samples from a host-connected accelerometer. This architecture provides robust detection and compensation of motion artifacts in captured samples.

The tiny form factor 1.6mm x 1.6mm WLP or 3mm x 3mm TQFN allows for integration into extremely small application devices.

Applications

- Wearable Fitness
- Hearables
- Wearable Medical
- Portable Medical
- Mobile Devices

Benefits and Features

- Biometric Sensor Hub Enables Faster Time to Market
- Finger-Based (Version A) Algorithms Measure:
  - Pulse Heart Rate
  - Pulse Blood Oxygen Saturation (SpO_2)
- Wrist-Based (Version B) Algorithm Measures:
  - Pulse Heart Rate
- Wrist-Based or Ear-Based (Version C) Algorithms Measure:
  - Pulse Heart Rate
  - Pulse Blood Oxygen Saturation (SpO_2)
- Finger-Based (Version D) Algorithms Measure:
  - Pulse Heart Rate
  - Pulse Blood Oxygen Saturation (SpO_2)
  - Estimated Blood Pressure
- Both Raw and Processed Data Are Available
- Basic Peripheral Mix Optimizes Size and Performance
  - One Slave I^2C for Communication with a Host Microcontroller
  - One Master I^2C for Communication with Sensors (Versions A, C, and D)
  - One Master SPI for Communication with Sensors (Versions B and C)
  - 32.768kHz RTC
  - FIFO Provides Minimal Host Interaction
  - Bootloader Facilitates Secure, Authenticated Firmware Upgrades

Ordering Information appears at end of data sheet.

19-100313; Rev 4; 2/22

© 2022 Analog Devices, Inc. All rights reserved. Trademarks and registered trademarks are the property of their respective owners.

One Analog Way, Wilmington, MA 01887 U.S.A. | Tel: 781.329.4700 | © 2022 Analog Devices, Inc. All rights reserved.

Simplified Block Diagram

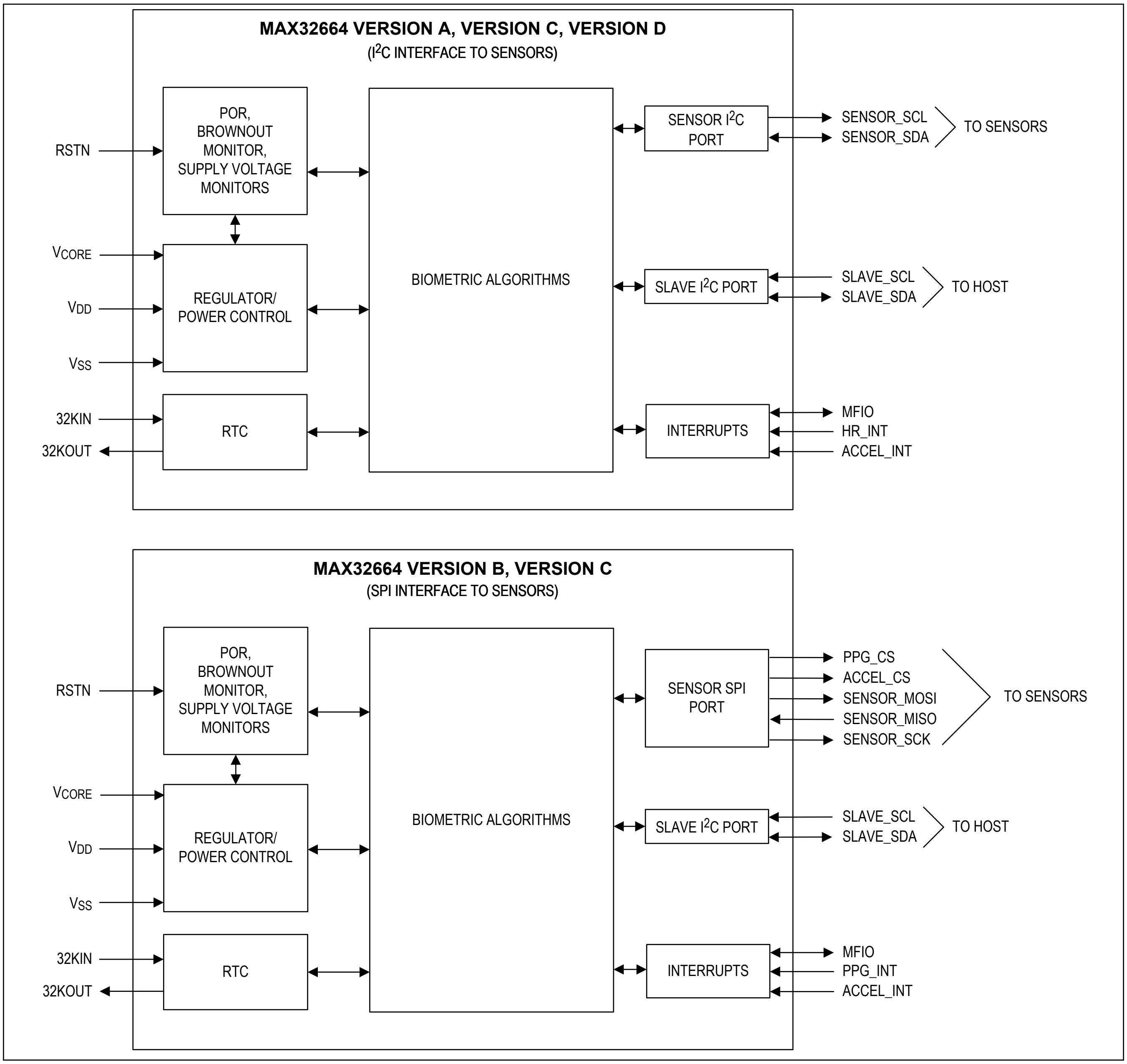

TABLE OF CONTENTS

General Description 1
Applications 1
Benefits and Features 1
Simplified Block Diagram 2
Absolute Maximum Ratings 7
Package Information 7
  16 WLP 7
  24 TQFN-EP 7
Electrical Characteristics 7
Electrical Characteristics—I^2C 9
Electrical Characteristics—SPI 9
Pin Configuration MAX32664 Version A and Version D 16-WLP I^2C Interface to Sensors 12
Pin Descriptions MAX32664 Version A and Version D 16-WLP I^2C Interface to Sensors 12
Pin Configuration MAX32664 Version B and Version C 16-WLP SPI Interface to Sensors 14
Pin Descriptions MAX32664 Version B and Version C 16-WLP SPI Interface to Sensors 14
Pin Configuration MAX32664 Version A and Version D 24-TQFN I^2C Interface to Sensors 16
Pin Descriptions MAX32664 Version A and Version D 24-TQFN I^2C Interface to Sensors 16
Pin Configuration MAX32664 Version B and Version C 24-TQFN SPI Interface to Sensors 18
Pin Descriptions MAX32664 Version B and Version C 24-TQFN SPI Interface to Sensors 18
Pin Configuration MAX32664 Version C 16-WLP I^2C Interface to Sensors 20
Pin Descriptions MAX32664 Version C 16-WLP I^2C Interface to Sensors 20
Pin Configuration MAX32664 Version C 24-TQFN I^2C Interface to Sensors 22
Pin Descriptions MAX32664 Version C 24-TQFN I^2C Interface to Sensors 22
Detailed Description 24
  Finger Heart Rate, SpO_2 Algorithm (Version A) 24
  Wrist Heart Rate Algorithm (Version B) 24
  Wrist or Ear Heart Rate, SpO_2 Algorithm (Version C) 24
  Finger Heart Rate, SpO_2, Blood Pressure Algorithm (Version D) 24
  Algorithm Selection and Evaluation 24
  Interface to Host 25
  Interface to the Sensors 25
  Device Selection 25
Applications Information 26
  Evaluation Platforms 26
Typical Application Circuits 26
  MAX32664 VERSION A FINGER-BASED HEART RATE AND SpO_2 MONITOR I^2C INTERFACE TO SENSORS 26
  MAX32664 VERSION B WRIST-BASED HEART RATE MONITOR SPI INTERFACE TO SENSORS 27
  MAX32664 VERSION C EAR-BASED HEART RATE AND SpO_2 MONITOR I^2C INTERFACE TO SENSORS . . . 28

TABLE OF CONTENTS (CONTINUED)

MAX32664 VERSION C WRIST-BASED HEART RATE AND SpO_2 MONITOR SPI INTERFACE TO SENSORS . 29
MAX32664 VERSION D FINGER-BASED HEART RATE, SpO2, BLOOD PRESSURE MONITOR I^2C INTERFACE TO SENSORS 30
Ordering Information 31
Revision History 33

LIST OF FIGURES

Figure 1. I^2C Timing Diagram 10
Figure 2. SPI Master Mode Timing Diagram 11

LIST OF TABLES

Table 1. Evaluation Platforms 26

Absolute Maximum Ratings

V_{DD} .... -0.3V to +3.63V
32KIN, 32KOUT .... -0.3V to V_{DD} + 0.3V
All Digital Pins .... -0.3V to V_{DD} + 0.3V
Total Current into All Digital Pins Combined (sink) 100mA
V_{SS} 100mA
Output Current (sink) by Any Digital Pin 25mA
Output Current (source) by Any Digital Pin .... -25mA
Continuous Package Power Dissipation 24 TQFN-EP (multilayer board) T_A = +70°C (derate 16.3mW/°C above +70°C) ... 1305mW
Operating Temperature Range .... -40°C to +105°C
Storage Temperature Range .... -65°C to +150°C
Soldering Temperature (reflow) .... +260°C

Note: All voltages with respect to V_{SS}, unless otherwise noted.

Stresses beyond those listed under "Absolute Maximum Ratings" may cause permanent damage to the device. These are stress ratings only, and functional operation of the device at these or any other conditions beyond those indicated in the operational sections of the specifications is not implied. Exposure to absolute maximum rating conditions for extended periods may affect device reliability.

Package Information

16 WLP

Package Code	W161K1+1
Outline Number	21-100241
Land Pattern Number	Refer to *Application Note 1891*
**Thermal Resistance, Four-Layer Board:**	
Junction to Ambient (θ_{JA})	66.34 °C/W
Junction to Case (θ_{JC})	N/A

24 TQFN-EP

Package Code	T2433+2C
Outline Number	21-100264
Land Pattern Number	90-100089
**Thermal Resistance, Four-Layer Board:**	
Junction to Ambient (θ_{JA})	61.3°C/W
Junction to Case (θ_{JC})	2.2°C/W

For the latest package outline information and land patterns (footprints), go to ***www.maximintegrated.com/packages***. Note that a "+", "#", or "-" in the package code indicates RoHS status only. Package drawings may show a different suffix character, but the drawing pertains to the package regardless of RoHS status.

Package thermal resistances were obtained using the method described in JEDEC specification JESD51-7, using a four-layer board. For detailed information on package thermal considerations, refer to ***www.maximintegrated.com/thermal-tutorial***.

Electrical Characteristics

(Limits are 100% tested at T_A = +25°C and T_A = +105°C. Limits over the operating temperature range and relevant supply voltage range are guaranteed by design and characterization. Specifications marked GBD are guaranteed by design and not production tested. Specifications to the minimum operating temperature are guaranteed by design and are not production tested.)

PARAMETER	SYMBOL	CONDITIONS	MIN	TYP	MAX	UNITS
**POWER SUPPLIES**						
Supply Voltage	V_{DD}		1.71	1.8	3.63	V
Power-Fail Reset Voltage	V_{RST}	Monitors V_{DD}	1.63		1.71	V
Power-On Reset Voltage	V_{POR}	Monitors V_{DD}		1.4		V

Electrical Characteristics (continued)

(Limits are 100% tested at T_A = +25°C and T_A = +105°C. Limits over the operating temperature range and relevant supply voltage range are guaranteed by design and characterization. Specifications marked GBD are guaranteed by design and not production tested. Specifications to the minimum operating temperature are guaranteed by design and are not production tested.)

PARAMETER	SYMBOL	CONDITIONS	MIN	TYP	MAX	UNITS
**DIGITAL I/O**						
Input Low Voltage for RSTN, SLAVE_SCL, SLAVE_SDA, SENSOR_SDA, SENSOR_MISO, PPG_INT, HR_INT, ACCEL_INT, MFIO	V_{IL}				0.3 × V_{DD}	V
Input High Voltage for RSTN, SLAVE_SCL, SENSOR_SDA, SENSOR_MISO, PPG_INT, ACCEL_INT, MFIO, HR_INT, SLAVE_SDA	V_{IH}		0.7 × V_{DD}			V
Output Low Voltage for SENSOR_SDA, SENSOR_SCL, SLAVE_SDA	V_{OL_I2C}	V_{DD} = 1.71V, I_{OL} = 2mA		0.2	0.4	V
Output High Voltage for SENSOR_SDA, SENSOR_SCL, SLAVE_SDA	V_{OH_I2C}	V_{DD} = 1.71V, I_{OH} = -2mA	V_{DD} - 0.4			V
Output Low Voltage for PPG_CS, ACCEL_CS, SENSOR_MOSI, SENSOR_SCK, MFIO	V_{OL}	I_{OL} = 1mA	0.4			V
Output High Voltage for PPG_CS, ACCEL_CS, SENSOR_MOSI, SENSOR_SCK, MFIO	V_{OH}	I_{OH} = -1mA	V_{DD} - 0.4			V
Input Hysteresis (Schmitt)	V_{IHYS}			300		mV
Input/Output Pin Capacitance for All Pins	C_{IO}			4		pF
Input Leakage Current Low	I_{IL}	V_{IN} = 0V	-500		+500	nA
Input Leakage Current High	I_{IH}	V_{IN} = 3.6V	-500		+500	nA
Input Pullup Resistor to RSTN	R_{PU_VDD}	Pullup to V_{DD} = 3.63V		10.5		kΩ
**CLOCKS**						
System Clock Frequency	f_{SYS_CLK}			96		MHz
RTC Input Frequency	f_{32KIN}	32.768kHz watch crystal, C_L = 6pF, ESR < 90kΩ, C_0 < 2pF		32.768		kHz
RTC Operating Current	I_{RTC}	All power modes, RTC enabled		0.45		µA

Electrical Characteristics (continued)

(Limits are 100% tested at T_A = +25°C and T_A = +105°C. Limits over the operating temperature range and relevant supply voltage range are guaranteed by design and characterization. Specifications marked GBD are guaranteed by design and not production tested. Specifications to the minimum operating temperature are guaranteed by design and are not production tested.)

PARAMETER	SYMBOL	CONDITIONS	MIN	TYP	MAX	UNITS
RTC Power-Up Time	t_{RTC_ON}			250		ms

Electrical Characteristics—I²C

(Limits are 100% tested at T_A = +25°C and T_A = +105°C. Limits over the operating temperature range and relevant supply voltage range are guaranteed by design and characterization. Specifications marked GBD are guaranteed by design and not production tested. Specifications to the minimum operating temperature are guaranteed by design and are not production tested.)

PARAMETER	SYMBOL	CONDITIONS	MIN	TYP	MAX	UNITS
**FAST MODE**						
Output Fall Time	t_{OF}	From $V_{OH(MIN)}$ to $V_{OL(MAX)}$		150		ns
Pulse Width Suppressed by Input Filter	t_{SP}			75		ns
SCL Clock Frequency	f_{SCL}		0		400	kHz
Low Period SCL Clock	t_{LOW}		1.3			µs
High Time SCL Clock	t_{HIGH}		0.6			µs
Setup Time for Repeated Start Condition	$t_{SU;STA}$		0.6			µs
Hold Time for Repeated Start Condition	$t_{HD;STA}$		0.6			µs
Data Setup Time	$t_{SU;DAT}$			125		ns
Data Hold Time	$t_{HD;DAT}$			10		ns
Rise Time for SDA and SCL	t_R			30		ns
Fall Time for SDA and SCL	t_F			30		ns
Setup Time for a Stop Condition	$t_{SU;STO}$		0.6			µs
Bus Free Time Between a Stop and Start Condition	t_{BUS}		1.3			µs
Data Valid Time	$t_{VD;DAT}$		0.9			µs
Data Valid Acknowledge Time	$t_{VD;ACK}$		0.9			µs

Electrical Characteristics—SPI

(TIming specifications are guaranteed by design and not production tested.)

PARAMETER	SYMBOL	CONDITIONS	MIN	TYP	MAX	UNITS
**MASTER MODE**						
SPI Master Operating Frequency	f_{MCK}				48	MHz
SPI Master SCK Period	t_{MCK}			$1/f_{MCK}$		ns

Electrical Characteristics—SPI (continued)

(TIming specifications are guaranteed by design and not production tested.)

PARAMETER	SYMBOL	CONDITIONS	MIN	TYP	MAX	UNITS
SCK Output Pulse-Width High/Low	t_{MCH}, t_{MCL}		$t_{MCK}/2$			ns
MOSI Output Hold Time After SCK Sample Edge	t_{MOH}		$t_{MCK}/2$			ns
MOSI Output Valid to Sample Edge	t_{MOV}		$t_{MCK}/2$			ns
MISO Input Valid to SCK Sample Edge Setup	t_{MIS}			5		ns
MISO Input to SCK Sample Edge Hold	t_{MIH}			$t_{MCK}/2$		ns

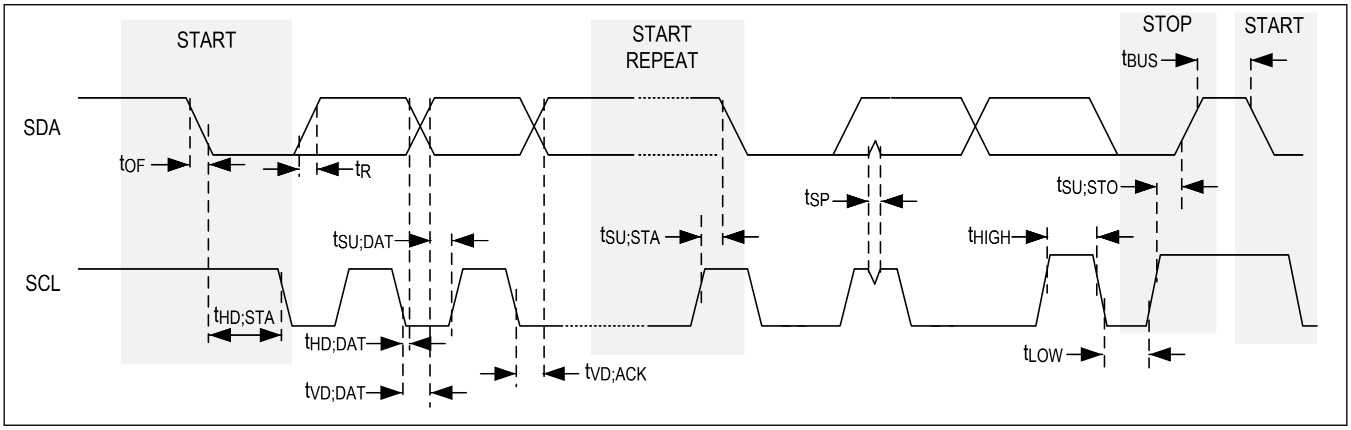

Figure 1. I²C Timing Diagram

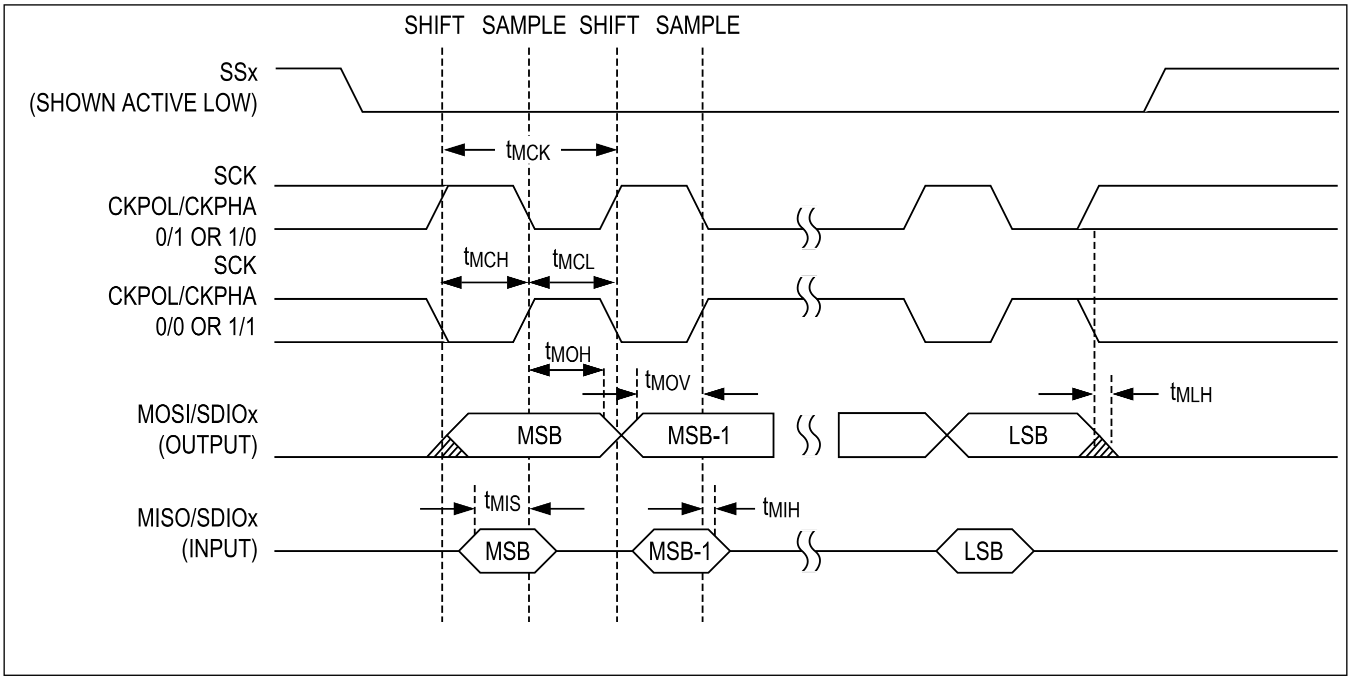

Figure 2. SPI Master Mode Timing Diagram

Pin Configuration MAX32664 Version A and Version D 16-WLP I^2C Interface to Sensors

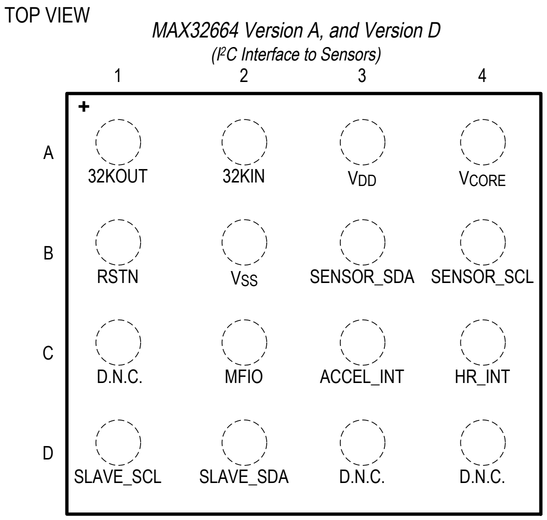

16-WLP

Pin Descriptions MAX32664 Version A and Version D 16-WLP I^2C Interface to Sensors

PIN	NAME	FUNCTION MODE	FUNCTION
		Signal Name	
**POWER**			
A3	V_{DD}	Digital Supply Voltage	This pin must be bypassed to V_{SS} with a 1.0μF capacitor as close as possible to the package. The device operates solely from this one power supply pin.
A4	V_{CORE}	Core Supply Voltage	V_{CORE} must always be bypassed to V_{SS} with a 1.0μF capacitor as close as possible to the package. Do not connect this device pin to any other circuits.
B2	V_{SS}	Digital Ground	
**CLOCK**			
A2	32KIN	32.768kHz Crystal Oscillator Input	Connect a 32.768kHz crystal between 32KIN and 32KOUT for RTC operation. Optionally, an external clock source can be driven on 32KIN if the 32KOUT pin is left unconnected.
A1	32KOUT	32.768kHz Crystal Oscillator Output	
**RESET**			
B1	RSTN	Reset	External System Reset (Active-Low) Input. The device remains in reset while this pin is in its active state. When the pin transitions to its inactive state, the device performs a reset (resetting all logic on all supplies except for real-time clock circuitry) and begins execution. This pin is internally connected with an internal pullup to the V_{DD} supply as indicated in the *Electrical Characteristics* table. Add a noise snubber circuit as close as possible to the device, with component values shown in the *Typical Application Circuits*.

PIN	NAME	FUNCTION MODE<br>Signal Name	FUNCTION
**I^2C**			
D1	SLAVE_SCL	I^2C Slave Clock	This is the I^2C slave SCL that should be connected to the host I^2C master SCL.
D2	SLAVE_SDA	I^2C Slave Data	This is the I^2C slave SDA that should be connected to the host I^2C master SDA.
B4	SENSOR_SCL	I^2C Sensor Clock	This is the I^2C master SCL that should be connected to the I^2C slave SCL on the slave sensors.
B3	SENSOR_SDA	I^2C Sensor Data	This is the I^2C master SDA that should be connected to the I^2C slave SDA on the slave sensors.
**INTERRUPTS**			
C4	HR_INT	Heart Rate/PPG Monitor Interrupt Input	This pin connects to the heart rate/PPG monitor sensor interrupt output.
C3	ACCEL_INT	Accelerometer Interrupt Input	This pin connects to the accelerometer sensor interrupt output.
C2	MFIO	Multifunction I/O	MFIO asserts low as an output when the sensor hub needs to communication with the host; MFIO acts as an input and when held low during a reset, the sensor hub enters bootloader mode.
**DO NOT CONNECT**			
C1, D3, D4	D.N.C.	Do Not Connect	This pin is internally connected. Do not make any electrical connection, including V_{SS}, to this pin.

Pin Configuration MAX32664 Version B and Version C 16-WLP SPI Interface to Sensors

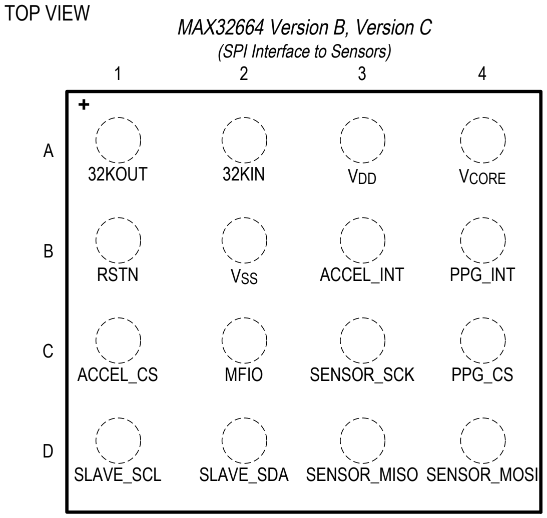

16-WLP

Pin Descriptions MAX32664 Version B and Version C 16-WLP SPI Interface to Sensors

PIN	NAME	FUNCTION MODE<br>Signal Name	FUNCTION
**POWER**			
A3	V_{DD}	Digital Supply Voltage	This pin must be bypassed to V_{SS} with a 1.0μF capacitor as close as possible to the package. The device operates solely from this one power supply pin.
A4	V_{CORE}	Core Supply Voltage	V_{CORE} must always be bypassed to V_{SS} with a 1.0μF capacitor as close as possible to the package. Do not connect this device pin to any other circuits.
B2	V_{SS}	Digital Ground	
**CLOCK**			
A2	32KIN	32.768kHz Crystal Oscillator Input	Connect a 32.768kHz crystal between 32KIN and 32KOUT for RTC operation. Optionally, an external clock source can be driven on 32KIN if the 32KOUT pin is left unconnected.
A1	32KOUT	32.768kHz Crystal Oscillator Output	
**RESET**			
B1	RSTN	Reset	External System Reset (Active-Low) Input. The device remains in reset while this pin is in its active state. When the pin transitions to its inactive state, the device performs a reset (resetting all logic on all supplies except for real-time clock circuitry) and begins execution. This pin is internally connected with an internal pullup to the V_{DD} supply as indicated in the *Electrical Characteristics* table. Add a noise snubber circuit as close as possible to the device, with component values shown in the *Typical Application Circuits*.

PIN	NAME	FUNCTION MODE	FUNCTION
		Signal Name	
**SPI**			
C3	SENSOR_SCK	SPI Master Clock for Sensors	This is the SPI master clock that should be connected to the sensor SPI SCK.
D3	SENSOR_MISO	SPI Master In Slave Out	This is the SPI master in slave out that should be connected to the sensor SPI data output pin.
D4	SENSOR_MOSI	SPI Master Out Slave In	This is the SPI master out slave in that should be connected to the sensor SPI data input pin.
C4	PPG_CS	PPG Sensor Chip Select	This is the SPI master PPG sensor chip select output that should be connected to the SPI slave PPG sensor chip select input.
C1	ACCEL_CS	Accelerometer Chip Select	This is the SPI master accelerometer chip select output that should be connected to the SPI slave accelerometer chip select input.
**I^2C**			
D1	SLAVE_SCL	I^2C Slave Clock	This is the I^2C slave SCL that should be connected to the host I^2C master SCL.
D2	SLAVE_SDA	I^2C Slave Data	This is the I^2C slave SDA that should be connected to the host I^2C master SDA.
**INTERRUPTS**			
B4	PPG_INT	PPG Sensor Interrupt Input	This pin connects to the PPG sensor interrupt output.
B3	ACCEL_INT	Accelerometer Interrupt Input	This pin connects to the accelerometer sensor interrupt output.
C2	MFIO	Multifunction I/O	The MFIO pin provides different functions. MFIO acts as an input and when held low during a reset, the sensor hub enters bootloader mode. The host asserts MFIO low when it needs to communicate with the sensor hub.

Pin Configuration MAX32664 Version A and Version D 24-TQFN I^2C Interface to Sensors

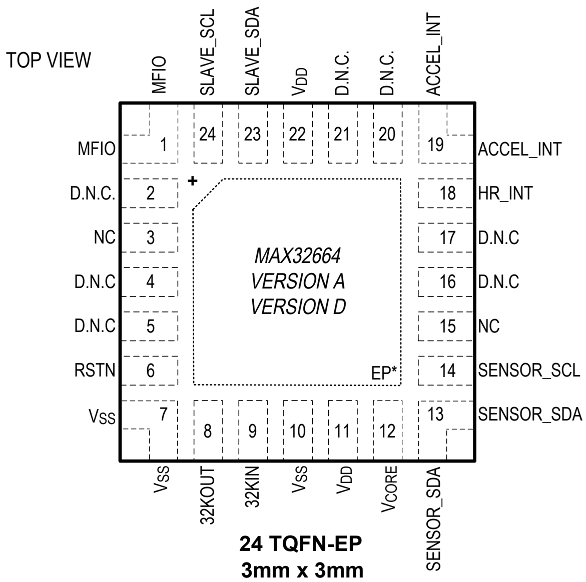

24 TQFN-EP
3mm x 3mm
I^2C Interface to Sensors
*EP = EXPOSED PAD

Pin Descriptions MAX32664 Version A and Version D 24-TQFN I^2C Interface to Sensors

PIN	NAME	FUNCTION MODE	FUNCTION
		Signal Name	
**POWER**			
11, 22	V_{DD}	Digital Supply Voltage	This pin must be bypassed to V_{SS} with a 1.0μF capacitor as close as possible to the package. The device operates solely from this one power supply pin.
12	V_{CORE}	Core Supply Voltage	V_{CORE} must always be bypassed to V_{SS} with a 1.0μF capacitor as close as possible to the package. Do not connect this device pin to any other circuits.
7, 10	V_{SS}	Digital Ground	
EP	EP	Exposed Pad	Exposed Pad (TQFN Only). This pad must be connected to V_{SS}. Refer to *Application Note 3273: Exposed Pads: A Brief Introduction* for additional information.
**CLOCK**			
9	32KIN	32.768kHz Crystal Oscillator Input	Connect a 32.768kHz crystal between 32KIN and 32KOUT for RTC operation. Optionally, an external clock source can be driven on 32KIN if the 32KOUT pin is left unconnected.
8	32KOUT	32.768kHz Crystal Oscillator Output	

PIN	NAME	FUNCTION MODE<br>Signal Name	FUNCTION
**RESET**			
6	RSTN	Reset	External System Reset (Active-Low) Input. The device remains in reset while this pin is in its active state. When the pin transitions to its inactive state, the device performs a reset (resetting all logic on all supplies except for real-time clock circuitry) and begins execution. This pin is internally connected with an internal pullup to the V_{DD} supply as indicated in the *Electrical Characteristics* table. Add a noise snubber circuit as close as possible to the device, with component values shown in the *Typical Application Circuits*.
**I^{2}C**			
24	SLAVE_SCL	I^{2}C Slave Clock	This is the I^{2}C slave SCL that should be connected to the host I^{2}C master SCL.
23	SLAVE_SDA	I^{2}C Slave Data	This is the I^{2}C slave SDA that should be connected to the host I^{2}C master SDA.
14	SENSOR_SCL	I^{2}C Sensor Clock	This is the I^{2}C master SCL that should be connected to the I^{2}C slave SCL on the slave sensors.
13	SENSOR_SDA	I^{2}C Sensor Data	This is the I^{2}C master SDA that should be connected to the I^{2}C slave SDA on the slave sensors.
**INTERRUPTS**			
18	HR_INT	Heart Rate/PPG Monitor Interrupt Input	This pin connects to the heart rate/PPG monitor sensor interrupt output.
19	ACCEL_INT	Accelerometer Interrupt Input	This pin connects to the accelerometer sensor interrupt output.
1	MFIO	Multifunction I/O	MFIO asserts low as an output when the sensor hub needs to communication with the host; MFIO acts as an input and when held low during a reset, the sensor hub enters bootloader mode.
**DO NOT CONNECT**			
2, 4, 5, 16, 17, 20, 21	D.N.C.	Do Not Connect	This pin is internally connected. Do not make any electrical connection, including V_{SS}, to this pin.
**NOT CONNECTED**			
3, 15	NC	Not Connected. This pin is not connected to the die and can be used to route any signal.	This pin is not connected to the die and can be used to route any signal.

Pin Configuration MAX32664 Version B and Version C 24-TQFN SPI Interface to Sensors

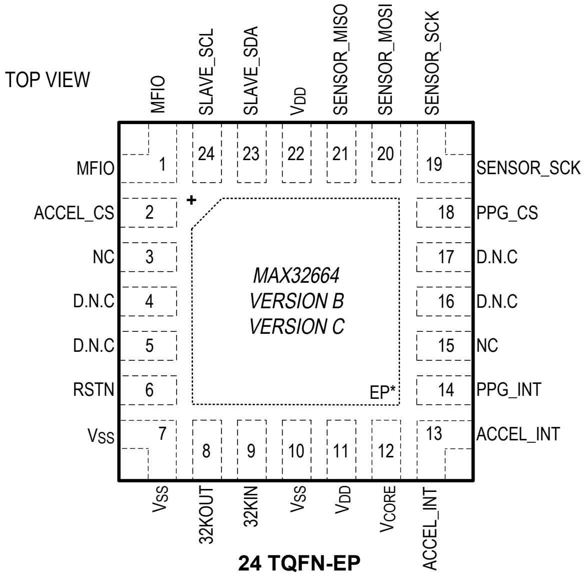

24 TQFN-EP
3mm x 3mm
SPI Interface to Sensors
*EP = EXPOSED PAD

Pin Descriptions MAX32664 Version B and Version C 24-TQFN SPI Interface to Sensors

PIN	NAME	FUNCTION MODE<br>Signal Name	FUNCTION
**POWER**			
11, 22	V_{DD}	Digital Supply Voltage	This pin must be bypassed to V_{SS} with a 1.0μF capacitor as close as possible to the package. The device operates solely from this one power supply pin.
12	V_{CORE}	Core Supply Voltage	V_{CORE} must always be bypassed to V_{SS} with a 1.0μF capacitor as close as possible to the package. Do not connect this device pin to any other circuits.
7, 10	V_{SS}	Digital Ground	
EP	EP	Exposed Pad	Exposed Pad (TQFN Only). This pad must be connected to V_{SS}. Refer to Application Note 3273: Exposed Pads: A Brief Introduction for additional information.
**CLOCK**			
9	32KIN	32.768kHz Crystal Oscillator Input	Connect a 32.768kHz crystal between 32KIN and 32KOUT for RTC operation. Optionally, an external clock source can be driven on 32KIN if the 32KOUT pin is left unconnected.
8	32KOUT	32.768kHz Crystal Oscillator Output	

PIN	NAME	FUNCTION MODE	FUNCTION
		Signal Name	
**RESET**			
6	RSTN	Reset	External System Reset (Active-Low) Input. The device remains in reset while this pin is in its active state. When the pin transitions to its inactive state, the device performs a reset (resetting all logic on all supplies except for real-time clock circuitry) and begins execution. This pin is internally connected with an internal pullup to the V_{DD} supply as indicated in the *Electrical Characteristics* table. Add a noise snubber circuit as close as possible to the device, with component values shown in the *Typical Application Circuits*.
**SPI**			
19	SENSOR_SCK	SPI Master Clock for Sensors	This is the SPI master clock that should be connected to the sensor SPI SCK.
21	SENSOR_MISO	SPI Master In Slave Out	This is the SPI master in slave out that should be connected to the sensor SPI data output pin.
20	SENSOR_MOSI	SPI Master Out Slave In	This is the SPI master out slave in that should be connected to the sensor SPI data input pin.
18	PPG_CS	PPG Sensor Chip Select	This is the SPI master PPG sensor chip select output that should be connected to the SPI slave PPG sensor chip select input.
2	ACCEL_CS	Accelerometer Chip Select	This is the SPI master accelerometer chip select output that should be connected to the SPI slave accelerometer chip select input.
**I^2C**			
24	SLAVE_SCL	I^2C Slave Clock	This is the I^2C slave SCL that should be connected to the host I^2C master SCL.
23	SLAVE_SDA	I^2C Slave Data	This is the I^2C slave SDA that should be connected to the host I^2C master SDA.
**INTERRUPTS**			
14	PPG_INT	PPG Sensor Interrupt Input	This pin connects to the PPG sensor interrupt output.
13	ACCEL_INT	Accelerometer Interrupt Input	This pin connects to the accelerometer sensor interrupt output.
1	MFIO	Multifunction I/O	This pin provides different functions. MFIO acts as an input and when held low during a reset, the sensor hub enters bootloader mode. The host asserts MFIO low when it needs to communicate with the sensor hub.
**DO NOT CONNECT**			
4, 5, 16, 17	D.N.C.	Do Not Connect	This pin is internally connected. Do not make any electrical connection, including V_{SS}, to this pin.
**NOT CONNECTED**			
3, 15	NC	Not Connected. This pin is not connected to the die and can be used to route any signal.	This pin is not connected to the die and can be used to route any signal.

Pin Configuration MAX32664 Version C 16-WLP I2C Interface to Sensors

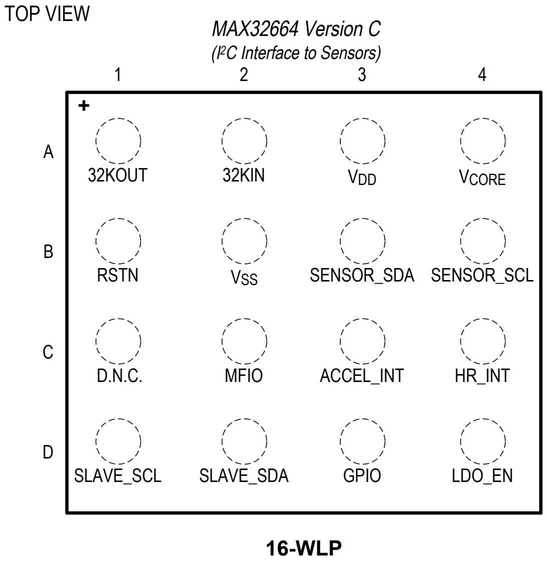

Pin Descriptions MAX32664 Version C 16-WLP I2C Interface to Sensors

PIN	NAME	FUNCTION MODE<br>Signal Name	FUNCTION
**POWER**			
A3	V_{DD}	Digital Supply Voltage	This pin must be bypassed to V_{SS} with a 1.0μF capacitor as close as possible to the package. The device operates solely from this one power supply pin.
A4	V_{CORE}	Core Supply Voltage	V_{CORE} must always be bypassed to V_{SS} with a 1.0μF capacitor as close as possible to the package. Do not connect this device pin to any other circuits.
B2	V_{SS}	Digital Ground	
**CLOCK**			
A2	32KIN	32.768kHz Crystal Oscillator Input	Connect a 32.768kHz crystal between 32KIN and 32KOUT for RTC operation. Optionally, an external clock source can be driven on 32KIN if the 32KOUT pin is left unconnected.
A1	32KOUT	32.768kHz Crystal Oscillator Output	
**RESET**			
B1	RSTN	Reset	External System Reset (Active-Low) Input. The device remains in reset while this pin is in its active state. When the pin transitions to its inactive state, the device performs a reset (resetting all logic on all supplies except for real-time clock circuitry) and begins execution. This pin is internally connected with an internal pullup to the V_{DD} supply as indicated in the *Electrical Characteristics* table. Add a noise snubber circuit as close as possible to the device, with component values shown in the *Typical Application Circuits*.
**I2C**			
D1	SLAVE_SCL	I2C Slave Clock	This is the I2C slave SCL that should be connected to the host I2C master SCL.

PIN	NAME	FUNCTION MODE	FUNCTION
		Signal Name	
D2	SLAVE_SDA	I^2C Slave Data	This is the I^2C slave SDA that should be connected to the host I^2C master SDA.
B4	SENSOR_SCL	I^2C Sensor Clock	This is the I^2C master SCL that should be connected to the I^2C slave SCL on the slave sensors.
B3	SENSOR_SDA	I^2C Sensor Data	This is the I^2C master SDA that should be connected to the I^2C slave SDA on the slave sensors.
**INTERRUPTS**			
C4	HR_INT	Heart Rate/PPG Monitor Interrupt Input	This pin connects to the heart rate/PPG monitor sensor interrupt output.
C3	ACCEL_INT	Accelerometer Interrupt Input	This pin connects to the accelerometer sensor interrupt output.
C2	MFIO	Multifunction I/O	The host asserts MFIO low when it needs to communicate with the sensor hub; MFIO acts as an input and when held low during a reset, the sensor hub enters bootloader mode.
**GPIO**			
D3	GPIO	General-Purpose Input/ Output	Open drain when programmed as output (active low). This device pin connects to the heart rate/PPG monitor sensor GPIO pin.
D4	LDO_EN	LDO Enable Output	This pin connects to the heart rate/PPG monitor sensor LDO Enable pin.
**DO NOT CONNECT**			
C1	D.N.C.	Do Not Connect	This pin is internally connected. Do not make any electrical connection, including V_{SS}, to this pin.

Pin Configuration MAX32664 Version C 24-TQFN I2C Interface to Sensors

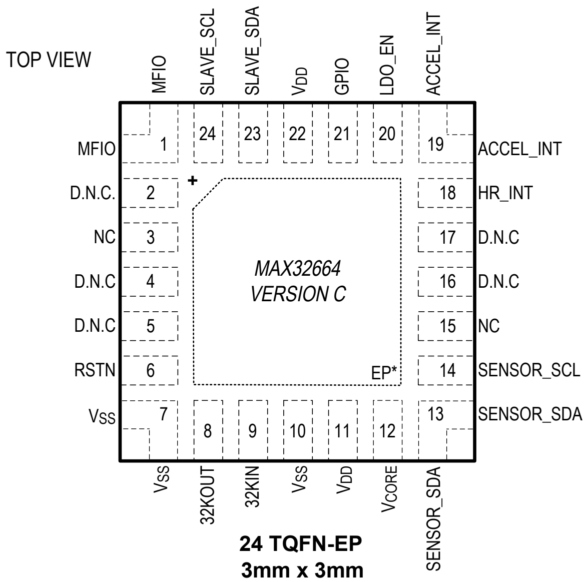

24 TQFN-EP
3mm x 3mm
I²C Interface to Sensors

*EP = EXPOSED PAD

Pin Descriptions MAX32664 Version C 24-TQFN I2C Interface to Sensors

PIN	NAME	FUNCTION MODE<br>Signal Name	FUNCTION
**POWER**			
11, 22	V_{DD}	Digital Supply Voltage	This pin must be bypassed to V_{SS} with a 1.0μF capacitor as close as possible to the package. The device operates solely from this one power supply pin.
12	V_{CORE}	Core Supply Voltage	V_{CORE} must always be bypassed to V_{SS} with a 1.0μF capacitor as close as possible to the package. Do not connect this device pin to any other circuits.
7, 10	V_{SS}	Digital Ground	
EP	EP	Exposed Pad	Exposed Pad (TQFN Only). This pad must be connected to V_{SS}. Refer to *Application Note 3273: Exposed Pads: A Brief Introduction* for additional information.
**CLOCK**			
9	32KIN	32.768kHz Crystal Oscillator Input	Connect a 32.768kHz crystal between 32KIN and 32KOUT for RTC operation. Optionally, an external clock source can be driven on 32KIN if the 32KOUT pin is left unconnected.
8	32KOUT	32.768kHz Crystal Oscillator Output	

PIN	NAME	FUNCTION MODE<br>Signal Name	FUNCTION
**RESET**			
6	RSTN	Reset	External System Reset (Active-Low) Input. The device remains in reset while this pin is in its active state. When the pin transitions to its inactive state, the device performs a reset (resetting all logic on all supplies except for real-time clock circuitry) and begins execution. This pin is internally connected with an internal pullup to the V_{DD} supply as indicated in the *Electrical Characteristics* table. Add a noise snubber circuit as close as possible to the device, with component values shown in the *Typical Application Circuits*.
**I^2C**			
24	SLAVE_SCL	I^2C Slave Clock	This is the I^2C slave SCL that should be connected to the host I^2C master SCL.
23	SLAVE_SDA	I^2C Slave Data	This is the I^2C slave SDA that should be connected to the host I^2C master SDA.
14	SENSOR_SCL	I^2C Sensor Clock	This is the I^2C master SCL that should be connected to the I^2C slave SCL on the slave sensors.
13	SENSOR_SDA	I^2C Sensor Data	This is the I^2C master SDA that should be connected to the I^2C slave SDA on the slave sensors.
**INTERRUPTS**			
18	HR_INT	Heart Rate/PPG Monitor Interrupt Input	This pin connects to the heart rate/PPG monitor sensor interrupt output.
19	ACCEL_INT	Accelerometer Interrupt Input	This pin connects to the accelerometer sensor interrupt output.
1	MFIO	Multifunction I/O	The host asserts MFIO low when it needs to communicate with the sensor hub; MFIO acts as an input and when held low during a reset, the sensor hub enters bootloader mode.
**GPIO**			
21	GPIO	General-Purpose Input/ Output	Open drain when programmed as output (active low). This device pin connects to the heart rate/PPG monitor sensor GPIO pin.
20	LDO_EN	LDO Enable Output	This pin connects to the heart rate/PPG monitor sensor LDO Enable pin.
**DO NOT CONNECT**			
2, 4, 5, 16, 17	D.N.C.	Do Not Connect	This pin is internally connected. Do not make any electrical connection, including V_{SS}, to this pin.
**NOT CONNECTED**			
3, 15	NC	Not Connected. This pin is not connected to the die and can be used to route any signal.	This pin is not connected to the die and can be used to route any signal.

Detailed Description

The MAX32664 is a sensor hub family with embedded firmware and algorithms for wearables. It seamlessly enables customer-desired sensor functionality, including communication with Analog Devices' optical sensor solutions and delivering raw or calculated data to the outside world. This is achieved while keeping overall system power consumption in check. The device family interfaces to a microcontroller host through a fast-mode slave I^2C interface for access to raw and processed sensor data as well as field updates. The sampling is adjusted automatically by the algorithm to minimize power consumption and can be configured by the user as needed.

Finger Heart Rate, SpO_2 Algorithm (Version A)

The MAX32664 Version A communicates with the MAX30101/MAX30102 through I^2C to perform finger-based heart rate and blood oxygen saturation (SpO_2) monitoring. The embedded algorithm uses digital filtering, pressure/position compensation, advanced R-wave detection, and automatic gain control to determine the heart rate in beats per minute while minimizing power. Also, the Analog Devices sensor hardware has built-in ambient light rejection to minimize background noise. SpO_2 results are reported as percentage of hemoglobin that is saturated with oxygen. The calibration values for SpO_2 configuration should be determined prior to deploying the end product. Use of an accelerometer is recommended to detect and compensate for the artifacts of motion on the algorithm.

Wrist Heart Rate Algorithm (Version B)

The MAX32664 Version B communicates with the MAX86140/MAX86141 through SPI to perform wrist-based heart rate measurements. The embedded algorithm uses digital filtering, distance/motion compensation, and advanced R-wave detection to determine the pulse rate in beats per minute. Power usage is minimized with automatic gain control. In addition, the Analog Devices sensor hardware provides additional features such as ambient light rejection, high signal-to-noise (SNR) ratio, and external LEDs for optimal placement. Use of an accelerometer is required to detect and compensate for the artifacts of motion on the algorithm.

Wrist or Ear Heart Rate, SpO_2 Algorithm (Version C)

The MAX32664 Version C communicates with the MAX86141 through SPI or to the MAXM86161 through I^2C to perform wrist-based or ear-based heart rate and blood oxygen saturation (SpO_2) measurements. The embedded algorithm uses digital filtering, distance/motion compensation, and advanced R-wave detection to determine the pulse rate in beats per minute. Power usage is minimized with automatic gain control. SpO_2 results are reported as a percentage of hemoglobin that is saturated with oxygen. The calibration values for SpO_2 configuration should be determined before deploying the end product. In addition, the Analog Devices sensor hardware provides additional features such as ambient light rejection, high signal-to-noise (SNR) ratio, and external LEDs for optimal placement. Use of an accelerometer is required to detect and compensate for the artifacts of motion on the algorithm.

Finger Heart Rate, SpO_2, Blood Pressure Algorithm (Version D)

The MAX32664 Version D communicates with the MAX30101/MAX30102 through I^2C to perform finger-based heart rate, blood oxygen saturation (SpO_2), and blood pressure monitoring. The embedded algorithm uses digital filtering, pressure/position compensation, advanced R-wave detection, and automatic gain control to determine the heart rate in beats per minute while minimizing power. Also, the Analog Devices sensor hardware has built-in ambient light rejection to minimize background noise. SpO_2 results are reported as percentage of hemoglobin that is saturated with oxygen. The calibration values for SpO_2 configuration should be determined before deploying the end product. Estimated blood pressure is reported for systolic and diastolic blood pressure. Blood pressure cuff measurements are used to set the blood pressure calibration data in the firmware. An accelerator is not required by the algorithm.

Algorithm Selection and Evaluation

During early stages of algorithm selection and evaluation, a user may wish to experiment with different versions of the MAX32664. To facilitate this, the MAX32664 Version Z was created. The MAX32664 Version Z contains only the bootloader and a Z encryption key. The Version Z encryption key is used as a generic key that allows multiple, Analog Devices-supplied algorithms to be accepted by the bootloader (not at the same time). For example, various Analog

Devices evaluation kits use the MAX32664 Version Z. This allows any of the versions of algorithm to be evaluated. In order to do this, a special version of the algorithms must be obtained from Analog Devices (Version A, B, C, or D algorithm with Z encryption key). Furthermore, it must be noted that the sensor interface/device pinout may be different between various algorithm versions, such as I^2C vs. SPI. The MAX32664 Version Z is not intended for mass production.

Each production version (A, B, C, or D) has its own bootloader with encryption and an initial preprogrammed version of the firmware. If the user determines that performance of the initial preprogrammed firmware is not sufficient for their application, they can upgrade the firmware by going to the MAX32664 webpage *MAX32664 Ultra-Low Power Biometric Sensor Hub* to obtain the latest version of the firmware.

Interface to Host

The interface to the host is the I^2C interface. The devices support one slave interface with the 7-bit address of 0x55. The MAX32664B and the MAX32664C provide a selectable address feature that can be accessed by the host through an I^2C command. The following features for this interface are as follows:

- One slave for communication with a host
- RESTART condition
- Fast mode: 400kbps
- Internal filter to reject noise spikes
- Receiver FIFO depth of 8 bytes
- Transmitter FIFO depth of 8 bytes
- Firmware bootloader

Interface to the Sensors

The interface to the sensors is either a master SPI or a fast-mode I^2C. Pullup resistors are required for the I^2C.

Device Selection

See the *Ordering Information* table at the end of this data sheet for device selection by interface type.

Applications Information

Evaluation Platforms

The actual device (IC) used in the evaluation platforms may be the MAX32664 Version Z preprogrammed with the corresponding algorithm. See the *Algorithm Selection and Evaluation* section for additional information.

Table 1. Evaluation Platforms

DEVICE	PLATFORM
MAX32664 Version A and Version D	MAXREFDES220#
MAX32664 Version B	MAXREFDES101#
MAX32664 Version C	MAXREFDES103#

Typical Application Circuits

MAX32664 VERSION A FINGER-BASED HEART RATE AND SpO_2 MONITOR I^2C INTERFACE TO SENSORS

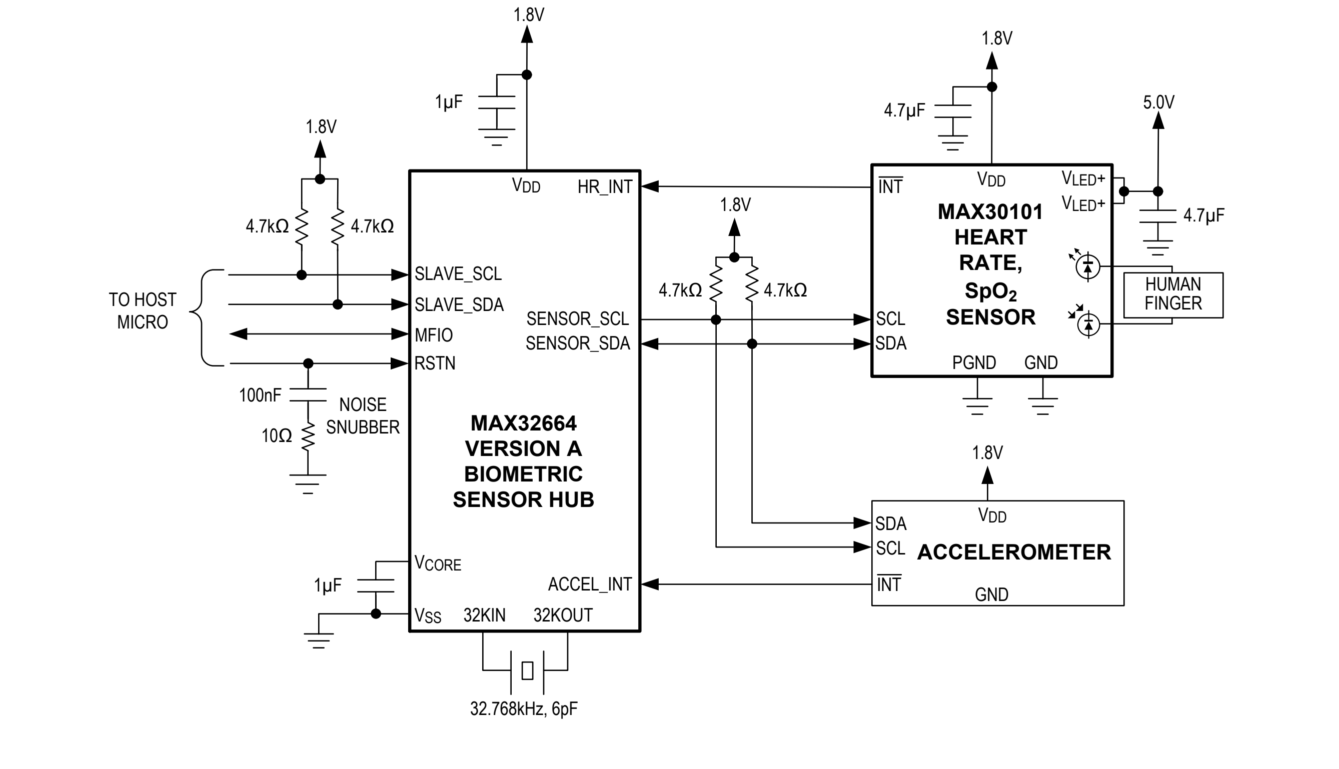

Typical Application Circuits (continued)

MAX32664 VERSION B WRIST-BASED HEART RATE MONITOR SPI INTERFACE TO SENSORS

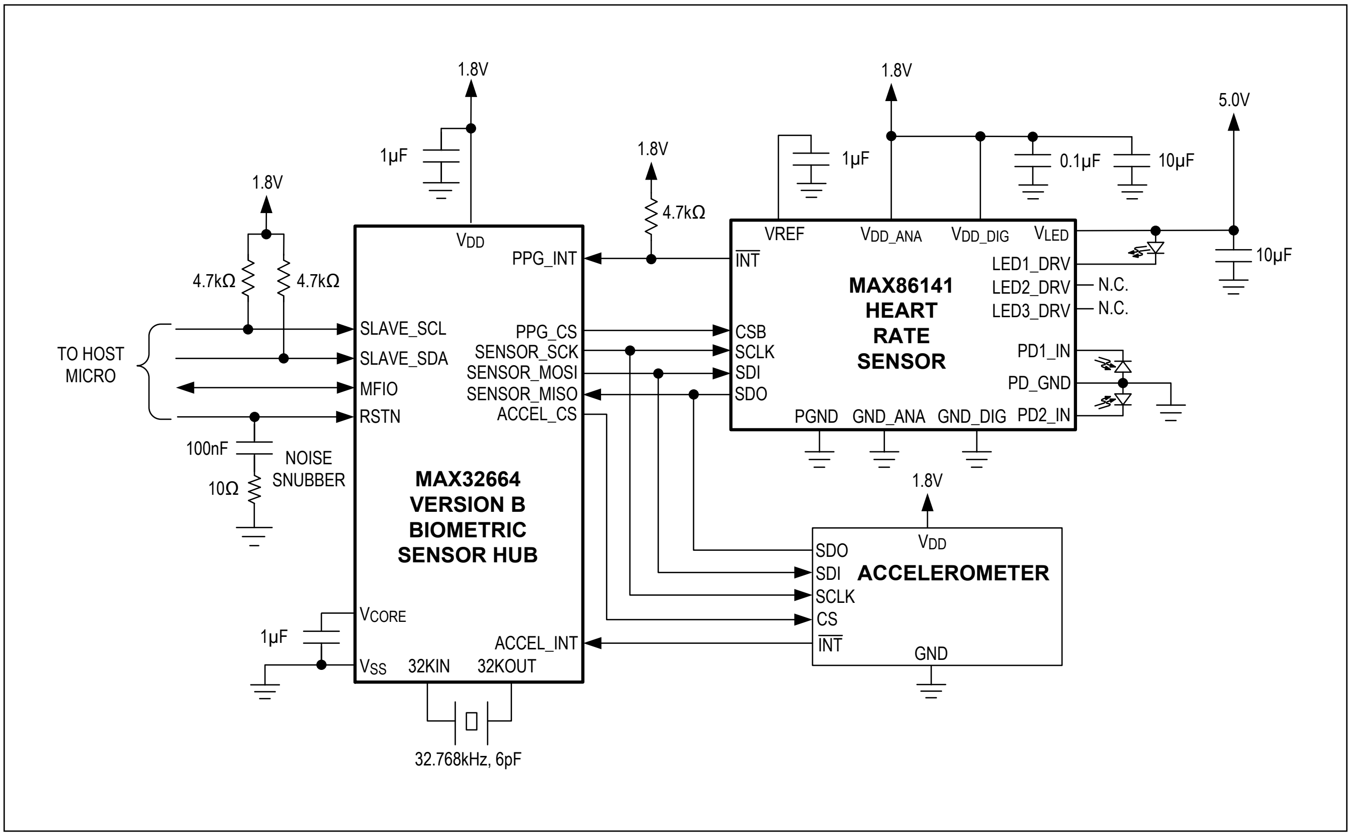

Typical Application Circuits (continued)

MAX32664 VERSION C EAR-BASED HEART RATE AND SpO_2 MONITOR I^2C INTERFACE TO SENSORS

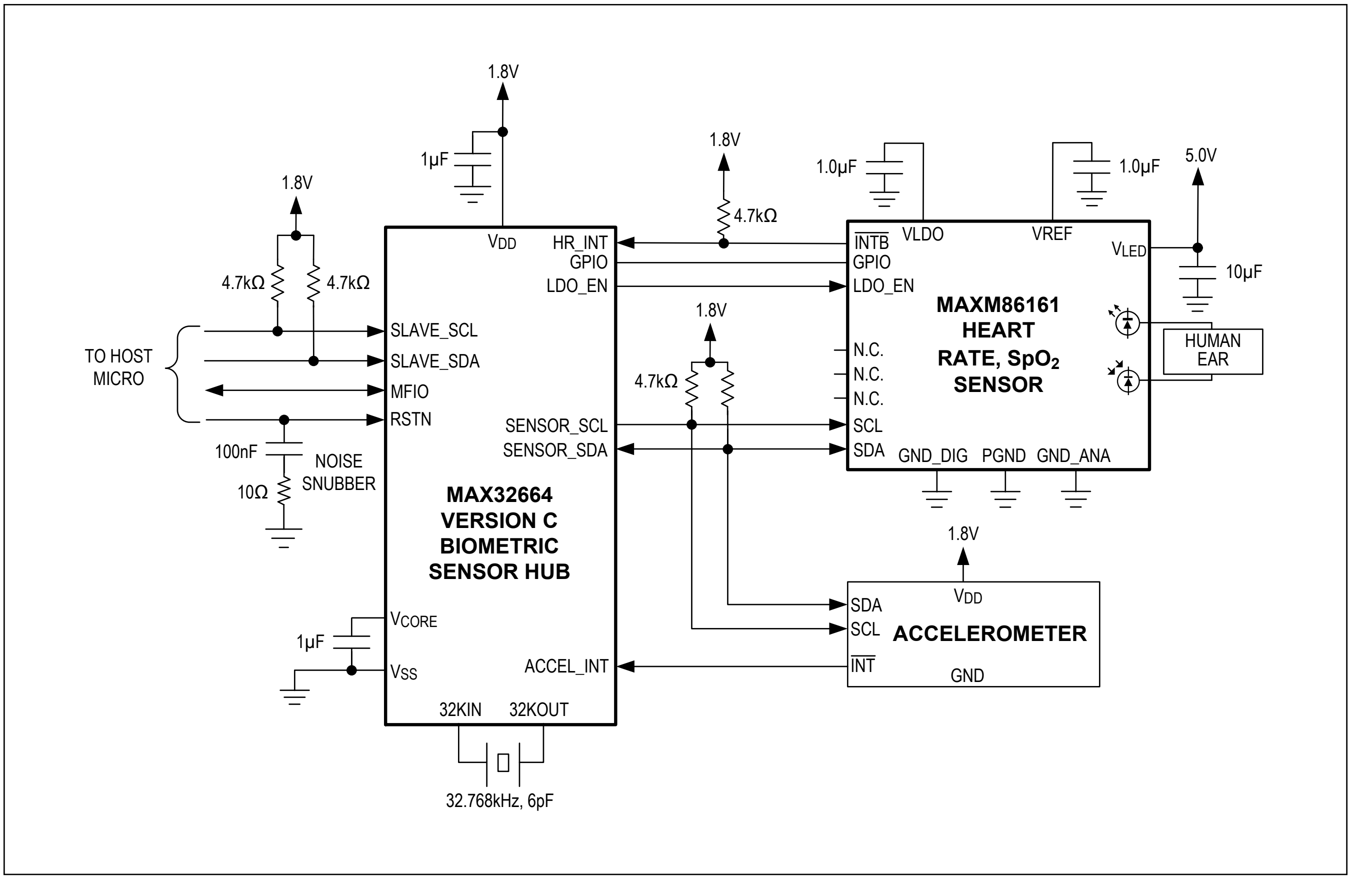

Typical Application Circuits (continued)

MAX32664 VERSION C WRIST-BASED HEART RATE AND SpO_2 MONITOR SPI INTERFACE TO SENSORS

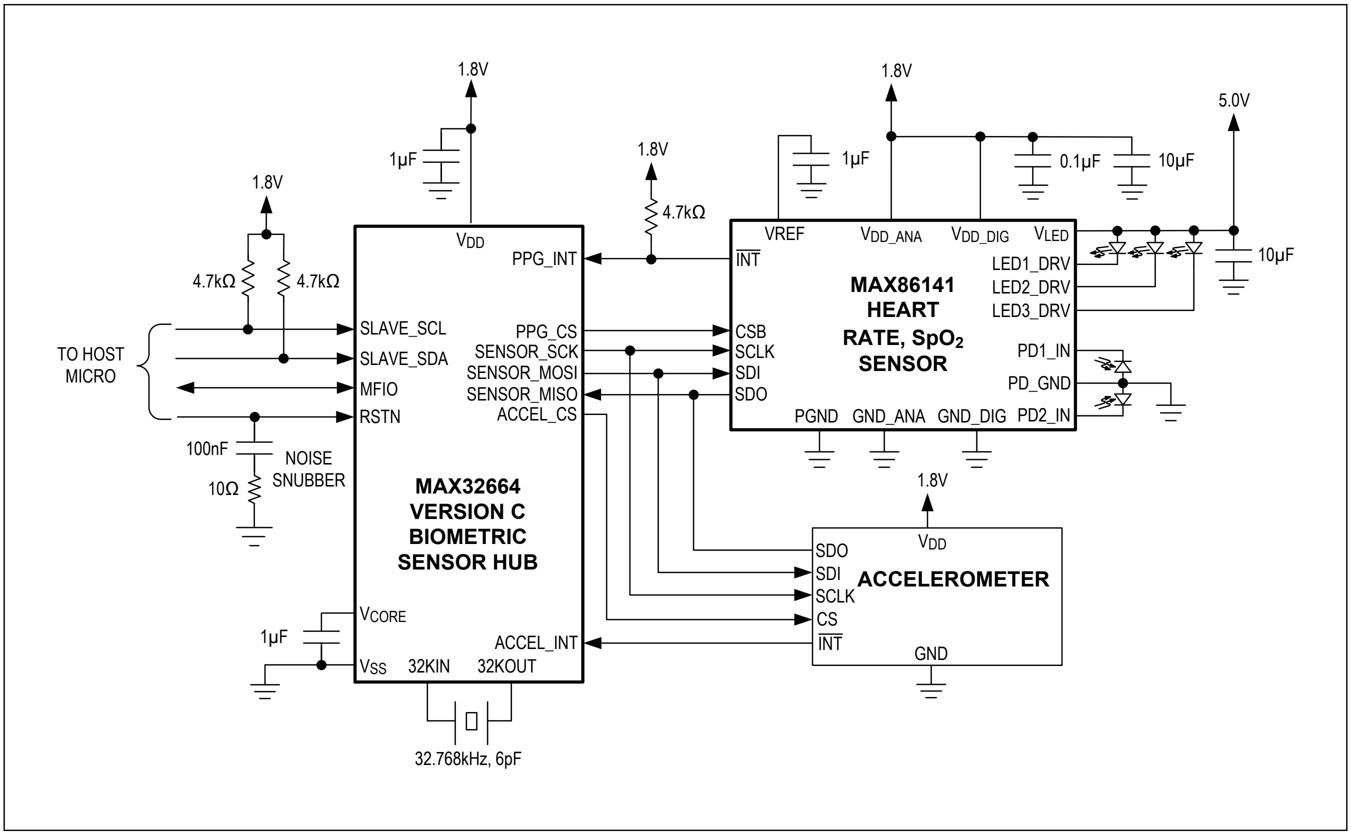

Typical Application Circuits (continued)

MAX32664 VERSION D FINGER-BASED HEART RATE, SpO2, BLOOD PRESSURE MONITOR I2C INTERFACE TO SENSORS

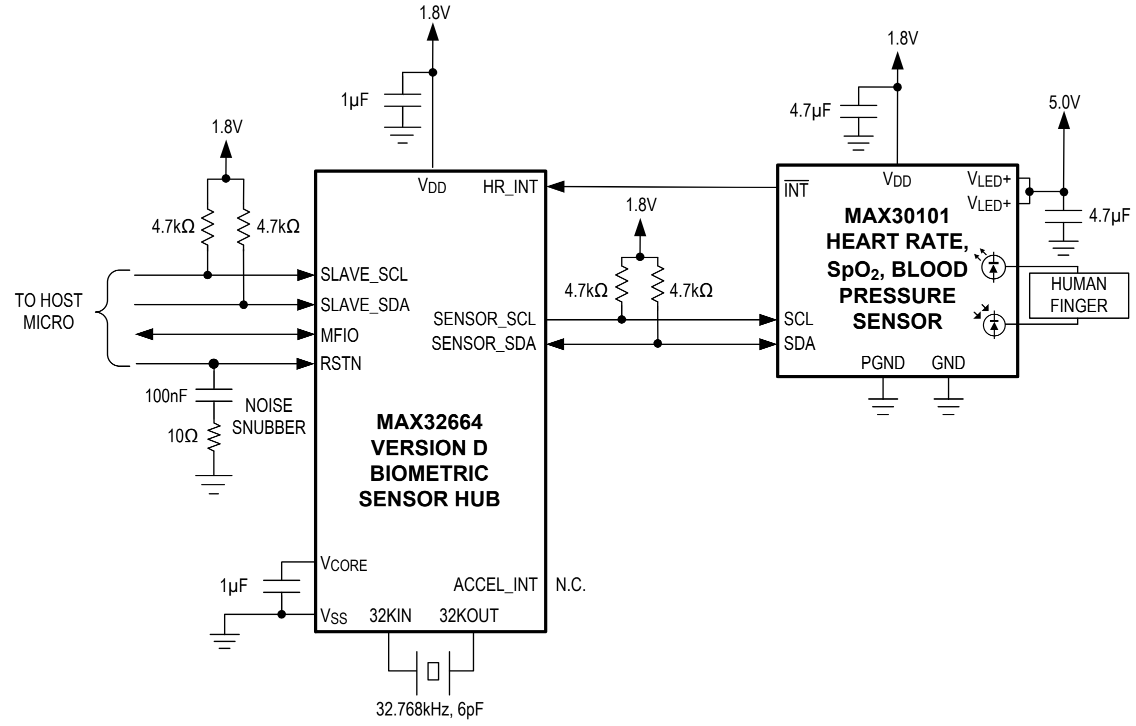

Ordering Information

PART	VERSION	SENSOR INTERFACE	COMPATIBLE SENSORS	PIN-PACKAGE
MAX32664GWEA+	A	I^2C	MAX30101	16 WLP (1.6mm x 1.6mm x 0.65mm, 0.35mm pitch)
MAX32664GWEA+T	A	I^2C	MAX30101	16 WLP (1.6mm x 1.6mm x 0.65mm, 0.35mm pitch)
MAX32664GWEB+	B	SPI	MAX86140/141	16 WLP (1.6mm x 1.6mm x 0.65mm, 0.35mm pitch)
MAX32664GWEB+T	B	SPI	MAX86140/141	16 WLP (1.6mm x 1.6mm x 0.65mm, 0.35mm pitch)
MAX32664GWEC+	C	SPI/I^2C	MAX86140/141, MAXM86161	16 WLP (1.6mm x 1.6mm x 0.65mm, 0.35mm pitch)
MAX32664GWEC+T	C	SPI/I^2C	MAX86140/141, MAXM86161	16 WLP (1.6mm x 1.6mm x 0.65mm, 0.35mm pitch)
MAX32664GWED+	D	I^2C	MAX30101/102	16 WLP (1.6mm x 1.6mm x 0.65mm, 0.35mm pitch)
MAX32664GWED+T	D	I^2C	MAX30101/102	16 WLP (1.6mm x 1.6mm x 0.65mm, 0.35mm pitch)
MAX32664GTGA+	A	I^2C	MAX30101/102	24 TQFN-EP (3mm x 3mm x 0.75mm, 0.4mm pitch)
MAX32664GTGA+T	A	I^2C	MAX30101/102	24 TQFN-EP (3mm x 3mm x 0.75mm, 0.4mm pitch)
MAX32664GTGB+	B	SPI	MAX86140/141	24 TQFN-EP (3mm x 3mm x 0.75mm, 0.4mm pitch)
MAX32664GTGB+T	B	SPI	MAX86140/141	24 TQFN-EP (3mm x 3mm x 0.75mm, 0.4mm pitch)
MAX32664GTGC+	C	SPI/I^2C	MAX86140/141, MAXM86161	24 TQFN-EP (3mm x 3mm x 0.75mm, 0.4mm pitch)
MAX32664GTGC+T	C	SPI/I^2C	MAX86140/141, MAXM86161	24 TQFN-EP (3mm x 3mm x 0.75mm, 0.4mm pitch)
MAX32664GTGD+	D	I^2C	MAX30101/102	24 TQFN-EP (3mm x 3mm x 0.75mm, 0.4mm pitch)
MAX32664GTGD+T	D	I^2C	MAX30101/102	24 TQFN-EP (3mm x 3mm x 0.75mm, 0.4mm pitch)

MAX32664GWEZ+	Z	—	—	16 WLP<br>(1.6mm x 1.6mm x 0.65mm,<br>0.35mm pitch)
MAX32664GTGZ+	Z	—	—	24 TQFN-EP<br>(3mm x 3mm x 0.75mm, 0.4mm<br>pitch)

+Denotes a lead(Pb)-free/RoHS-compliant package.

T = Tape and reel. Full reel.

Revision History

REVISION NUMBER	REVISION DATE	DESCRIPTION	PAGES CHANGED
0	4/18	Initial release	—
1	2/19	Revised entire data sheet	1–13
2	5/19	Updated *General Description*, *Benefits and Features*, *Simplified Block Diagram*, *Pin Configurations*, *Pin Descriptions*, *Wrist Heart Rate SpO2 Algorithm (Version C)*, *Finger Heart Rate SpO2 Blood Pressure Algorithm (Version D)*, *Algorithm Selection and Evaluation*, *Typical Application Circuits*, and *Ordering Information* sections	1, 2, 7–10, 14–16
3	4/20	Updated *General Description*, *Benefits and Features*, *Absolute Maximum Ratings*, *Electrical Characteristics*, *Electrical Characteristics—I^2C*, *Pin Description*, *Finger Heart Rate, SpO_2 (Version A)*, *Wrist Heart Rate (Version B)*, *Wrist or Ear Heart Rate, SpO_2 Algorithm (Version C)*, *Finger Heart Rate, SpO_2, Blood Pressure Algorithm (Version D)*, *Interface to Host*, and *Ordering Information*	1, 7–9, 12–21, 27, 28
4	2/22	Added HR_INT and SLAVE_SDA to VIL Electrical Characteristics. Added VOL Electrical Characteristics. Updated the link to the MAX32664 Ultra-Low Power Biometric Sensor Hub webpage located in the *Algorithm Selection and Evaluation* section. Updated the *Interface to Host* description. Added new Pin Configuration and Pin Descriptions for Version C I^2C to Sensors. Updated MAX32664 VERSION C EAR-BASED HEART RATE AND SpO_2 MONITOR I^2C INTERFACE TO SENSORS Typical Application Circuit. Updated package measurements in Ordering Information table.	8, 9, 12–14, 16, 17, 19–23, 25, 28, 31, 32

ANALOG DEVICES
www.analog.com

Information furnished by Analog Devices is believed to be accurate and reliable. However, no responsibility is assumed by Analog Devices for its use, nor for any infringements of patents or other rights of third parties that may result from its use. Specifications subject to change without notice. No license is granted by implication or otherwise under any patent or patent rights of Analog Devices. Trademarks and registered trademarks are the property of their respective owners.
