# Supplementary material for: Validation of an mHealth System for Monitoring Fundamental Physiological Parameters in the Clinical Setting
Source: Sensors (Basel). 2024 Aug 10;24(16):5164. doi: 10.3390/s24165164 (PMC11359666; doi:10.3390/s24165164)
Supplement: Supplementary file 1 [file sensors-24-05164-s001.zip › MLX90615-Datasheet-Melexis-953269.pdf]

MLX90615

Infra Red Thermometer

Features and Benefits

- Small size, low cost
- Easy to integrate
- Factory calibrated in wide temperature range:
  -40…85˚C for sensor temperature and
  -40…115˚C for object temperature
- High accuracy of 0.5℃ over wide temperature range (0...+50℃ for both T_A and T_O)
- High (medical) accuracy calibration
- Measurement resolution of 0.02℃
- SMBus compatible digital interface
- Power saving mode
- Customizable PWM output for continuous reading
- Embedded emissivity compensation
- 3V supply voltage

Applications Examples

- High precision non-contact temperature measurements
- Hand-held thermometers
- Ear thermometers
- Home appliances with temperature control
- Healthcare
- Livestock monitoring
- Multiple zone temperature control – up to 127 sensors can be read via common 2 wires

Ordering Information

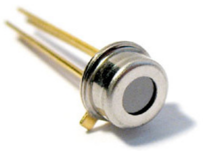

Part No.	Temperature Code	Package Code	- Option Code	Standard part	Packing form
			- X X X		
MLX90615	S (-20℃...85℃)	SG (TO-46)	(1) (2) (3)	-000	-TU

(1) Accuracy
D – medical accuracy

(2) Specifics:
A – straight pins for thru hole mounting

(3) Package options:
A – 100° FOV
G – 80° FOV

Example:
MLX90615SSG-DAA-000-TU

1 Functional diagram

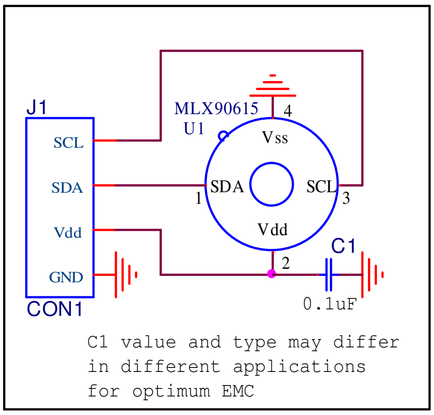

Figure 1 Typical application schematics – MLX90615 connection to SMBus

2 General description

The MLX90615 is an Infra Red thermometer for non contact temperature measurements. Both the IR sensitive thermopile detector chip and the signal conditioning chip are integrated in the same TO-46 can package.

Thanks to its low noise amplifier, 16-bit ADC and powerful DSP unit, a high accuracy and resolution of the thermometer is achieved.

The thermometer is factory calibrated with the digital SMBus compatible interface enabled. Readout resolution is 0.02℃.

General description (continued)

The MLX90615 is built from 2 chips, the Infra Red thermopile detector and the signal conditioning chip MLX90325, specially designed by Melexis to process the output of IR sensor.
The device is available in an industry standard TO-46 package.

Thanks to the low noise amplifier, high resolution 16-bit ADC and powerful DSP unit of the MLX90325, Melexis is able to deliver a high accuracy and high resolution infrared thermometer. The calculated object and ambient temperatures are available in the RAM memory of the MLX90325 with a resolution of 0.02˚C. The values are accessible by 2 wire serial SMBus compatible protocol with a resolution of 0.02℃ or via a 10-bit PWM (Pulse Width Modulated) signal from the device.

The MLX90615 is factory calibrated in standard temperature ranges from: -40...85˚C for the ambient temperature and from -40...115˚C for the object temperature.

As a standard, the MLX90615 is delivered with a programmed object emissivity of 1. It can be easily customized by the customer for any other emissivity in the range 0.1...1.0 without the need of recalibration with a black body.

The MLX90615 can be battery powered.

An optical filter (5.5µm …14µm long-wave pass) that cuts off the visible and near infra-red radiant flux is integrated in the package to make the sensor insensitive to visible light.

3 Table of Contents

1 Functional diagram 1
2 General description..... 1
3 Table of Contents 3
4 Glossary of Terms 4
5 Maximum ratings 4
6 Pin definitions and descriptions..... 5
7 Electrical Specification..... 6
8 Detailed description 8
8.1 Block diagram 8
8.2 Signal processing principle 8
8.3 Block description 8
8.3.1 Amplifier..... 8
8.3.2 Power-On-Reset (POR) 9
8.3.3 EEPROM 9
8.3.4 RAM..... 10
8.4 SMBus compatible 2-wire protocol 11
8.4.1 Functional description 11
8.4.2 Differences with the standard SMBus specification (reference [1]) 11
8.4.3 Detailed description..... 11
8.4.4 Bit transfer 13
8.4.5 Commands 13
8.4.6 SMBus Communication examples..... 14
8.4.7 Timing specification..... 14
8.4.8 Sleep Mode..... 15
8.5 Switching between PWM and SMBus..... 16
8.5.1 PWM to SMBus mode..... 16
8.5.2 SMBus to PWM mode..... 16
8.6 PWM 16
8.6.1 PWM format..... 17
8.6.2 Customizing the temperature range for PWM output..... 17
8.7 Principle of operation..... 19
8.7.1 Ambient temperature T_A..... 19
8.7.2 Object temperature T_O 19
9 Performance Graphs 20
9.1 Temperature accuracy of the MLX90615..... 20
9.2 Field Of View (FOV) 21
10 Applications Information..... 23
10.1 Use of the MLX90615 thermometer in SMBus configuration..... 23
10.2 Use of multiple MLX90615s in SMBus configuration..... 23
10.3 PWM output 24
11 Application Comments..... 25
12 Standard information regarding manufacturability of Melexis products with different soldering processes..... 26
13 ESD Precautions 27
14 FAQ..... 27
15 Package Information..... 29
16 Part marking 29
17 Table of figures..... 30
18 References 31
19 Disclaimer..... 31

4 Glossary of Terms

PTAT	**P**roportional **T**o **A**bsolute **T**emperature sensor (package temperature)
POR	**P**ower **O**n **R**eset
HFO	**H**igh **F**requency **O**scillator (RC)
DSP	**D**igital **S**ignal **P**rocessing
FIR	**F**inite **I**mpulse **R**esponse. Digital filter
IIR	**I**nfinite **I**mpulse **R**esponse. Digital filter
IR	**I**nfra-**R**ed
DC	**D**irect **C**urrent (for settled conditions specifications)
LPF	**L**ow **P**ass **F**ilter
FOV	**F**ield **O**f **V**iew
SDA, SCL	**S**erial **DA**ta, **S**erial **CL**ock – SMBus compatible communication pins
T_A	**A**mbient **T**emperature measured from the chip – (the package temperature)
T_O	**O**bject **T**emperature, 'seen' from IR sensor
ESD	**E**lectro-**S**tatic **D**ischarge
EMC	**E**lectro-**M**agnetic **C**ompatibility
TBD	**T**o **B**e **D**efined

Table 1: Glossary of Terms

5 Maximum ratings

Parameter	MLX90615
Supply Voltage, V_{DD} (over voltage)	5V
Supply Voltage, V_{DD} (operating)	3.6V
Reverse Voltage	0.5 V
Operating Temperature Range, T_A	-20…+85℃
Storage Temperature Range, T_S	-20…+125℃
ESD Sensitivity (AEC Q100 002)	2kV
DC sink current, SDA pin	25 mA
DC clamp current, SDA pin	10 mA
DC clamp current, SCL pin	10 mA

Table 2: Absolute maximum ratings for MLX90615

Exceeding the absolute maximum ratings may cause permanent damage. Exposure to absolute maximum rated conditions for extended periods may affect device reliability.

6 Pin definitions and descriptions

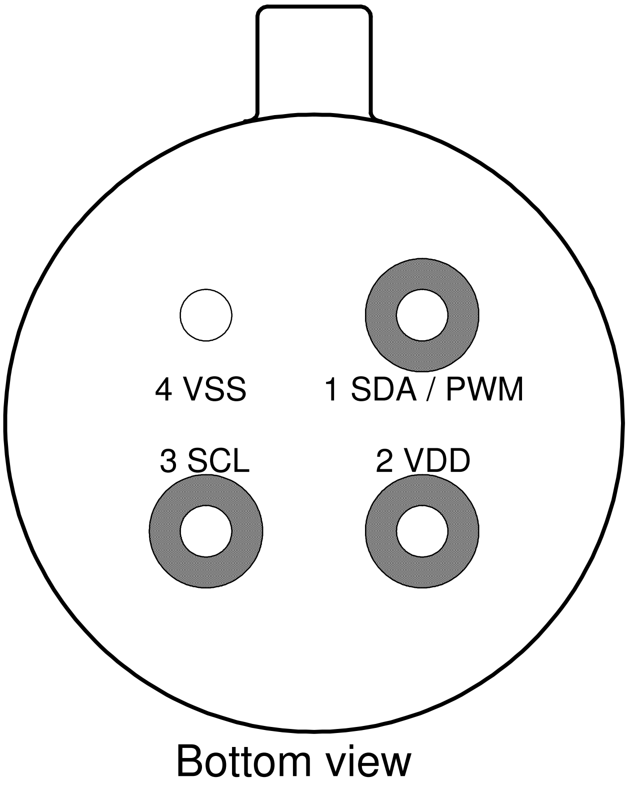

Figure 2: Pin description MLX90615

Pin Name	Function
**SDA / PWM**	Digital input / output open drain NMOS. In SMBus mode (factory default) Serial Data I/O. In PWM mode – PWM output. Weak pull-up (300kΩ typ) is present on this pin.
**VDD**	External supply voltage.
**SCL**	Serial clock input for 2 wire communications protocol. Weak pull-up (300kΩ typ) is present on this pin.
**VSS**	Ground. The metal can is also connected to this pin.

Table 3: Pin description MLX90615

Notes:
For EMC and isothermal conditions reasons, it is highly recommended not to use any electrical connection to the metal can except by the Vss pin.

Both SCL and SDA pin have input Schmidt trigger when the thermometer is operated in the 2-wire SMBus interface mode.

7 Electrical Specification

All parameters are valid for T_A = 25 ˚C, V_{DD} =3V (unless otherwise specified)

Parameter	Symbol	Test Conditions	Min	Typ	Max	Units
**Supplies**						
External supply	V_{DD}		2.6	3	3.4	V
Supply current	I_{DD}	No load		1.3	1.5	mA
Supply current (programming)	I_{DDpr}	No load, erase / write EEPROM operations		1.5		mA
Power-down supply current	Isleep	No load, SCL and SDA high		1.1	3	µA
**Power On Reset**						
POR level	V_{POR}	Power-up, power-down and brown-out	0.8	1.5	1.9	V
V_{DD} rise time	T_{POR}	Ensure POR signal			20	ms
Output valid	Tvalid	After POR		0.5		s
**EEPROM**						
Data retention		T_A = +85℃	10			years
Erase/write cycles		T_A = +25℃	100,000			Times
Erase/write cycles		T_A = +85℃	40,000			Times
Erase cell time	Terase			5		ms
Write cell time	Twrite			5		ms
**Pulse width modulation**						
PWM resolution	PWMres	Data band		10		bit
PWM output period	$PWM_{T,H,def}$	Factory default high frequency PWM, HFO factory calibrated		1.024		ms
PWM output period	$PWM_{T,L}$	Low frequency PWM, HFO factory calibrated		102.4		ms
PWM period stability	$dPWM_T$	Internal oscillator factory calibrated, over the entire operation range and supply voltage	-15		+15	%
Output low Level	PWM_{LO}	I_{sink} = 2 mA			VSS+0.2	V
Output sink current	$Isink_{PWM}$	Vout,L = 0.5V		10		mA

Table 4 Electrical specification parameters of the MLX90615

Parameter	Symbol	Test Conditions	Min	Typ	Max	Units
SMBus compatible 2-wire interface*						
Input high voltage	$V_{IH}(T_A,V)$	Over temperature and supply	VDD-0.1			V
Input low voltage	$V_{IL}(T_A,V)$	Over temperature and supply			0.6	V
Output low voltage	V_{OL}	Over temperature and supply, Isink = 2mA			0.2	V
SCL,SDA leakage	I_{leak}	$V_{SCL}=V_{DD}$, $V_{SDA}=V_{DD}$, T_A =+85°C			0.25	μA
SCL capacitance	C_{SCL}				10	pF
SDA capacitance	C_{SDA}				10	pF
Slave address	SA	Factory default		**5B**		hex
WakeUp Request	t_{wake}	SCL low	21		39	ms
SMBus Request	t_{REQ}	SCL low	39			ms
Timeout, low	$T_{imeout,L}$	SCL low	21		39	ms
Timeout, high	$T_{imeout,H}$	SCL high	52		78	μs
Acknowledge setup time	Tsuac(MD)	8-th SCL falling edge, Master			1.5	μs
Acknowledge hold time	Thdac(MD)	9-th SCL falling edge, Master			1.5	μs
Acknowledge setup time	Tsuac(SD)	8-th SCL falling edge, Slave			2.5	μs
Acknowledge hold time	Thdac(SD)	9-th SCL falling edge, Slave			1.5	μs

Table 5 Electrical specification parameters of the MLX90615 (continued)

Notes: All the communication and refresh rate timings are given for the nominal calibrated HFO frequency and will vary with this frequency's variations.

**SMBus compatible interface is described in details in the SMBus detailed description section. Maximum number of MLX90615 devices on one bus is 127, higher pull-up currents are recommended for higher number of devices, faster bus data transfer rates, and increased reactive loading of the bus.*

MLX90615 is always a slave device on the bus. MLX90615 can work in both low-power and high-power SMBus communication.

All voltages are with respect to the Vss (ground) unless otherwise noted.

8 Detailed description

8.1 Block diagram

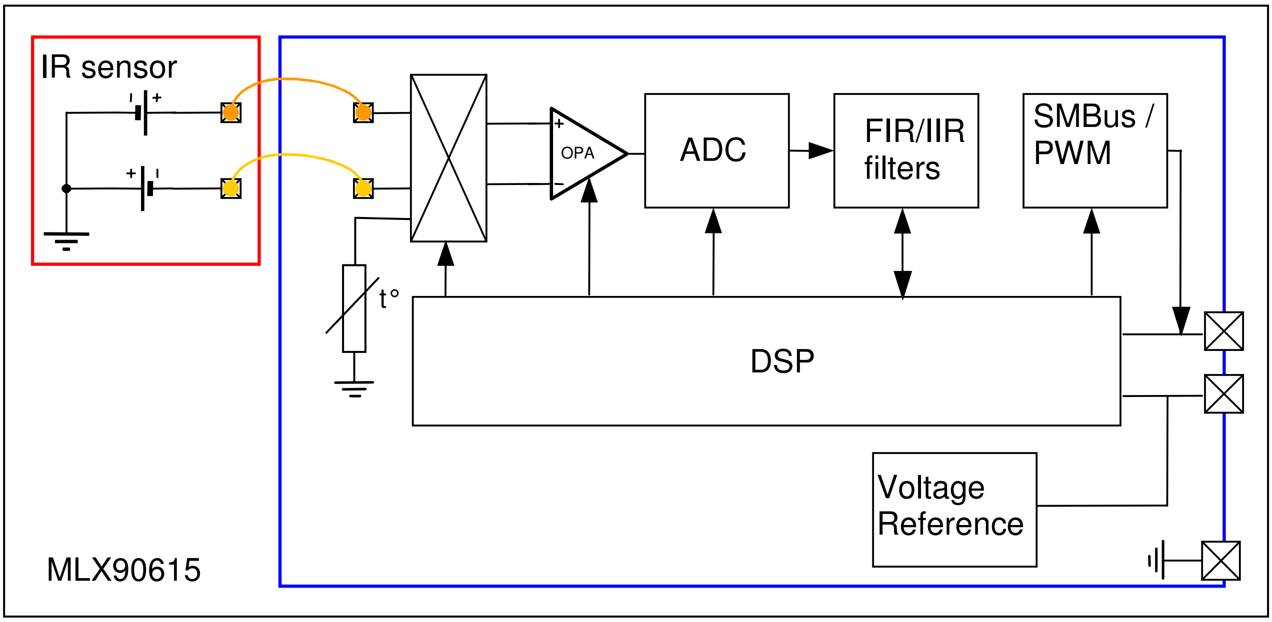

Figure 3: Block Diagram

8.2 Signal processing principle

A DSP embedded in the MLX90615 controls the measurements, calculates object and ambient temperatures and does the post-processing of the temperatures to output them through SMBus compatible interface or PWM (whichever activated).

The output of the IR sensor is amplified by a low noise, low offset chopper amplifier with programmable gain, then converted by a Sigma Delta modulator to a single bit stream and fed to the DSP for further processing. The signal passes a FIR low pass filter with fixed length of 65536. The output of the FIR filter is the measurement result and is available in the internal RAM. Based on results of the above measurements, the corresponding ambient temperature T_A and object temperatures T_O are calculated. Both calculated temperatures have a resolution of 0.02 ˚C.

An additional IIR LPF is programmable in EEPROM and allows customization of the thermometer output in order to trade-off noise versus settling time (refresh rate of the data in the RAM remain constant).

The IIR filter can also limit effect of spurious objects that may appear in the FOV in some applications.

The PWM output can be enabled in EEPROM as the POR default. Linearized temperatures (T_O or T_A, selectable in EEPROM) are available through the free-running PWM output.

8.3 Block description

8.3.1 Amplifier

A low noise, low offset amplifier with programmable gain is used for amplifying the IR sensor voltage. By a careful design of the input modulator and balanced input impedance, the max offset of the system is

0.5µV. As the device is factory calibrated any change of gain settings would require new calibration of object channel.

8.3.2 Power-On-Reset (POR)

The Power On Reset (POR) is connected to the Vdd supply. The on-chip POR circuit provides an active level of the POR signal when the Vdd voltage rises above approximately 0.5V and holds the entire MLX90615 in reset until the Vdd is higher than the specified POR threshold V_{POR}. During the time POR is active, the POR signal is available as a weak open drain (active high) at the SDA pin. After the MLX90615 exits the POR state, the functions programmed in the EEPROM take control of that pin.

8.3.3 EEPROM

A limited number of addresses in the EEPROM memory are of interest for the customer. The whole EEPROM can be read and written with the SMBus interface. The entire EEPROM content between addresses 0x04 and 0x0D must be kept unaltered or the factory calibration of the device will be lost.

EEPROM (16X16)		
Name	Address	Write access
PWM T min / SMBus slave address (SA)	0x00	Yes
PWM T range	0x01	Yes
Config	0x02	Yes
Emissivity	0x03	Yes
Melexis reserved (factory calibration)	0x04	Yes
…	…	…
Melexis reserved (factory calibration)	0x0D	Yes
ID number	0x0E	No
ID number	0x0F	No

Table 6 EERPOM table

SMBus slave address: 7 LSBs (6...0) contains the SMBus slave address that the MLX90615 will respond to. Note that all MLX90615 will respond to SA=0x00 and therefore this value is useless in a network. Factory default SA is 0x5B, max 127 devices on one line SA=0x01 …0x7F

PWM T min: 15 bit for the minimum temperature when PWM is used – right justified (factory default is 0x355B, which corresponds to +0.03℃)

PWM T range: 15 bit range for the PWM signal temperature ($T_{MAX} – T_{MIN}$) – right justified (factory default is 0x09C4, which corresponds to a PWM range of 50.01℃).

The **Config** register consist of control bits to configure the thermometer after POR:

15	14	13	12	11	10	9	8	7	6	5	4	3	2	1	0	Config register bit meaning (0x02)
															0	- Comminucation type is PWM
															1	- Comminucation type is SMBus
														0		- PWM frequency = 1KHz
														1		- PWM frequency = 10KHz
													0			- PWM output = To
													1			- PWM output = Ta
																- 0…63 - Internal RC oscillator trimnming
																- MLXreserved - DO NOT alter
					0	0										- GAIN = 1 - Amplifier bypassed MLX reserved - DO NOT alter
					0	1										- GAIN = 10 MLX reserved - DO NOT alter
					1	0										- GAIN = 40 MLX reserved - DO NOT alter
					1	1										- GAIN = 40 MLX reserved - DO NOT alter
				0												- Internal shunt regulatior disabled
				1												- Internal shunt regulatior enabled
	0	0	0													**- FORBIDDEN COMBINATION**
	0	0	1													- IIR (2) a1=1, b1=0
	0	1	0													- IIR (3) a1=0.5, b1=0.5
	0	1	1													- IIR (4) a1=0.333(3), b1=0.666(6)
	1	0	0													- IIR (5) a1=0.25, b1=0.75
	1	0	1													- IIR (6) a1=0.2, b1=0.8
	1	1	0													- IIR (7) a1=0.166(6), b1=0.833(3)
	1	1	1													- IIR (8) a1=0.14286, b1=0.87514
0																MLX reserved - DO NOT alter

Table 7 Config register meaning of the MLX90615 (continued)

**Note: IIR setting 000b must be avoided*

Emissivity: Contains the value for object emissivity correction. The MLX90615 will compensate for the emissivity of the object measured with respect to that value. The equation for that register is

$$Emissivity = dec2hex(round(16384 \times \varepsilon))$$

Where $dec2hex(round(x))$ represents decimal to hexadecimal conversion with round-off to nearest value (not truncation). In this case the physical emissivity values are **ε** 0…1. For details about the emissivity factor in IR measurements refer to the FAQ section of the current document.
Factory default is 0x4000, which sets the thermometer to an emissivity of 1.0 (emissivity correction is off).

8.3.4 RAM

RAM can be read through SMBus interface. Limited numbers of RAM registers, summarized below are of interest to the customer.

RAM (16x16)		
Name	Address	Read access
**Melexis reserved**	**0x00**	**Yes**
**…**	**…**	**…**
**Melexis reserved**	**0x04**	**Yes**
**Raw IR data**	**0x05**	**Yes**
**T_A**	**0x06**	**Yes**
**T_O**	**0x07**	**Yes**
**Melexis reserved**	**0x08**	**Yes**
**…**	**…**	**…**
**Melexis reserved**	**0x0F**	**Yes**

Table 8 RAM address list of the MLX90615

T_A is the MLX90615 package (ambient) temperature and T_O is the object temperature. The output scale is 0.02°K/LSB. To convert a read object temperature into degrees Celsius the equation is:

$$T_O[°C] = RAM(0x07) \times 0.02 - 273.15$$

Raw IR data is in sign (1 bit, the MSB) and magnitude (15 bits) format.

8.4 SMBus compatible 2-wire protocol

The chip supports a 2 wires serial protocol, build with pins SDA and SCL.

- SCL – digital input, used as the clock for SMBus compatible communication. A low pulse on that pin with duration t_{REQ} switches to the SMBus mode in case the PWM is selected in EEPROM. In case PWM operation is desired, the SCL pin should be kept high. SMBus is the factory default (via EEPROM settings).
- SDA / PWM – Digital input / NMOS open drain output, used for PWM and input / output for the SMBus. (SMBus is factory default function).

8.4.1 Functional description

The SMBus interface is a 2-wire protocol, allowing communication between the Master Device (MD) and one or more Slave Devices (SD). In the system only one master can be present at any given time [1]. The MLX90615 can only be used as a slave device.

Generally, the MD initiates the start of data transfer by selecting a SD through the Slave Address (SA).

The MD has read access to the RAM and EEPROM and write access to 14 EEPROM cells (at addresses 0x00…0x0D). If the access to the MLX90615 is a read operation, it will respond with 16 data bits and 8 bit PEC only if its own SA, programmed in the internal EEPROM, is equal to the SA, sent by the master. The SA feature allows connecting up to 127 devices with 2 wires, unless the system has some of the specific features described in paragraph 5.2 of reference [1]. In order to provide access to any device or to assign an address to a SD before it is connected to the bus system, the communication must start with zero SA followed by low $R/\overline{W}$ bit. When this command is sent from the MD, the MLX90615 will always respond and will ignore the internal chip code information.

Note that <u>EEPROM addresses 0x04…0x0D contain the factory calibration parameters and should not be altered</u>.

Special care must be taken not to put two MLX90615 devices with the same SD addresses on the same bus as MLX90615 does not support ARP[1].
The MD can force the MLX90615 into low consumption mode "sleep mode".

8.4.2 Differences with the standard SMBus specification (reference [1])

There are eleven command protocols for the standard SMBus interface. The MLX90615 supports only two of them. Not supported commands are:

- Quick Command
- Byte commands - Sent Byte, Receive Byte, Write Byte and Read Byte
- Process Call
- Block commands – Block Write and Write-Block Read Process Call

Supported commands are:

- Read Word
- Write Word

8.4.3 Detailed description

The SDA pin of the MLX90615 can operate also as a PWM output, depending on the EEPROM settings. If PWM is enabled, after POR the SDA pin is directly configured as a PWM output. The PWM mode can be avoided and the pin can be restored to its Serial Data function by issuing SMBus request condition. If SMBus mode is set, after POR, the request does not have to be sent.

8.4.3.1 Bus Protocol

1	7	1	1	8	1	1
S	Slave Address	Wr	A	Data Byte	A	P

S	Start Condition
Sr	Repeated Start Condition
Rd	Read (bit value of 1)
Wr	Write (bit value of 0)
A	Acknowledge (this bit can be 0 for ACK and 1 for NACK)
P	Stop Condition
PEC	Packet Error Code
☐ (white)	Master-to-Slave
■ (grey)	Slave-to-Master

Figure 4: SMBus packet element key

After every 8 bits received by the SD an ACK/NACK is followed. When a MD initiates communication, it first sends the SA. Only the SD with recognized SA gives ACK, the rest will remain silent. If the SD does not ACK the MD should stop the communication and repeat the message. A NACK could be received after the PEC. This means that there is an error in the received message and the MD should try sending the message again. The PEC calculation includes all bits except the START, REPEATED START, STOP, ACK, and NACK bits. The PEC is a CRC-8 with polynomial X8+X2+X1+1. The Most Significant Bit of every byte is transmitted first.

8.4.3.1.1 Read Word (depending on the command – RAM or EEPROM)

1	7	1	1	8	1	1	7	1	1	
S	Slave Address	Wr	A	Command	A	Sr	Slave Address	Rd	A	………..

	8	1	8	1	8	1	1
………..	Data Byte Low	A	Data Byte High	A	PEC	A	P

Figure 5: SMBus read word format

8.4.3.1.2 Write Word (EEPROM only)

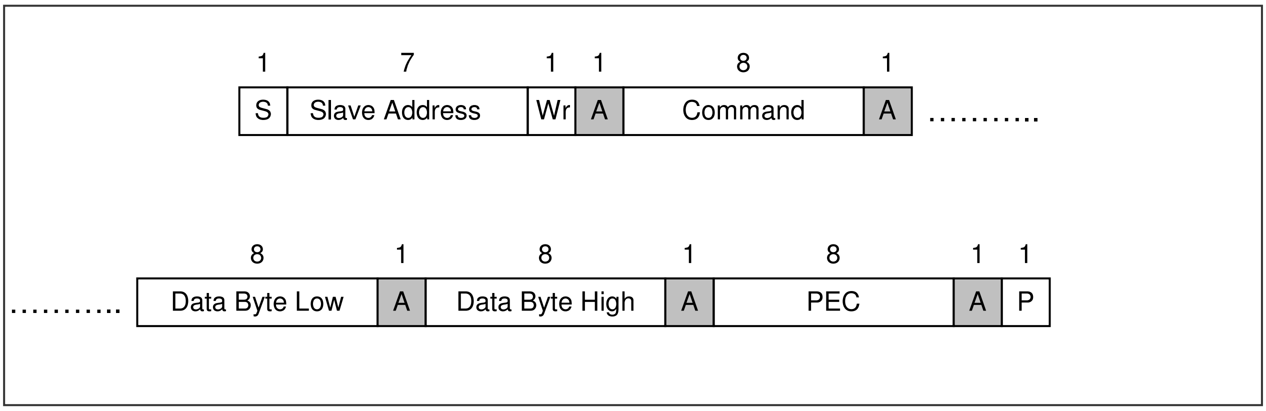

Figure 6: SMBus write word format

8.4.4 Bit transfer

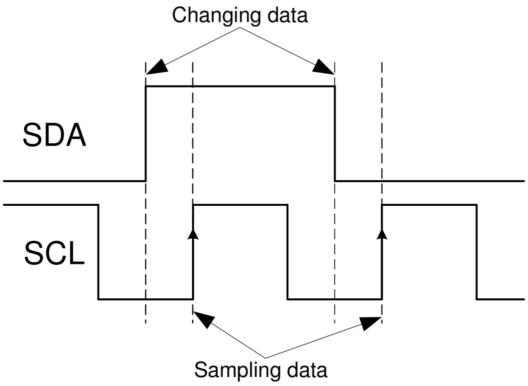

Figure 7: Bit transfer on SMBus

The data on SDA must be changed when SCL is low (min 300ns after the falling edge of SCL). The data is fetched by both MD and SDs on the rising edge of the SCL. The recommended timing for changing data is in the middle of the period when the SCL is low

8.4.5 Commands

In application mode RAM and EEPROM can be read both with 16x16 sizes. (For example, T_O - RAM address 0x07 will sweep between 0x2D8A to 0x4BD0 as the object temperature rises from -40℃ to +115℃). The MSB read from RAM is an error flag (active high) for the linearized temperatures (T_O and T_A).

Opcode	Command
0001 aaaa*	EEPROM Access
0010 aaaa*	RAM Access
1100 0110	Enter SLEEP mode

Table 9 Command list for the MLX90615

NOTE*: The aaaa are the 4 LSBits of the memory map address to be read / written

8.4.6 SMBus Communication examples

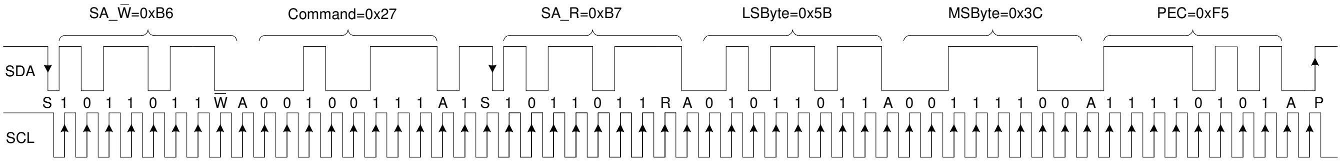

Figure 8: SMBus read word format (SA=0x5B, read RAM=0x07, result = 0x3C5B)

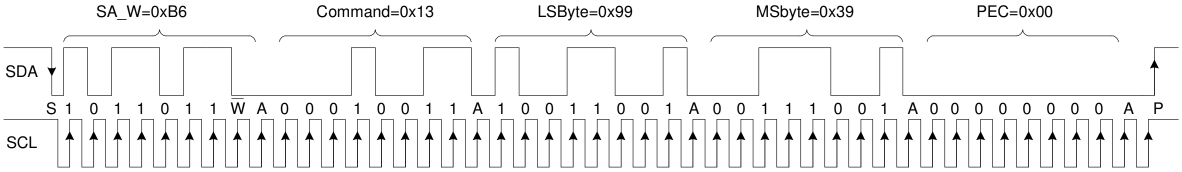

Figure 9: SMBus write word format (SA=0x5B, Write=0x3999 to EEPROM= 0x03)

Note: Before a write operation to takes place, the EEPROM cell needs to be erased. An erase operation is simply a writing of 0x0000 at the same EEPROM address. Care needs to be taken not to alter factory calibration (EEPROM addresses 0x04…0x0D).

8.4.7 Timing specification

The MLX90615 meets all the timing specifications of the SMBus [1] except the values given in the Electrical specifications section.The maximum frequency of the MLX90615 SMBus clock is 100 kHz and the minimum is 10 kHz.

The specific timings for the MLX90615's SMBus are:

SMBus Request (t_{REQ}) is the time that the SCL should be forced low in order to switch MLX90615 from thermal relay mode to SMBus mode – at least 39 ms

Timeout L is the maximum allowed time for SCL to be low. After this time the MLX90615 will reset its communication block and will be ready for new communication - not more than 21ms

Timeout H is the maximum time for which it is allowed for SCL to be high during communication. After this time MLX90615 will reset its communication block assuming that the bus is idle (according to the SMBus specification) - not more than 52µs

Tsuac(SD) is the time after the eighth falling edge of SCL during which the MLX90615 will force SDA low to acknowledge the last received byte – not more than 2.5µs

Thdac(SD) is the time after the ninth falling edge of SCL during which the MLX90615 will release the SDA (so the MD can continue with the communication) – not more than 1.5µs

Tsuac(MD) is the time after the eighth falling edge of SCL during which the MLX90615 will release SDA (so that the MD can acknowledge the last received byte) – not more than 1.5µs

Thdac(MD) is the time after the ninth falling edge of SCL during which the MLX90615 will take control of the SDA (so it can continue with the next byte to transmit) – not more than 1.5µs
The indexes MD and SD for the latest timings are used – MD when the master device is making the acknowledge; SD when the slave device is making the acknowledge). For other timings see [1].

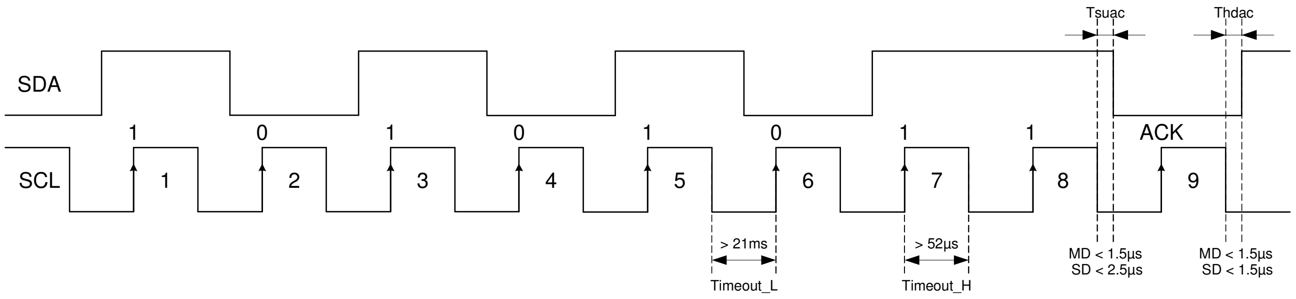

Figure 10: SMBus timing

8.4.8 Sleep Mode

Sleep mode is available in SMBus mode only.

MLX90615 can enter Sleep Mode via command “Enter SLEEP mode” sent via the SMBus interface. SCL needs to be high during Sleep. SDA can idle in each state at the same time, but the high state is recommended as the pull-up does not add current drain. There are weak pull-ups on both SCL and SDA pins.

8.4.8.1 Enter Sleep Mode

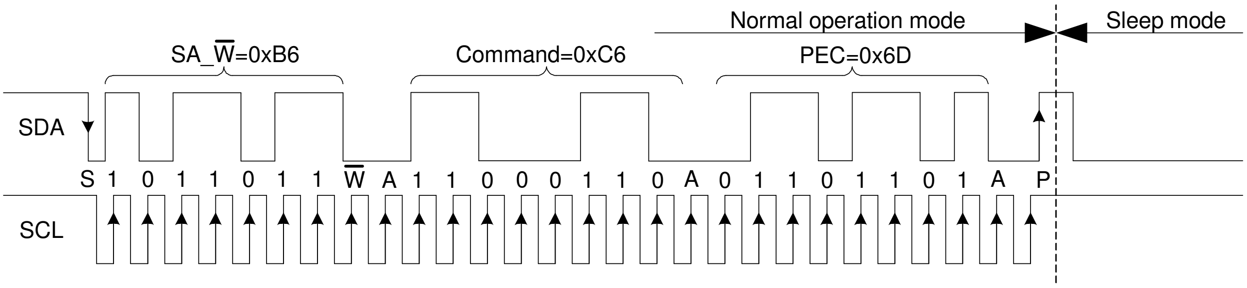

Figure 11: Enter sleep mode command (SA=0x5B, Command=0xC6,PEC=0x6D)

8.4.8.2 Exit Sleep Mode

MLX90615 goes back into power-up default mode by forcing the SCL pin low for at least t_{wake} > 8ms. Exit from Sleep is always in SMBus mode. Valid data will be available typically 0.3 seconds after the device has woken up.

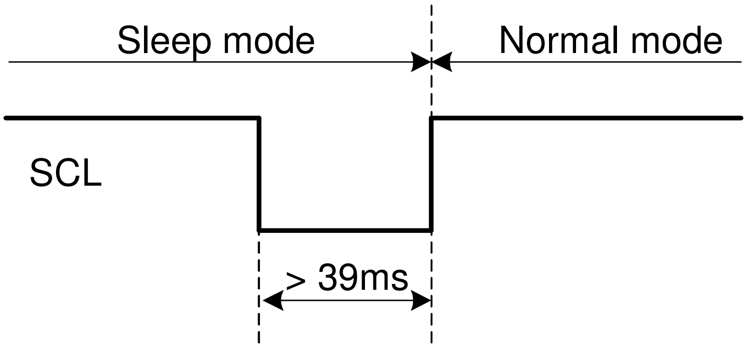

Figure 12 Exit Sleep Mode

8.5 Switching between PWM and SMBus

8.5.1 PWM to SMBus mode

The diagram below illustrates how to switch to SMBus if PWM is enabled. If PWM is enabled, the MLX90615's SMBus Request condition is needed to disable PWM and reconfigure SDA/PWM pin before starting SMBus communication. The MLX90615's SMBus request condition requires forcing the SCL pin LOW for a period longer than the request time (t_{REQ}>39ms). The SDA / PWM line value is ignored in this case. Note that after power ON / OFF the sensor will be in PWM mode if bit 0 from Config register is not set in SMBus mode.

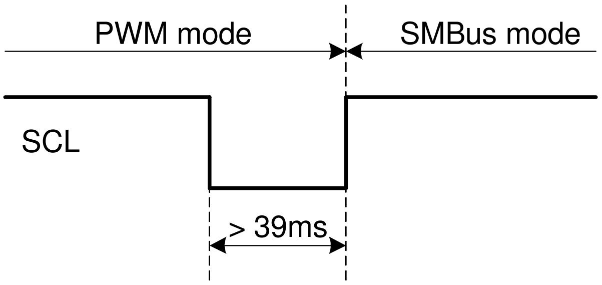

Figure 13: Switching from PWM to SMBus mode

8.5.2 SMBus to PWM mode

If SMBus mode is set by default, PWM mode can be set only by setting bit 0 from Config register to PWM mode and switching the supply OFF then ON.

8.6 PWM

The MLX90615 can be read via PWM or SMBus compatible interface. Selection of PWM output is done in EEPROM configuration (factory default is SMBus). Object or ambient temperature can be read through PWM. The PWM period is derived from the on-chip oscillator and is programmable in a low (10 Hz) or high (1 KHz) frequency mode.

Temperature ranges for the PWM output are determined by the contents of the cells 0x00, 0x01 in the EEPROM – PWM To_{MIN} and PWM_{RANGE} (To_{MAX} - To_{MIN}), scale is 0.02°K/LSB.

Note that in SMBus mode the EEPROM address 0x00 (LSByte) is used for Slave address SA.

Note that the SCL pin needs to be kept high in order to use the PWM function or to be left unconnected as there is a weak pull up build in.

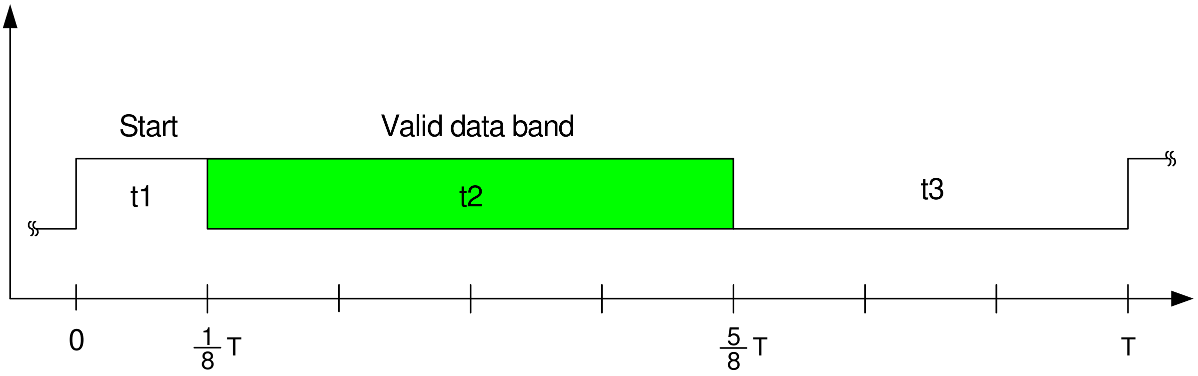

Figure 14 PWM format

8.6.1 PWM format

The temperature reading can be calculated from the signal timing as:

$$Tout = 2 \times \frac{t_2}{T} \times (T_{MAX} - T_{MIN}) + T_{MIN}$$

where Tmin and Trange are the corresponding rescale coefficients in EEPROM for the selected temperature output and T is the PWM period. Tout is T_O or T_A according to bit Config Register, 2.

The different time intervals t_1, t_2 and t_3 have the following functions:

t_1: Start buffer. During this time the signal is always high. $t_1 = 0.125 \times T$ (T is the PWM period, refer to fig. 14).

t_2 : Valid Data Output Band, 0 to 1/2T. PWM output data resolution is 10 bit.

t_3: always low signal.

The maximum duty cycle is limited to $t_1 + t_2 = 0.625$ this means that the PWM line will never go static, allowing detection of fault on the line (disconnected device, short on the line).

8.6.2 Customizing the temperature range for PWM output

The calculated ambient and object temperatures are stored in RAM with a resolution of 0.02°C (15 bit). The PWM operates with a 10-bit number so the transmitted temperature is rescaled in order to fit in the desired range.

For this goal 2 cells in EEPROM are foreseen to store the desired temperature range, PWM T_{MIN} and PWM T_{RANGE}.

Thus the output range can be programmed with an accuracy of 0.02 °C.

The data for PWM is rescaled according to the following equation:

$$T_{PWM} = \frac{T_{RAM} - T_{MIN_{EEPROM}}}{K_{PWM}}, K_{PWM} = \frac{T_{RANGE_{EEPROM}}}{1023}$$

The T_{RAM} is the linearized temperature, 15-bit (0x2D8A…0x4BD0, → -40 ..+115°C) and the result is a 10-bit word, in which 0x000 corresponds to PWM T_{MIN}[°C], 0x3FF corresponds to PWM T_{MAX}[°C] and 1LSB corresponds to:

$$1LSB = \frac{T_{MAX} - T_{MIN}}{1023}, [°C]$$

$$T_{MAX} = T_{MIN} + T_{RANGE}$$

$$T_{MIN_{EEPORM}} = T_{MIN} \times 50LSB$$

$$T_{MAX_{EEPORM}} = T_{MAX} \times 50LSB$$

Example:

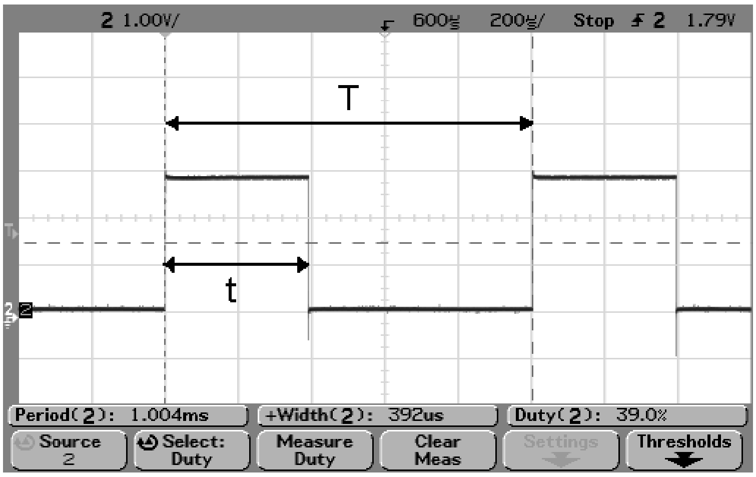

Figure 15: PWM example

$$To_{MIN} = 0°C \rightarrow To_{MIN}(\text{EEPROM}, 0x00) = 50 \times (To_{MIN} + 273.15) = 13658d = 0x355A$$

$$PWM_{RANGE}(To_{MAX} - To_{MIN}) = 50°C$$

$$PWM_{RANGE}(EEPROM, 0x01) = 50 \times PWM_{RANGE} \times (To_{MAX} - To_{MIN}) = 50 \times 50 = 2500d = 0x09C4$$

Captured PWM period is T = 1004µs (or frequency is 996 Hz i.e. high frequency mode is selected)
Captured high duration is t = 392 µs

Calculated duty cycle is:

$$D = \frac{t}{T} = \frac{392}{1004} = 0.3904 \text{ or } 39.04\%$$

The temperature is calculated as follows:

$$T_O = 2 \times (0.3904 - 0.125) \times (50 - 0) + 0 = 2 \times 0.2654 \times 50 = 26.54°C$$

Where 0.125 is START period (always high) which carry no temperature information and must be subtracted.

8.7 Principle of operation

The IR sensor consists of series connected thermo-couples with cold junctions placed at thick chip substrate and hot junctions, placed over thin membrane. The IR radiation absorbed from the membrane heats (or cools) it. The thermopile output signal is

$$V_{ir}(T_A, T_O) = A \times (T_O - T_A)$$

Where T_O is the object absolute temperature (Kelvin), T_A is the sensor die absolute (Kelvin) temperature, and A is the overall sensitivity.
An additional sensor is needed for the chip temperature. After measurement of the output of both sensors, the corresponding ambient and object temperatures can be calculated. These calculations are done by the internal DSP, which produces digital outputs, linearly proportional to measured temperatures.

8.7.1 Ambient temperature T_A

The Sensor die temperature is measured with a PTAT element. All the sensors conditioning and data processing is handled on-chip and the linearized sensor die temperature T_A is available in RAM (address 0x06).
The resolution of the calculated T_A is 0.02˚C. The sensor is factory calibrated for the range -20 … +85˚C.

Example:

$RAM(0x06) = 0x3171$ corresponding to -20.01˚C

$RAM(0x06) = 0x45F3$ corresponding to +84.99˚C

Conversion RAM content to real T_A is easy done with following expression:

$$T_A[°K] = 0.02 \times T_{A\_REG}$$

8.7.2 Object temperature T_O

The result has a resolution of 0.02˚C and is available in RAM (address 0x07). To is derived from RAM as:

$$T_O[°K] = 0.02 \times T_{O\_REG}$$

Example:

$RAM(0x07) = 0x2D89$ corresponding to -40.01˚C

$RAM(0x07) = 0x4BCF$ corresponding to +114.99˚C

Unique Features

The MLX90615 is a ready to use low-cost non contact thermometer provided by Melexis with output data linearly dependent on the object temperature with high accuracy and extended resolution.

The user can program the internal object emissivity correction for objects with a low emissivity.

The MLX90615 is housed in standard TO46 package.

The low power consumption and sleep mode make the thermometer ideally suited for handheld mobile applications.

The digital sensor interface can be either a PWM or an enhanced access SMBus compatible protocol. Systems with more than 100 devices can be built with only two signal lines.

9 Performance Graphs

9.1 Temperature accuracy of the MLX90615

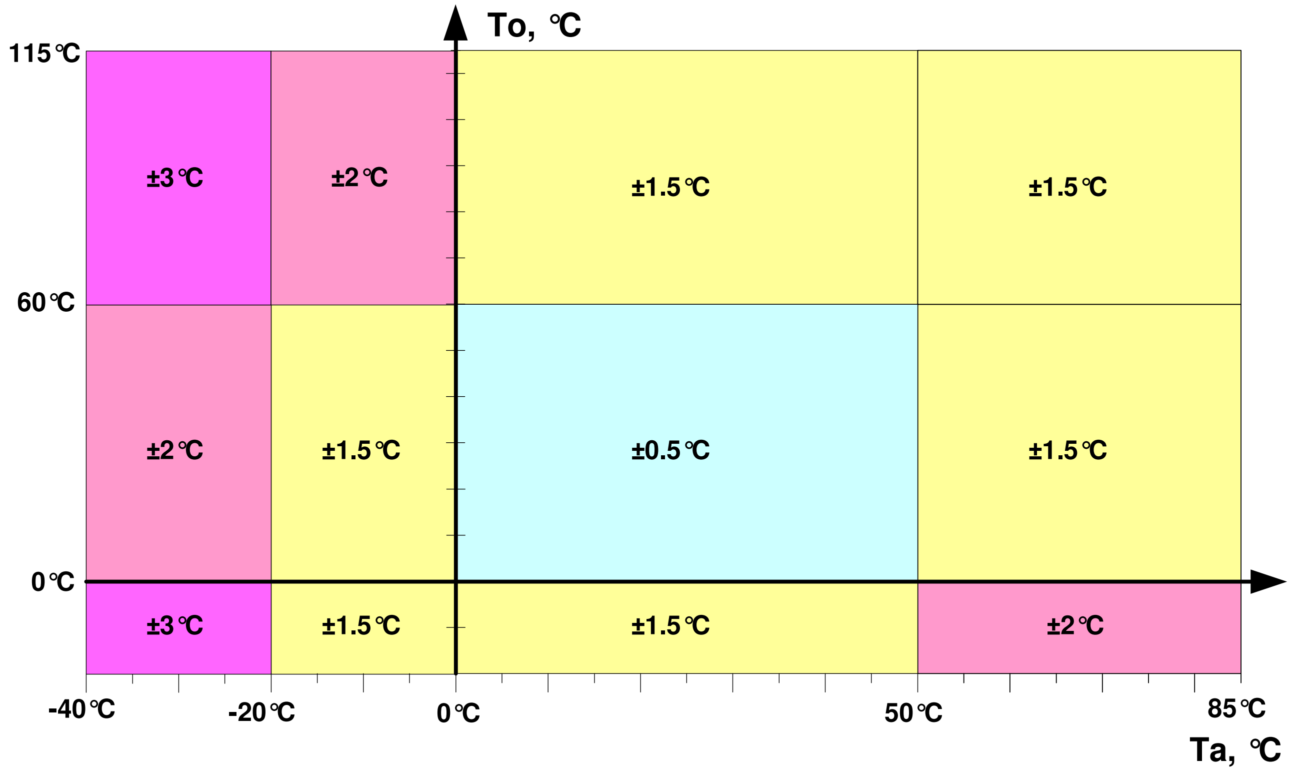

Figure 16: Accuracy of MLX90615 (T_A, T_O)

All accuracy specifications apply under settled isothermal conditions only and nominal supply voltage.

The accuracy for the MLX90615SSG-DAX in the range T_A =16°C …40°C and T_O= 32°C …42°C is shown in diagram below. The accuracy for the rest ranges is same as in previous diagram.

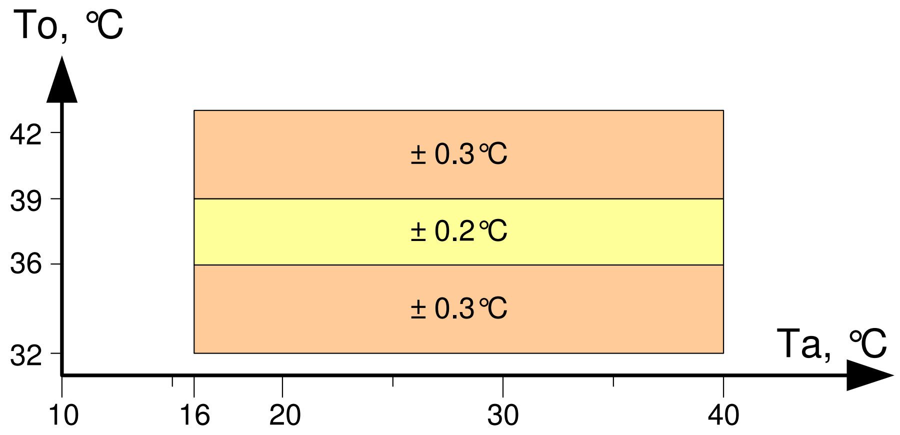

Figure 17: Accuracy of MLX90615SSG-DAx (T_A, T_O)

Versions MLX90615SSG-DAx comply with ASTM standard section 5.3 (Designation: E1965 – 98 (2009) - Standard Specification for Infrared Thermometers for Intermittent Determination of Patient Temperature).

9.2 Field Of View (FOV)

Field of view is determined at 50% thermopile signal and with respect to the sensor main axis.

Parameter	MLX90615SSG-XAA	MLX90615SSG-XAG
Peak direction	±0°	±0°
FOV width	100°±5°	80°±5°

Table 10 FOV options for the MLX90615

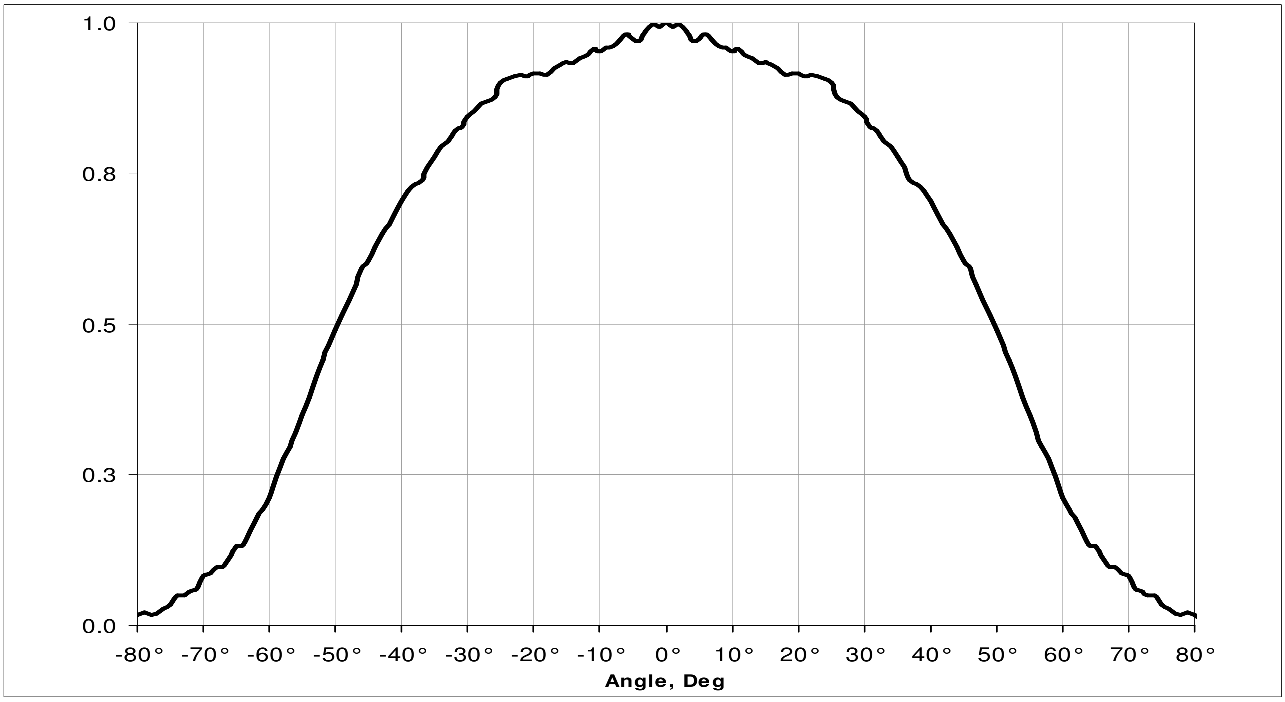

Figure 18: Typical FOV of MLX90615SSG-DAA

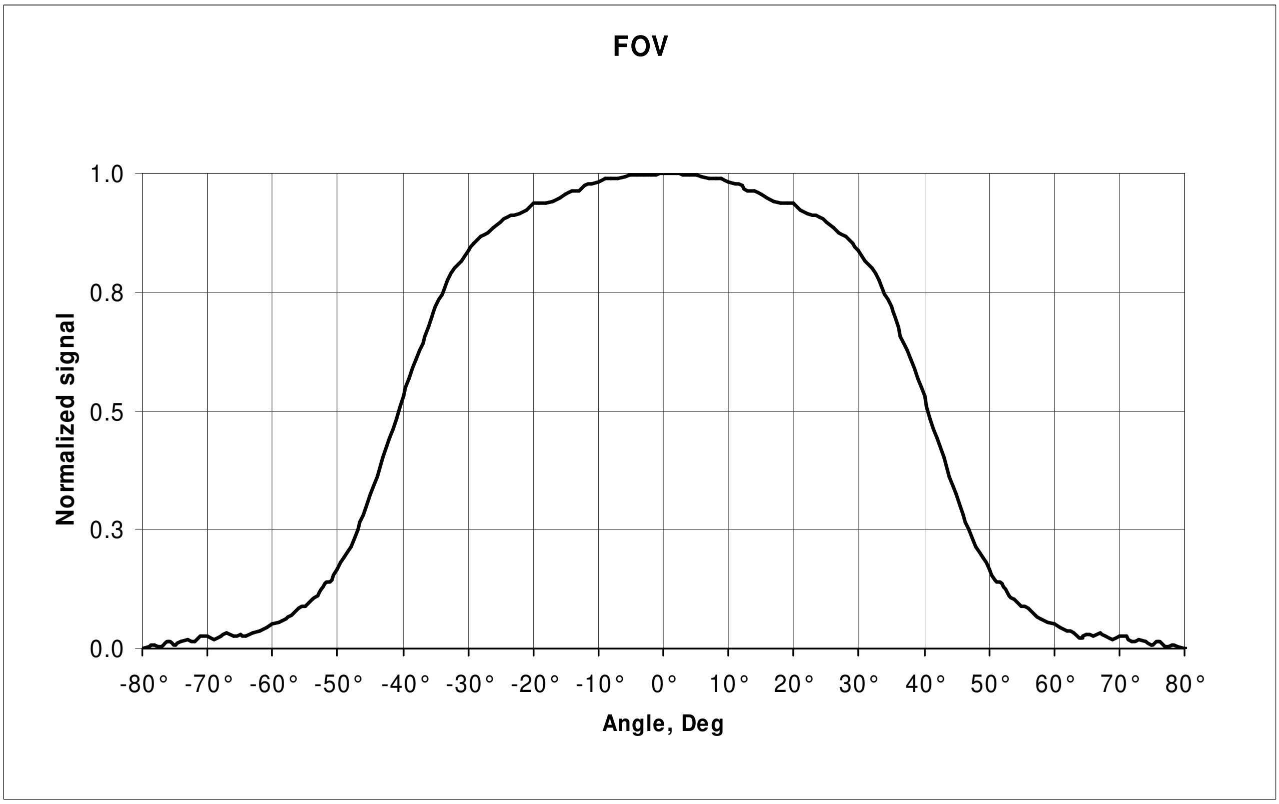

Figure 19: Typical FOV of MLX90615SSG-DAG

10 Applications Information

10.1 Use of the MLX90615 thermometer in SMBus configuration

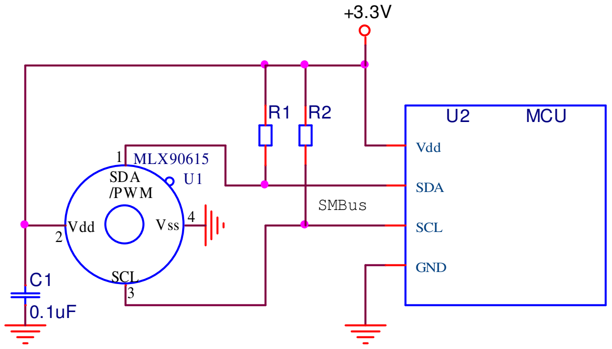

Figure 20: Connection of MLX90615 to SMBus.

MLX90615 has diode clamps SDA/SCL to Vdd so it is necessary to provide MLX90615 with power in order not to load the SMBus lines.

10.2 Use of multiple MLX90615s in SMBus configuration

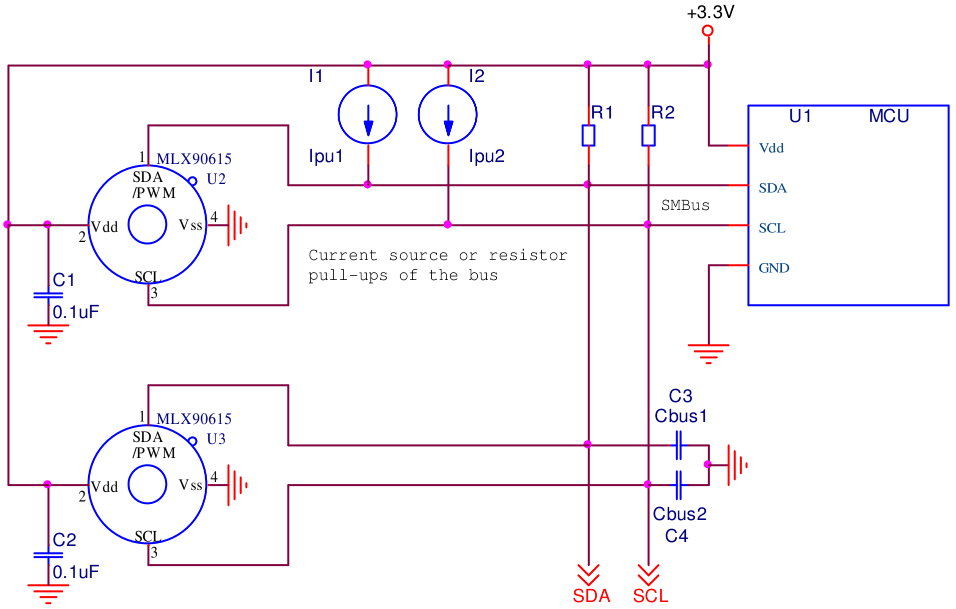

Figure 21: Use of multiple MLX90615 devices in SMBus network

The MLX90615 supports a 7-bit slave address in EEPROM, thus allowing up to 127 devices to be read via two common wires. Current source pull-ups may be preferred with higher capacitive loading on the bus (C3 and C4 represent the lines' parasitic), while simple resistive pull-ups provide the obvious low cost advantage.

10.3 PWM output

With PWM output configuration MLX90615 can be read via single wire. Output is open drain NMOS (with a weak pull-up, 300kΩ typ). Therefore external pull-up is required for high level state on the line with longer wires. Simple level shifting is possible with a single resistor. ESD protective clamp on the SDA pin consists of 4 diodes to Vdd, thus allowing high level to go up to 5V disregarding the MLX90615 supply voltage value.

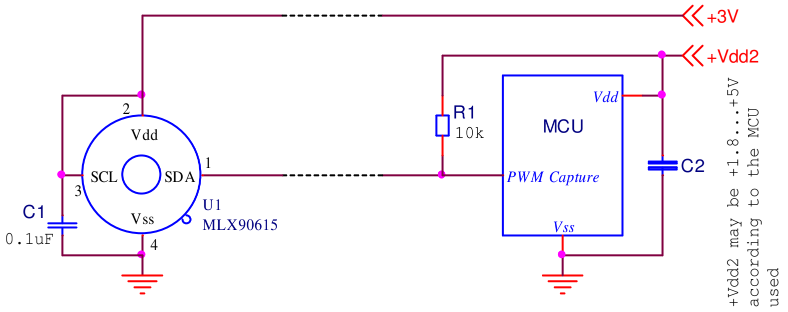

Figure 22: Using MLX90615 PWM output

In EEPROM two PWM periods can be programmed – 102.4 or 1 ms (typ). With remote installation (wires)

PWM is recommended as more robust to EMI than the SMBus and the high PWM period would be also preferred. As a factory default, once PWM is enabled, output will cover 0…50℃ object temperature range (as 12.5 … 62.5% duty cycle) at 1 kHz frequency.

11 Application Comments

Significant **contamination** at the optical input side (sensor filter) might cause unknown additional filtering/distortion of the optical signal and therefore result in unspecified errors.

IR sensors are inherently susceptible to errors caused by **thermal gradients**. There are physical reasons for that phenomenon and, in spite of the careful design of the MLX90615, it is recommended not to subject the MLX90615 to heat transfer and especially transient conditions.

Upon **power-up** the MLX90615 passes embedded checking and calibration routines. During these routines the output is not defined and it is recommended to wait for the specified POR time before reading the module. Very slow power-up may cause the embedded POR circuitry trigger on inappropriate levels, resulting in unspecified operation and is not recommended.

The MLX90615 is designed and calibrated to operate as a non contact thermometer in **settled conditions**. Using the module in very different way will result in unknown results.

Capacitive loading on a SMBus can degrade the communication. Some improvement is possible with use of current sources compared to resistors in pull-up circuitry. Further improvement is possible with specialized commercially available bus accelerators. With the MLX90615 additional improvement is possible with increasing the pull-up current (decreasing the pull-up resistor values). Input levels for SMBus compatible mode have higher overall tolerance than the SMBus specification, but the output low level is rather low even with the high-power SMBus specification for pull-up currents. Another option might be to go for a slower communication (clock speed), as the MLX90615 implements Schmidt triggers on it's inputs in SMBus compatible mode and is therefore not really sensitive to rise time of the bus (it is more likely the rise time to be an issue than the fall time, as far as the SMBus systems are open drain with pull-up).

For **ESD protection** there are clamp diodes between the Vss and Vdd and each of the other pins. This means that the MLX90615 might draw current from a bus in case the SCL and/or SDA is connected and the Vdd is lower than the bus pull-ups' voltage.

It is possible to use the MLX90615 in applications, powered directly from the AC line (trasformerless). In such cases it is very important not to forget that **the metal package of the sensor is not isolated** and therefore may occur to be connected to that line, too. Melexis can not be responsible for any application like this and highly recommends not using the MLX90615 in that way.

Power supply decoupling capacitor is needed as with most integrated circuits. MLX90615 is a mixed-signal device with sensors, small signal analog part, digital part and I/O circuitry. In order to keep the noise low power supply switching noise needs to be decoupled. High noise from external circuitry can also affect noise performance of the device. In many applications a 100nF SMD ceramic capacitor close to the Vdd and Vss pins would be a good choice. It should be noted that not only the trace to the Vdd pin needs to be short, but also the one to the Vss pin. Using MLX90615 with short pins improves the effect of the power supply decoupling.
Severe noise can also be coupled within the package from the SCL (in worst cases also from the SDA) pin. This issue can be solved by using PWM output. Also the PWM output can pass additional filtering (at lower PWM frequency settings). With a simple LPF RC network added also increase of the ESD rating is possible.

Check www.melexis.com for most current application notes about MLX90615.

12 Standard information regarding manufacturability of Melexis products with different soldering processes

Our products are classified and qualified regarding soldering technology, solderability and moisture sensitivity level according to following test methods:

Reflow Soldering SMD's (Surface Mount Devices)

- IPC/JEDEC J-STD-020
  Moisture/Reflow Sensitivity Classification for No hermetic Solid State Surface Mount Devices
  (classification reflow profiles according to table 5-2)
- EIA/JEDEC JESD22-A113
  Preconditioning of Nonhermetic Surface Mount Devices Prior to Reliability Testing
  (reflow profiles according to table 2)

Wave Soldering SMD's (Surface Mount Devices) and THD's (Through Hole Devices)

- EN60749-20
  Resistance of plastic- encapsulated SMD's to combined effect of moisture and soldering heat
- EIA/JEDEC JESD22-B106 and EN60749-15
  Resistance to soldering temperature for through-hole mounted devices

The application of Wave Soldering for SMD's is allowed only after consulting Melexis regarding assurance of adhesive strength between device and board!

Iron Soldering THD's (Through Hole Devices)

- EN60749-15
  Resistance to soldering temperature for through-hole mounted devices

Solderability SMD's (Surface Mount Devices) and THD's (Through Hole Devices)

- EIA/JEDEC JESD22-B102 and EN60749-21
  Solderability

For all soldering technologies deviating from above mentioned standard conditions (regarding peak temperature, temperature gradient, temperature profile etc) additional classification and qualification tests have to be agreed upon with Melexis.

General precautions for soldering, handling or cleaning:

- Thermal Stresses above the absolute maximum ratings during application or storage may cause damages to the device.
- Do not expose the sensor to aggressive detergents such as Freon, Trichloroethylene, etc.
- Windows may be cleaned with alcohol and cotton swab at ambient temperatures.
- Rework by manual soldering can be done with soldering iron temperature maximum 350°C for maximum 5 sec.
- Avoid heat exposure to the top and the window of the package.
- Do not apply pressure (force) on the window or top of the package, especially when T_A temperature is above 85°C.
- Temperatures above absolute maximum rating and/or mechanical pressure on the window or top of the package may cause loss of hermeticity and as a result penetration of a humid air inside the package, bond pad and wire corrosion, leading to degradation or loss of function.

- When considering reflow soldering, measure the temperature profile of the top of the can and keep the peak temperature as low as possible.
- Please contact Melexis in case you intend to use a reflow soldering process for through hole devices to verify your soldering process design.

Melexis is contributing to global environmental conservation by promoting **lead free** solutions. For more information on qualifications of **RoHS** compliant products (RoHS = European directive on the Restriction Of the use of certain Hazardous Substances) please visit the quality page on our website: http://www.melexis.com/quality.asp

The MLX90615 is RoHS compliant

13 ESD Precautions

Electronic semiconductor products are sensitive to Electro Static Discharge (ESD).
Always observe Electro Static Discharge control procedures whenever handling semiconductor products.

14 FAQ

When I measure aluminium and plastic parts settled at the same conditions I get significant errors on aluminium. Why?

Different materials have different **emissivity**. A typical value for aluminium (roughly polished) is 0.18 and for plastics values of 0.84…0.95 are typical. IR thermometers use the radiation flux between the sensitive element in the sensor and the object of interest, given by the equation

$$q = \varepsilon_1 \times \alpha_1 \times \left(T_1^{\,4}\right) \times \sigma \times A_1 \times F_{a-b} - \varepsilon_2 \times \left(T_2^{\,4}\right) \times \sigma \times A_2 \,,$$

Where
ε_1 and **ε_2** are the emissivity of the two objects,
α_1 is the absorptivity of the sensor (in this case),
σ is the Stefan-Boltzmann constant,
A_1 and **A_2** are the surface areas involved in the radiation heat transfer,
F_{a-b} is the shape factor,
T_1 and **T_2** are known temperature of the sensor die (measured with specially integrated and calibrated element) and the object temperature that we need.
Note that these are all in Kelvin, heat exchange knows only physics.

When a body with low emissivity (such as aluminium) is involved in this heat transfer, the portion of the radiation incident to the sensor element that really comes from the object of interest decreases – and the reflected environmental IR emissions take place. (This is all for bodies with zero transparency in the IR band.)

The IR thermometer is calibrated to stay within specified accuracy – but it has no way to separate the incoming IR radiation into real object and reflected environmental part. Therefore, measuring objects with low emissivity is a very sophisticated issue and infra-red measurements of such materials is a specialised field.

What can be done to solve that problem? Look at paintings – for example, oil paints are likely to have emissivity of 0.85…0.95 – but keep in mind that the stability of the paint emissivity has inevitable impact on measurements.

It is also a good point to keep in mind that not everything that looks black is “black” also for IR. For example, even heavily oxidized aluminium has still emissivity as low as 0.30.

How high is enough? Not an easy question – but, in all cases the closer you need to get to the real object temperature the higher the needed emissivity will be, of course.

With the real life emissivity values the environmental IR comes into play via the reflectivity of the object (the sum of Emissivity, Reflectivity and Absorptivity gives 1.00 for any material). The larger the difference between environmental and object temperature is at given reflectivity (*with an opaque for IR material reflectivity equals 1.00 minus emissivity*) the bigger errors it produces.

After I put the MLX90615 in the dashboard I start getting errors larger than specified in spite that the module was working properly before that. Why?

Any object present in the FOV of the module provides IR signal. It is actually possible to introduce error in the measurements if the module is attached to the dashboard with an opening that enters the FOV. In that case portion of the dashboard opening will introduce IR signal in conjunction with constraining the effective FOV and thus compromising specified accuracy. Relevant opening that takes in account the FOV is a must for accurate measurements. Note that the basic FOV specification takes 50% of IR signal as threshold (in order to define the area, where the measurements are relevant), while the entire FOV at lower level is capable of introducing lateral IR signal under many conditions.

When a hot (cold) air stream hits my MLX90615 some error adds to the measured temperature I read. What is it?

IR sensors are inherently sensitive to difference in temperatures between the sensitive element and everything incident to that element. As a matter of fact, this element is not the sensor package, but the sensor die inside. Therefore, a thermal gradient over the sensor package will inevitably result in additional IR flux between the sensor package and the sensor die. This is real optical signal that can not be segregated from the target IR signal and will add errors to the measured temperature.

Thermal gradients with impact of that kind are likely to appear during transient conditions. The sensor used is developed with care about sensitivity to this kind of lateral phenomena, but their nature demands some care when choosing place to use the MLX90615 in order to make them negligible.

I measure human body temperature and I often get measurements that significantly differ from the +37°C I expect.

IR measurements are true surface temperature measurements. In many applications this means that the actual temperature measured by an IR thermometer will be temperature of the clothing and not the skin temperature. Emissivity (explained first in this section) is another issue with clothes that has to be considered. There is also the simple chance that the measured temperature is adequate – for example, in a cold winter human hand can appear at temperatures not too close to the well known +37°C.

I consider using MLX90615 to measure temperature within car compartment, but I am embarrassed about the Sun light that may hit the module. Is it a significant issue?

Special care is taken to cut off the visible light spectra as well as the NIR (near IR) before it reaches the sensitive sensor die. Even more, the glass (in most cases) is not transparent to the IR radiation used by the MLX90615. Glass has temperature and really high emissivity in most cases – it is “black” for IR of interest. Overall, Sun behind a window is most likely to introduce relatively small errors. Why it is not completely eliminated after all? Even visible light partially absorbed in the filter of the sensor has some heating potential – and there is no way that the sensor die will be “blind” for that heating right in front of it.

15 Package Information

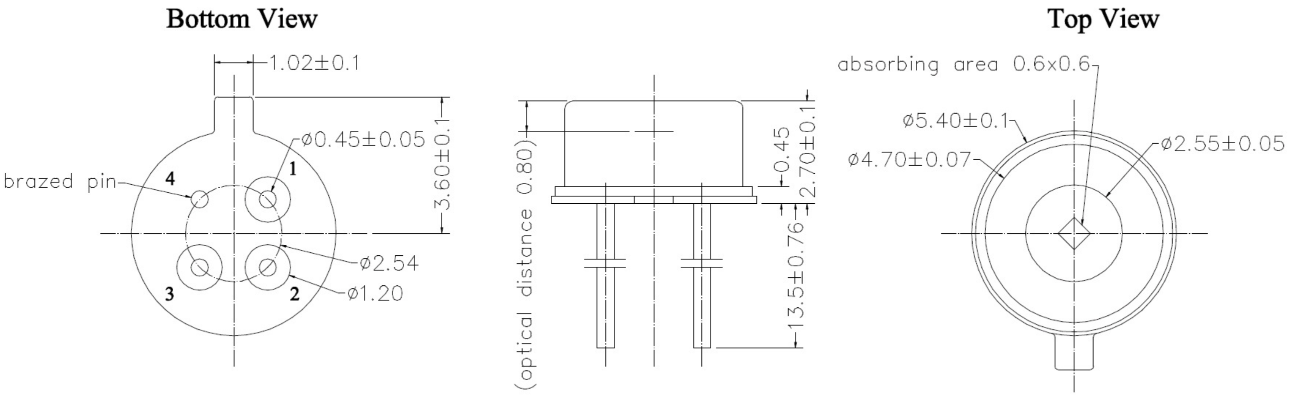

Figure 23: MLX90615SSG-DAA package drawing

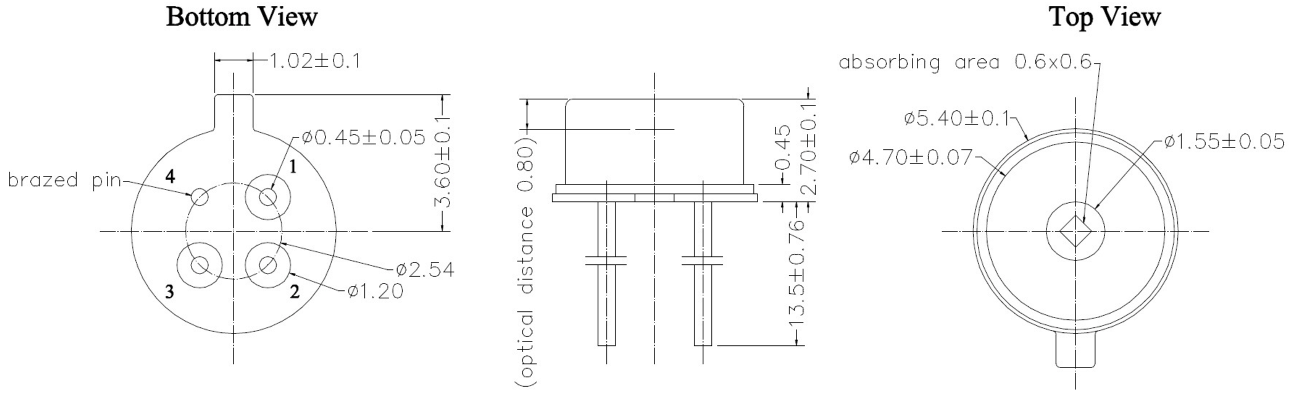

Figure 24: MLX90615SSG-DAG package drawing

16 Part marking

No part marking is foreseen for this product

17 Table of figures

Figure 1 Typical application schematics – MLX90615 connection to SMBus....1
Figure 2: Pin description MLX90615....5
Figure 3: Block Diagram....8
Figure 4: SMBus packet element key....12
Figure 5: SMBus read word format....12
Figure 6: SMBus write word format....13
Figure 7: Bit transfer on SMBus....13
Figure 8: SMBus read word format (SA=0x5B, read RAM=0x07, result = 0x3C5B)....14
Figure 9: SMBus write word format (SA=0x5B, Write=0x3999 to EEPROM= 0x03)....14
Figure 10: SMBus timing....15
Figure 11: Enter sleep mode command (SA=0x5B, Command=0xC6,PEC=0x6D)....15
Figure 12 Exit Sleep Mode....15
Figure 13: Switching from PWM to SMBus mode....16
Figure 14 PWM format....16
Figure 15: PWM example....18
Figure 16: Accuracy of MLX90615 (T_A, T_O)....20
Figure 17: Accuracy of MLX90615SSG-DAx (T_A, T_O)....21
Figure 18: Typical FOV of MLX90615SSG-DAA....21
Figure 19: Typical FOV of MLX90615SSG-DAG....22
Figure 20: Connection of MLX90615 to SMBus.....23
Figure 21: Use of multiple MLX90615 devices in SMBus network....23
Figure 22: Using MLX90615 PWM output....24
Figure 23: MLX90615SSG-DAA package drawing....29
Figure 24: MLX90615SSG-DAG package drawing....29

18 References

[1] **System Management Bus (SMBus) Specification** Version 2.0 August 3, 2000
SBS Implementers Forum Copyright . 1994, 1995, 1998, 2000
Duracell, Inc., Energizer Power Systems, Inc., Fujitsu, Ltd., Intel Corporation, Linear Technology Inc., Maxim Integrated Products, Mitsubishi Electric Semiconductor Company, PowerSmart, Inc., Toshiba Battery Co. Ltd., Unitrode Corporation, USAR Systems, Inc.

19 Disclaimer

Devices sold by Melexis are covered by the warranty and patent indemnification provisions appearing in its Term of Sale.

Melexis makes no warranty, express, statutory, implied, or by description regarding the information set forth herein or regarding the freedom of the described devices from patent infringement. Melexis reserves the right to change specifications and prices at any time and without notice. Therefore, prior to designing this product into a system, it is necessary to check with Melexis for current information. This product is intended for use in normal commercial applications. Applications requiring extended temperature range, unusual environmental requirements, or high reliability applications, such as military, medical life-support or life-sustaining equipment are specifically not recommended without additional processing by Melexis for each application.

The information furnished by Melexis is believed to be correct and accurate. However, Melexis shall not be liable to recipient or any third party for any damages, including but not limited to personal injury, property damage, loss of profits, loss of use, interrupt of business or indirect, special incidental or consequential damages, of any kind, in connection with or arising out of the furnishing, performance or use of the technical data herein. No obligation or liability to recipient or any third party shall arise or flow out of Melexis' rendering of technical or other services.

© 2006 Melexis NV. All rights reserved.

For the latest version of this document, go to our website at
www.melexis.com

Or for additional information contact Melexis Direct:

Europe, Africa, Asia:
Phone: +32 1367 0495
E-mail: sales_europe@melexis.com

America:
Phone: +1 603 223 2362
E-mail: sales_usa@melexis.com

ISO/TS 16949 and ISO14001 Certified

Mouser Electronics

Authorized Distributor

Click to View Pricing, Inventory, Delivery & Lifecycle Information:

Melexis:

MLX90615SSG-DAG-000-TU MLX90615SSG-DAA-000-SP MLX90615SSG-DAG-000-SP MLX90615SSG-DAA-000-TU
